# Design, Synthesis and Antiviral Evaluation of Pyrido[1,2-*c*]pyrimidin-1-one Derivatives Against Porcine Epidemic Diarrhea Virus (PEDV)

**DOI:** 10.3390/molecules31091480

**Published:** 2026-04-29

**Authors:** Wenlong Xu, Wu Ni, Ziyan Zhou, Zhenhui Ju, Sisi Liu, Shixiang Pan, Xiangrui Jiang

**Affiliations:** 1 School of Pharmacy, Anhui University of Chinese Medicine, Hefei 230012, China; wlxu@baridd.ac.cn; 2 Shandong Laboratory of Yantai Drug Discovery, Bohai Rim Advanced Research Institute for Drug Discovery, Yantai 264117, China; zyzhou@baridd.ac.cn (Z.Z.); zhju@baridd.ac.cn (Z.J.); 3 College of Chemistry, Huazhong Agricultural University, Wuhan 430070, China; wuni@webmail.hzau.edu.cn; 4 Key Laboratory of Structure-Based Drug Design and Discovery of Ministry of Education, Shenyang Pharmaceutical University, Shenyang 110016, China; 5 Shanghai Institute of Materia Medica, Chinese Academy of Sciences, Shanghai 201203, China; 6 University of Chinese Academy of Sciences, Beijing 100049, China

**Keywords:** PEDV, pyrido[1,2-*c*]pyrimidin-1-one (PPO) derivatives, antiviral, SAR

## Abstract

Porcine epidemic diarrhea virus (PEDV) is the pathogen responsible for porcine epidemic diarrhea, causing significant economic losses to the swine industry. During the replication of PEDV, the genome mutates rapidly, making the effectiveness of commercial vaccines uncertain when facing newly emerging prevalent variants. More importantly, there are currently no safe and effective specific antiviral drugs available. **YT1418**, a pyrido[1,2-*c*]pyrimidin-1-one (PPO) compound, exhibited anti-PEDV activity in a previous study. To expand the chemical space of the PPO scaffold and clarify the influence of substituents at different positions on the antiviral activity of the compounds, 36 new compounds were designed and synthesized, and then their abilities to inhibit viral replication in a PEDV-infected cell model were evaluated. Furthermore, the hepatic microsomal metabolic stabilities of compounds with potent antiviral activity were assessed. The results showed that compounds **N1** and **N2** exhibited antiviral activity (EC_50_ = 0.32, 0.37 μM, respectively) superior to that of **YT1418**, with selective index values of 43.78 and 42.89, respectively. Meanwhile, compound **J4** demonstrated good hepatic microsomal stability and low cytotoxicity, which requires further investigation. This study identified lead compounds featuring a novel PPO core and established their structure–activity relationships, providing important insights for the development of anti-PEDV drugs.

## 1. Introduction

Porcine epidemic diarrhea (PED) is a severe swine intestinal disease that was first reported in the UK in 1971 [1]. In 1978, its causative pathogen was identified and designated as porcine epidemic diarrhea virus (PEDV), a single-stranded positive-sense RNA virus belonging to the genus α-Coronavirus, with a diameter of 95–190 nm and typical crown-shaped surface morphology [2,3,4,5,6]. PEDV primarily infects piglets, with typical symptoms including vomiting, watery diarrhea, severe dehydration, anorexia and rapid weight loss. The incidence rate reaches 80–100% and mortality up to 50–90%, causing substantial economic losses to the swine industry [7,8,9,10,11,12,13,14]. The highly pathogenic GII PEDV variant, emerging in 2010, spread rapidly across Asia, North America and Europe [15,16,17,18,19]. Its genome is prone to replication-associated mutations, driving the constant emergence of new variants and compromising the immunoprotective efficacy and safety of traditional attenuated and inactivated vaccines [20,21,22,23]. Although next-generation vaccines including mRNA, viral vector and subunit vaccines are under development, they are not yet commercially available [24,25]. Given such limitations, the development of safe and effective novel small-molecule inhibitors against viral replication is of great importance [26,27].

**Figure 1:**
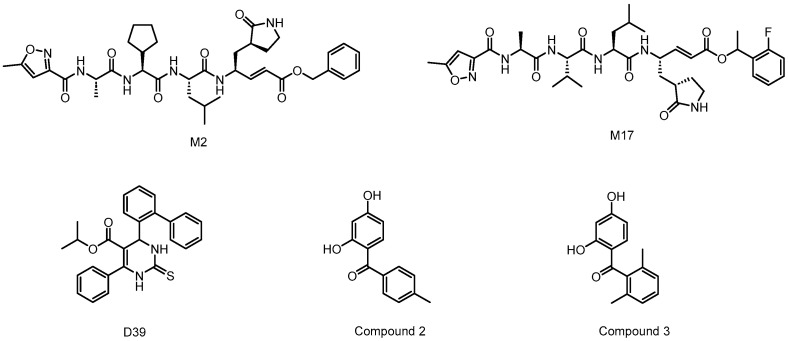
Representative Small-Molecule Inhibitors against PEDV.

Anti-PEDV drug development has made significant progress; in particular, small-molecule synthetic compounds have shown promise [28,29,30]. Several representative small-molecule inhibitors against porcine epidemic diarrhea virus (PEDV) that have been reported to date are shown in Figure 1. In 2017, Wang F. et al. optimized Michael acceptor-containing peptide inhibitors targeting PEDV M^pro^. Compounds **M2** and **M17** exhibited inhibition rates of 96% and 93% against PEDV M^pro^, respectively, demonstrating excellent inhibitory activity [31]. Lv S. et al. designed and synthesized a series of 3,4-dihydropyrimidine derivatives, with compound **D39** selected as the preferred candidate. It blocks early PEDV internalization by regulating calcium homeostasis, exhibiting an EC_50_ of 0.09 μM and a selectivity index (SI) of 358.9, exhibiting 35-fold higher activity than remdesivir. It demonstrates broad-spectrum inhibition against coronaviruses including FIPV and maintains metabolic stability in vitro [32]. Shi Y. et al. targeted the PEDV 3CL^pro^ and identified compound **2** and compound **3** as PEDV 3CL^pro^ inhibitors from a library of 1000 compounds, with the aforementioned compounds inhibiting PEDV replication with EC_50_ values of 100 μM and 57.9 μM, respectively [33].

**Figure 2:**
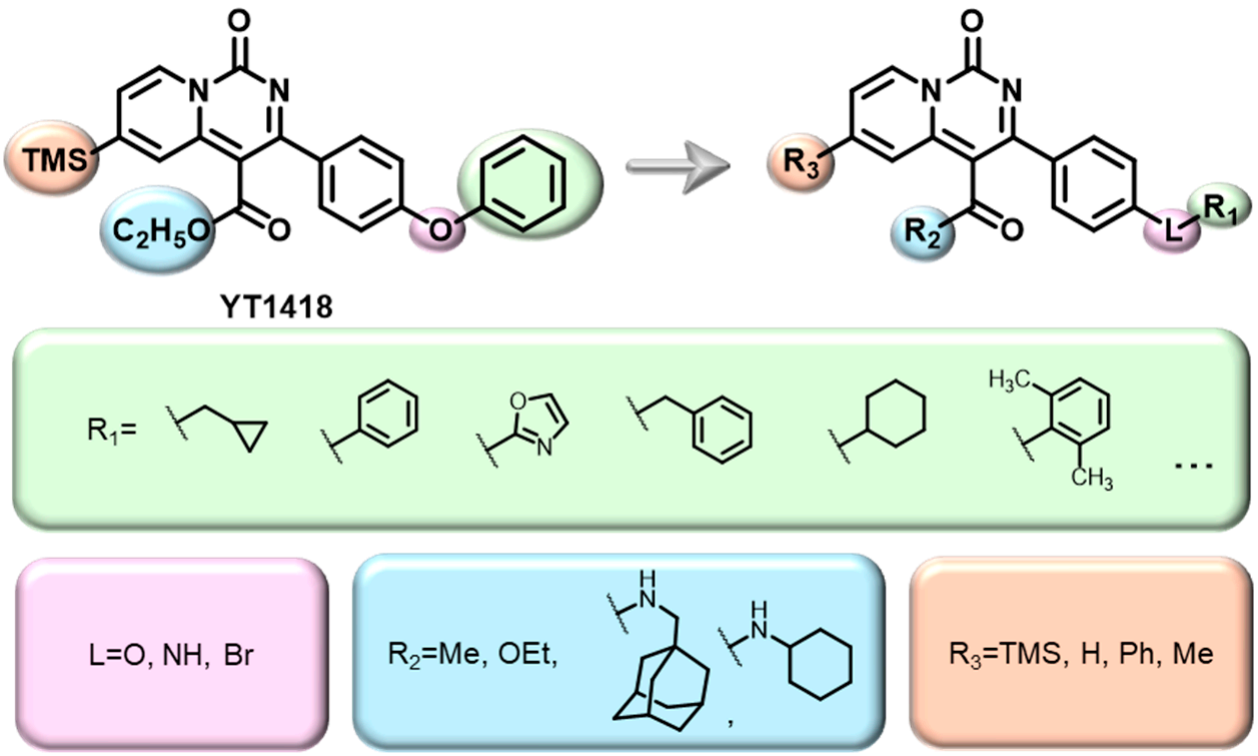
Design of PPO Derivatives.

A series of small-molecule compounds with promising anti-PEDV activity has been previously synthesized. Among them, compound **YT1418** (Figure 2) demonstrates the most potent antiviral activity against PEDV, with an EC_50_ value of 1.76 μM. This compound features a Pyrido[1,2-*c*]pyrimidin-1-one (PPO) core scaffold, which was initially constructed in the corresponding research group. To further extend the chemical space of this unique scaffold and identify more potent anti-PEDV agents, 36 derivatives bearing diverse substituents at the R_1_, R_2_, R_3_, and L positions of **YT1418** were designed and synthesized. The structures of all compounds are shown in Table S1. Their anti-PEDV activities and structure–activity relationships (SARs) were systematically evaluated. In addition, the hepatic microsomal metabolic stability of several representative derivatives was determined.

## 2. Results and Discussion

### 2.1. Chemical Synthesis and NMR Characterization

#### 2.1.1. Chemical Synthesis

**Scheme 1:**
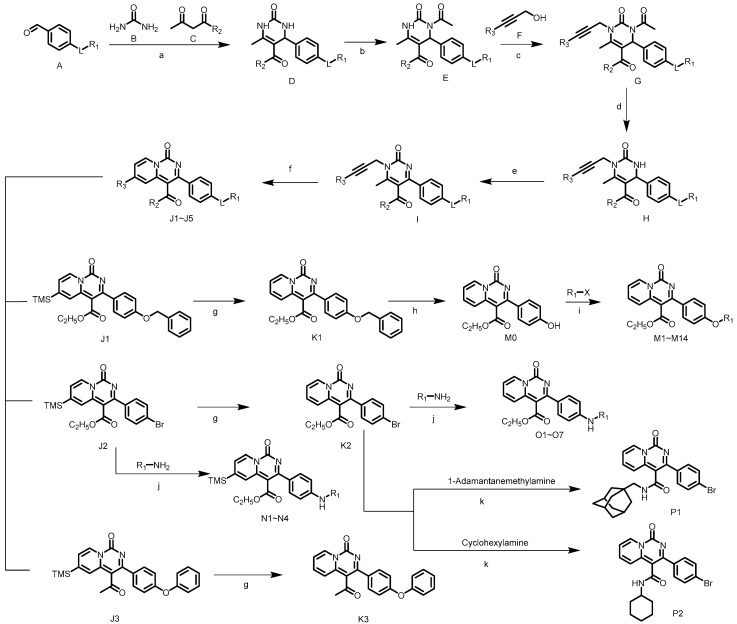
Synthetic Route of the Target Compound, Reagents and conditions: (a) Cu(NO_3_)_2_·3H_2_O, 80 °C, 1–2 h, 80–96%; (b) Ac_2_O, refluxing, 2 h, 57–84%; (c) PPh_3_, DIAD, THF, r.t., 2 h, 50–94%; (d) Piperidine, THF, 40 °C, 6 h, 84–95%; (e) BPO, EtOH, 100 °C, 8 h, 32–71%; (f) HAuCl_4_·3H_2_O, DCE, 80 °C, overnight, 18–54%; (g) TBAF (1 M in THF), THF, r.t., 1 h, 49–73%; (h) TFA, 80 °C, 1 h, 99%; (i) Cs_2_CO_3_, DMF, 80 °C, overnight, 29–50%; (j) Pd_2_(dba)_3_, Xantphos, Cs_2_CO_3_, toluene, 100 °C, overnight, 17–62%; (k) Cs_2_CO_3_, THF, 80 °C, overnight, 26–32%.

The synthesis of PPO derivatives is illustrated in Scheme 1. All target compounds were prepared via a multistep sequential synthetic route, and the detailed procedures are described below. The pyrimidine core was first constructed via the Biginelli reaction. Ethyl acetoacetate, urea, and the corresponding aldehyde (1:1:1 molar ratio) were heated at 80 °C using Cu(NO_3_)_2_·3H_2_O as the catalyst to afford intermediate **D** [34]. Acetylation of **D** with acetic anhydride at 140 °C gave intermediate **E** [35]. Intermediate **E** was then converted to intermediate **G** via a Mitsunobu reaction with alkynyl alcohols [35]. Deacetylation of **G** in the presence of piperidine furnished intermediate **H** [35]. Aromatization of the pyrimidinone ring was achieved with benzoyl peroxide (BPO) as the oxidant. Finally, annulation between the pyridine and pyrimidinone rings was accomplished with HAuCl_4_·3H_2_O as a Lewis acid catalyst [35]. After purification by column chromatography, compounds **J1**–**J5** were efficiently obtained. Compounds **K1**–**K3** were prepared by desilylation of **J1**–**J3** with 1.0 equiv of tetra-n-butylammonium fluoride (TBAF, 1 mol/L in THF). Debenzylation of **K1** with trifluoroacetic acid (TFA) at 80 °C provided the key intermediate **M0**. Nucleophilic substitution of **M0** with commercially available aryl or alkyl halides under basic conditions afforded compounds **M1**–**M14**. Compounds **N1**–**N4** and **O1**–**O7** were synthesized from **J2**/**K2** and commercially available substituted anilines or benzylamines via the Buchwald–Hartwig C–N cross-coupling reaction. Compounds **P1** and **P2** were obtained by aminolysis of the ethyl ester group.

#### 2.1.2. NMR Characterization

Compound **M0** was taken as a representative example to analyze the ^1^H NMR and ^13^C NMR spectral data. (1) ^1^H NMR analysis: due to the influence of the conjugated system of the PPO core and the electronic effects of the substituents, the aromatic hydrogens on the core exhibit a characteristic distribution of chemical shifts: The proton at the 8-position appears as a doublet at 9.12 ppm with J = 7.0 Hz. The proton at the 7-position appears as a triplet of doublets at 7.55 ppm with J = 6.5 Hz and J = 2.2 Hz. The two protons at the 5- and 6-positions of the parent nucleus overlap due to their similar chemical shifts and appear as a multiplet from 8.08 to 8.14 ppm. In the 4-substituted ethyl ester group, the methylene group exhibits a quartet at 4.09 ppm, and the methyl group exhibits a triplet at 0.96 ppm with the same J = 7.1 Hz. In the 3-substituted para-hydroxyphenyl group, the two sets of aromatic hydrogens on the benzene ring appear as doublets at 6.86 ppm and 7.45 ppm, respectively. The active hydrogen of the phenolic hydroxyl group appears as a singlet at 9.96 ppm. (2) ^13^C NMR analysis: Sp^2^-hybridized carbon atoms bonded to N and O heteroatoms on the parent nucleus are affected by the de-shielding effect of the heteroatoms. Their chemical shifts are distributed in the range of 146.99–166.75 ppm. The signals of the remaining aromatic carbon atoms on the parent nucleus and the benzene ring are distributed in the range of 102.45–139.57 ppm. The signals corresponding to the methyl and methylene carbon atoms appear at 61.16 ppm and 13.44 ppm, respectively.

### 2.2. Analysis of Anti-PEDV Activity and Structure–Activity Relationship

#### 2.2.1. Anti-PEDV Activity

To investigate the effects of R_1_, R_2_, and R_3_ substituents of the PPO skeleton on the anti-PEDV activity, the present study designed and synthesized 36 target compounds and assessed their anti-PEDV activity at a concentration of 10 μM (Table 1).

#### 2.2.2. Analysis of Structure–Activity Relationship (SAR)

**Figure 3:**
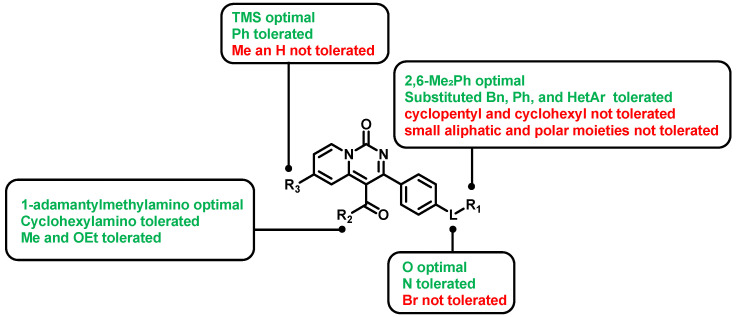
Summary of the SAR for the PPO derivatives.

A systematic structure–activity relationship (SAR) analysis was performed on this series of derivatives, and the results are shown in Figure 3.

We first investigated the effect of substituents at the R_1_ position. When the linker L is O, replacement of the phenyl group at R_1_ with small aliphatic or polar moieties (**M0**, **M1**, **M2**, **M3**) led to a marked loss of antiviral activity. Although the introduction of five-membered aromatic heterocycles (**M4**, **M5**) or alicyclic groups (**M13**, **M14**) partially restored activity, it remained weaker than that observed with the bulky biaryl group (**M6**). Moreover, when R_1_ is a benzyl group (**K1**), introducing substituents with different electronic properties onto the benzyl aromatic ring (**M7**–**M11**) or replacing the benzene ring with a pyridine ring (**M12**) is beneficial to improving antiviral activity. When L is NH and R_3_ is H, the electronegativity of substituents on the phenyl ring of R_1_ significantly influences activity: the introduction of electron-withdrawing groups (**O2** > **O1**) markedly improved the inhibition rate. Replacing R_1_ with an unsubstituted benzyl group (**O1** > **O6**) was detrimental to activity, whereas antiviral potency was substantially restored when an electron-withdrawing group was attached to the benzyl moiety (**O7** > **O1** > **O6**). When L is NH and R_3_ is TMS, and both the positional distribution and electronic properties of substituents on the phenyl ring of R_1_ also markedly modulate antiviral activity: For 2,6-disubstituted phenyl analogs, the introduction of either electron-donating groups (**N1**) or halogen substituents (**N2**) favorably enhances potency. In contrast, in 2,4-disubstituted phenyl derivatives, improved activity is observed with electron-withdrawing groups and halogens (**N4**), whereas electron-donating substitution (**N3**) exerts a detrimental effect. By comparing compounds that differ only in the linking atom L, we found that antiviral activity is generally higher when L is O rather than NH (**K1** > **O6**; **M8** > **O7**). Furthermore, when L is Br, the anti-PEDV activity decreases significantly (**J2** and **K2**). These results indicate that the introduction of a large, aromatic, hydrophobic group at the R_1_ position favors enhanced antiviral activity and that the electronic and steric properties of aromatic substituents strongly modulate potency.

Next, the influence of the steric volume of the R_3_ substituent (TMS, Ph, Me, H) on activity was evaluated. The results demonstrated that compounds with TMS or phenyl at R_3_ exhibited significantly higher activity than analogs substituted with hydrogen or methyl (**J1** > **K1**; **J2** > **K2**; **J3** > **K3**; **J4** > **J5**; **N2** > **O4**; **N3** > **O3**; **N4** > **O2**). This indicates that the introduction of a bulky hydrophobic group at the R_3_ position is also critical for enhancing anti-PEDV activity.

Finally, the effect of substituents at R_2_ was explored. When the ethoxy group at R_2_ was replaced by methyl (**J5**), the inhibition rate remained at 99.99% at the concentration of 10 μM, suggesting that the ester moiety at this position is not essential for activity. When the ethoxy group at R_2_ of **K2** was replaced by adamantylmethylamine (**P1**) and cyclohexylamine (**P2**), the antiviral activity of the compound was significantly enhanced (**P1** > **P2** > **K2**). These findings further confirm that the installation of a bulky hydrophobic group at the R_2_ position is crucial for improving anti-PEDV activity, offering valuable clues for further structure optimization.

### 2.3. Antiviral Activity, Cell Cytotoxicity and Metabolic Stability Assays of the Preferred Compounds

#### 2.3.1. Antiviral Activity and Cell Cytotoxicity of the Preferred Compounds

To further quantify the anti-PEDV activity of hit compounds identified in the primary screening, we determined the half-maximal effective concentration (EC_50_) to assess antiviral potency and the half-maximal cytotoxic concentration (CC_50_) to evaluate cytotoxicity against normal host cells. The selective index (SI), calculated as the ratio of CC_50_ to EC_50_, was used to evaluate the therapeutic safety window of each compound. All data are presented as mean ± standard error (SE) from three independent experiments (*n* = 3), with results summarized in Table 2.

Among the tested compounds, compounds **N1** and **N2** exhibited the most potent anti-PEDV activity, with EC_50_ values of 0.32 μM and 0.37 μM, respectively. However, the aforementioned compounds demonstrated non-favorable safety profiles, with CC_50_ values of 14.01 μM and 15.87 μM yielding SI values of 43.78 and 42.89, respectively. Furthermore, although compound **J4** did not exhibit the most potent anti-PEDV activity (EC_50_ = 1.77 μM), it showed markedly lower cytotoxicity than the other tested compounds (CC_50_ = 60.47 μM), with a SI value of 34.16. Further SAR analysis revealed that the introduction of the TMS group at the R_3_ position of the PPO skeleton enhanced antiviral activity but simultaneously increased cytotoxicity. Subsequent structural optimization will focus on the R_3_ substituent to improve potency and reduce cellular toxicity.

#### 2.3.2. Metabolic Stability Assays of the Preferred Compounds

Metabolic stability of compounds **J1**, **J3**, **J4**, **N1,** and **N2** was evaluated in human and mouse liver microsomes based on their antiviral activity (Table 3). Compounds **J3** and **J4** displayed moderate metabolic stability in mouse liver microsomes. In contrast, compounds **J1**, **N1,** and **N2** exhibited poor metabolic stability in both human and mouse liver microsomes.

## 3. Materials and Methods

### 3.1. General Experimental Information

All reagents and solvents were used as received from commercial suppliers without further purification. Unless otherwise stated, all reagents were of at least chemically pure (CP) grade, and all solvents were of analytical reagent (AR) grade; solvents dedicated to high-performance liquid chromatography (HPLC) were of chromatographic grade. Reaction progress was monitored by analytical thin-layer chromatography (TLC) on pre-coated silica gel plates (Yantai Jiangyou Silica Gel Development Co., Ltd., Yantai, China), with visualization under UV irradiation at 254 nm. Flash column chromatography was performed using 200–300 mesh silica gel (Yantai Jiangyou Silica Gel Co., Ltd.), and preparative TLC (PTLC) was conducted using 1 mm thick pre-coated silica gel plates (Yantai Jiangyou Silica Gel Co., Ltd.). ^1^H and ^13^C nuclear magnetic resonance (NMR) spectra of key intermediates and final target compounds were acquired on a Bruker 600 MHz NMR spectrometer (Bruker Corporation, Billerica, MA, USA). Residual non-deuterated solvent peaks were used as internal references: CDCl_3_ (^1^H NMR: δ 7.26 ppm, ^13^C NMR: δ 77.06 ppm), DMSO-d_6_ (^1^H NMR: δ 2.50 ppm, ^13^C NMR: δ 39.6 ppm), and CD_3_OD (^1^H NMR: δ 3.31 ppm, ^13^C NMR: δ 49.03 ppm). Chemical shifts (δ) are reported in parts per million (ppm), and coupling constants (*J*) are expressed in hertz (Hz). High-resolution electrospray ionization mass spectrometry (ESI-HRMS) characterization of selected key target compounds was performed on a Sciex ZenoTOF^TM^ 7600 system (AB Sciex Pte. Ltd., Singapore). All compounds are >95% pure, as determined by HPLC.

### 3.2. Synthesis of Compounds

#### 3.2.1. Synthesis of Ethyl 3-(4-(Benzyloxy)phenyl)-1-oxo-6-(trimethylsilyl)-1H-pyrido[1,2-*c*]pyrimidine-4-carboxylate (**J1**)

- Synthesis of ethyl 4-(4-(benzyloxy)phenyl)-6-methyl-2-oxo-1,2,3,4-tetrahydropyrimidine-5-carboxylate (**J1-1**).

4-(Benzyloxy)benzaldehyde (30 g, 1.0 equiv), ethyl acetoacetate (20 mL, 1.1 equiv), urea (9.3 g, 1.1 equiv), and Cu(NO_3_)_2_·3H_2_O (3.4 g, 0.1 equiv) were added to a 100 mL round-bottom flask. The mixture was stirred at 80 °C until a solid formed, at which point the reaction was terminated. For workup, the solid was washed with water, filtered, and the filter cake was washed with MTBE. The solid was dried under vacuum to afford 49.2 g of compound **J1-1** as a pale green powder in a 95% yield. ^1^H NMR (600 MHz, DMSO-*d*_6_) δ 9.16 (d, *J* = 2.4 Hz, 1H), 7.67 (dd, *J* = 3.5, 2.1 Hz, 1H), 7.45–7.41 (m, 2H), 7.38 (dd, *J* = 8.4, 6.8 Hz, 2H), 7.34–7.30 (m, 1H), 7.18–7.12 (m, 2H), 6.98–6.93 (m, 2H), 5.10 (d, *J* = 3.4 Hz, 1H), 5.07 (s, 2H), 3.98 (q, *J* = 7.1 Hz, 2H), 2.25 (s, 3H), 1.09 (t, *J* = 7.1 Hz, 3H). ESI-MS *m*/*z* 367.40 [M + H]^+^.

- Synthesis of ethyl 3-acetyl-4-(4-(benzyloxy)phenyl)-6-methyl-2-oxo-1,2,3,4-tetrahydropyrimidine-5-carboxylate (**J1-2**).

**J1-1** (40 g, 1.0 equiv) and acetic anhydride (100 mL) were added to a 250 mL round-bottom flask. The mixture was stirred at 140 °C under reflux for 2 h until TLC indicated the reaction was complete. For workup, the mixture was cooled to room temperature, and saturated aqueous sodium bicarbonate and ethyl acetate (EA) were added. The organic layer was washed with saturated aqueous sodium chloride, dried over anhydrous sodium sulfate, filtered, and concentrated. The residue was triturated with MTBE to afford 37.5 g of compound **J1-2** as a pale yellow powder in an 84% yield. ^1^H NMR (600 MHz, Chloroform-*d*) δ 8.76–8.61 (m, 1H), 7.43–7.35 (m, 4H), 7.34–7.31 (m, 1H), 7.28 (dd, *J* = 9.7, 2.9 Hz, 2H), 6.92–6.87 (m, 2H), 6.60 (d, *J* = 4.5 Hz, 1H), 5.03 (d, *J* = 2.2 Hz, 2H), 4.17 (qd, *J* = 7.0, 2.7 Hz, 2H), 2.59–2.54 (m, 3H), 2.41 (d, *J* = 3.0 Hz, 3H), 1.25 (td, *J* = 7.2, 2.1 Hz, 3H). ESI-MS *m*/*z* 409.41 [M + H]^+^.

- Synthesis of ethyl 3-acetyl-4-(4-(benzyloxy)phenyl)-6-methyl-2-oxo-1-(3-(trimethylsilyl)prop-2-yn-1-yl)-1,2,3,4-tetrahydropyrimidine-5-carboxylate (**J1-3**).

**J1-2** (32 g, 1.0 equiv), PPh_3_ (22.6 g, 1.1 equiv), and THF (150 mL) were added to a 250 mL round-bottom flask. Then DIAD (17.5 g, 1.1 equiv) and 3-(trimethylsilyl)prop-2-yn-1-ol (12.8 mL, 1.1 equiv) were added, and the mixture was stirred at room temperature for 8 h until TLC indicated the reaction was complete. For workup, water and ethyl acetate (EA) were added, and the mixture was separated into layers. The organic phase was washed with saturated aqueous NaCl, dried over anhydrous Na_2_SO_4_, filtered, and concentrated. Purification by silica gel column chromatography (dry packing, eluent: PE/EA = 30:1 to 20:1) afforded 38 g of compound **J1-3** as a colorless oil in a 94% yield. ^1^H NMR (600 MHz, Chloroform-*d*) δ 7.41–7.35 (m, 4H), 7.33–7.29 (m, 1H), 7.24–7.21 (m, 2H), 6.88–6.83 (m, 2H), 6.60 (s, 1H), 5.01 (s, 2H), 4.77 (d, *J* = 18.2 Hz, 1H), 4.33 (d, *J* = 18.2 Hz, 1H), 4.18 (qd, *J* = 7.1, 3.8 Hz, 2H), 2.70 (s, 3H), 2.51 (s, 3H), 1.24 (t, *J* = 7.1 Hz, 3H), 0.17 (d, *J* = 1.4 Hz, 9H). ESI-MS *m*/*z* 520.04 [M + H]^+^.

- Synthesis of ethyl 4-(4-(benzyloxy)phenyl)-6-methyl-2-oxo-1-(3-(trimethylsilyl)prop-2-yn-1-yl)-1,2,3,4-tetrahydropyrimidine-5-carboxylate (**J1-4**).

**J1-3** (24 g, 1.0 equiv), piperidine (19.7 g, 5.0 equiv), and THF (100 mL) were added to a 250 mL round-bottom flask. The mixture was stirred at 45 °C for 6 h until TLC indicated the reaction was complete. For workup, water and ethyl acetate (EA) were added, and the layers were separated. The organic phase was washed with saturated aqueous NaCl, dried over anhydrous Na_2_SO_4_, filtered, and concentrated. The residue was triturated with MTBE/hexane (1:3) to afford 18.6 g of compound **J1-4** as a white powder in an 84% yield. ^1^H NMR (600 MHz, Chloroform-*d*) δ 7.43–7.36 (m, 4H), 7.34–7.30 (m, 1H), 7.22–7.18 (m, 2H), 6.91–6.86 (m, 2H), 5.51–5.43 (m, 1H), 5.29 (d, *J* = 2.9 Hz, 1H), 5.03 (s, 2H), 4.82 (d, *J* = 18.2 Hz, 1H), 4.40 (d, *J* = 18.1 Hz, 1H), 4.09 (qd, *J* = 7.1, 2.8 Hz, 2H), 2.66 (d, *J* = 0.8 Hz, 3H), 1.18 (t, *J* = 7.1 Hz, 3H), 0.17 (s, 9H). ESI-MS *m*/*z* 477.44 [M + H]^+^.

- Synthesis of ethyl 4-(4-(benzyloxy)phenyl)-6-methyl-2-oxo-1-(3-(trimethylsilyl)prop-2-yn-1-yl)-1,2-dihydropyrimidine-5-carboxylate (**J1-5**).

**J1-4** (18 g, 1.0 equiv), BPO (32 g, 3.5 equiv), and ethanol (80 mL) were added to a 250 mL round-bottom flask. The mixture was stirred at 100 °C for 2 h until TLC indicated the reaction was complete. For workup, the mixture was cooled to room temperature, and saturated aqueous sodium sulfite solution and ethyl acetate (EA) were added. The organic layer was washed with saturated aqueous NaCl, dried over anhydrous Na_2_SO_4_, filtered, and concentrated. Purification by silica gel column chromatography (wet packing, eluent: PE/EA = 10:1 to 3:1) afforded 9.8 g of compound **J1-5** as a brown oil in a 55% yield. ^1^H NMR (600 MHz, Chloroform-d) δ 7.63–7.59 (m, 2H), 7.41 (d, J = 7.2 Hz, 2H), 7.37 (t, J = 7.5 Hz, 2H), 7.32 (t, J = 7.2 Hz, 1H), 6.98 (d, J = 8.7 Hz, 2H), 5.10 (s, 2H), 4.94 (s, 2H), 4.07 (q, J = 7.1 Hz, 2H), 2.67 (s, 3H), 0.97 (t, J = 7.1 Hz, 3H), 0.16 (d, J = 1.0 Hz, 9H). ESI-MS *m*/*z* 475.44 [M + H]^+^.

- Synthesis of ethyl 3-(4-(benzyloxy)phenyl)-1-oxo-6-(trimethylsilyl)-1H-pyrido[1,2-*c*]pyrimidine-4-carboxylate (**J1**).

**J1-5** (6.1 g, 1.0 equiv), hydrogen tetrachloroaurate (III) trihydrate (1.4 g, 0.3 equiv), and 1,2-dichloroethane (DCE, 30 mL) were placed in a 100 mL round-bottom flask. The mixture was stirred at 80 °C for 8 h. Then, dichloromethane (DCM) and saturated aqueous sodium sulfite solution were added, and the layers were separated. The organic phase was washed with saturated aqueous NaCl, dried over anhydrous Na_2_SO_4_, filtered, and concentrated. Purification by silica gel column chromatography (dry packing, eluent: PE/EA = 20:1 to 3:1) afforded 2.6 g of compound **J1** as a pale yellow powder in a 43% yield. ^1^H NMR (600 MHz, Chloroform-*d*) δ 9.18 (dd, *J* = 6.8, 0.9 Hz, 1H), 8.37 (t, *J* = 1.1 Hz, 1H), 7.68–7.64 (m, 2H), 7.44 (d, *J* = 7.0 Hz, 2H), 7.38 (tt, *J* = 6.1, 1.0 Hz, 3H), 7.34–7.31 (m, 1H), 7.04–7.00 (m, 2H), 5.13 (s, 2H), 4.10 (q, *J* = 7.2 Hz, 2H), 0.96 (t, *J* = 7.1 Hz, 3H), 0.38 (s, 9H). ^13^C NMR (151 MHz, Chloroform-*d*) δ 167.37, 166.52, 160.62, 155.93, 150.28, 145.93, 136.69, 132.15, 130.45, 128.80, 128.75, 128.20, 127.57, 127.21, 121.77, 114.71, 103.27, 70.16, 61.69, 13.72, −1.91. HRMS (ESI): *m*/*z* calced for C_27_H_29_N_2_O_4_Si [M + H]^+^: 473.18911, found: 473.18845. Compounds **J2**–**J5** were prepared according to the synthetic procedure for compound **J1**.

#### 3.2.2. Synthesis of Ethyl 3-(4-Bromophenyl)-1-oxo-6-(trimethylsilyl)-1H-pyrido[1,2-*c*]pyrimidine-4-carboxylate (**J2**)

- Synthesis of ethyl 4-(4-bromophenyl)-6-methyl-2-oxo-1,2,3,4-tetrahydropyrimidine-5-carboxylate (**J2-1**).

The title compound **J2-1** was prepared via a procedure similar to that for **J1-1**, yielding 33.3 g in a 96% yield. ^1^H NMR (600 MHz, DMSO-*d*6) δ 9.24 (s, 1H), 7.76 (s, 1H), 7.57–7.47 (m, 2H), 7.23–7.13 (m, 2H), 5.13 (s, 1H), 4.05–3.92 (m, 2H), 2.25 (s, 3H), 1.09 (s, 3H). ESI-MS *m*/*z* 339.34 [M + H]^+^.

- Synthesis of ethyl 3-acetyl-4-(4-bromophenyl)-6-methyl-2-oxo-1,2,3,4-tetrahydropyrimidine-5-carboxylate (**J2-2**).

The title compound **J2-2** was prepared according to a procedure similar to that for **J1-2**, affording 19.256 g of a white solid in a 57.1% yield. ^1^H NMR (600 MHz, DMSO-*d*6) δ 10.21 (s, 1H), 7.55–7.52 (m, 2H), 7.16–7.12 (m, 2H), 6.40 (s, 1H), 4.11 (qq, *J* = 7.1, 3.8 Hz, 2H), 2.43 (s, 3H), 2.31 (s, 3H), 1.17 (t, *J* = 7.1 Hz, 3H).

- Synthesis of ethyl 3-acetyl-4-(4-bromophenyl)-6-methyl-2-oxo-1-(3-(trimethylsilyl)prop-2-yn-1-yl)-1,2,3,4-tetrahydropyrimidine-5-carboxylate (**J2-3**).

Compound **J2-3** was prepared via a procedure similar to that for **J1-3**, affording 19.3 g of a white solid in a 90% yield. ^1^H NMR (600 MHz, DMSO-*d*6) δ 7.51–7.48 (m, 2H), 7.14–7.10 (m, 2H), 6.45 (s, 1H), 4.78 (d, *J* = 18.6 Hz, 1H), 4.41 (s, 1H), 4.16 (qd, *J* = 7.1, 2.0 Hz, 2H), 2.61 (s, 3H), 2.41 (s, 3H), 1.19 (t, *J* = 7.1Hz,4H), 0.11 (s,9H).

- Synthesis of ethyl 4-(4-bromophenyl)-6-methyl-2-oxo-1-(3-(trimethylsilyl)prop-2-yn-1-yl)-1,2,3,4-tetrahydropyrimidine-5-carboxylate (**J2-4**).

Compound **J2-4** was prepared by a procedure similar to that for **J1-4**, affording 13.4 g of a white solid in a 86% yield. ^1^H NMR (600 MHz, DMSO-*d*6) δ 8.14 (d, *J* = 3.8 Hz, 1H), 7.51–7.48 (m, 2H), 7.19–7.16 (m, 2H), 5.13 (d, *J* = 3.7 Hz, 1H), 4.66 (d, *J* = 18.4 Hz, 1H), 4.44 (d, *J* = 18.4 Hz, 1H), 4.03 (qd, *J* = 7.1, 4.0 Hz, 2H), 2.59 (s, 3H), 1.12 (t, *J* = 7.1 Hz, 3H), 0.14 (s, 9H).

- Synthesis of ethyl 4-(4-bromophenyl)-6-methyl-2-oxo-1-(3-(trimethylsilyl)prop-2-yn-1-yl)-1,2-dihydropyrimidine-5-carboxylate (**J2-5**).

Compound **J2-5** was prepared via a procedure similar to that for **J1-5**, affording 4.15 g of a white solid in a 34% yield. ^1^H NMR (600 MHz, Methanol-*d*4) δ 7.67–7.64 (m, 2H), 7.50–7.47 (m, 2H), 5.03 (s, 2H),4.08 (q, *J* = 7.2Hz,2H),0.97(t, *J* = 7.1Hz,3H),0.17(s,9H). ESI-MS *m*/*z* 447.45 [M + H]^+^.

- Synthesis of ethyl 3-(4-bromophenyl)-1-oxo-6-(trimethylsilyl)-1H-pyrido[1,2-*c*]pyrimidine-4-carboxylate (**J2**).

Compound **J2** was prepared according to a procedure similar to that for **J1**, affording 942 mg of a white-green solid in a 46% yield. ^1^H NMR (600 MHz, DMSO-*d*6) δ 9.11 (dd, *J* = 6.8, 0.9 Hz, 1H), 8.36 (t, *J* = 1.1 Hz, 1H), 7.76 (dd, *J* = 6.9, 1.3 Hz, 1H), 7.727.69 (m, 2H), 7.49–7.46 (m, 2H), 4.08 (q, *J* = 7.1 Hz, 2H), 0.91 (t, *J* = 7.1Hz, 3H), 0.36 (s,9H). HRMS (ESI): *m*/*z* calced for C_20_H_22_BrN_2_O_3_Si [M + H]^+^: 445.0577, found: 445.0559.

#### 3.2.3. Synthesis of 4-Acetyl-3-(4-phenoxyphenyl)-6-(trimethylsilyl)-1H-pyrido[1,2-*c*]pyrimidin-1-one (**J3**)

- Synthesis of 5-acetyl-6-methyl-4-(4-phenoxyphenyl)-3,4-dihydropyrimidin-2(1H)-one (**J3-1**).

Compound **J3-1** was prepared by a procedure similar to that for **J1-1**, affording 7.8 g of a brown powder in a 96% yield. ^1^H NMR (600 MHz, DMSO-*d*_6_) δ 9.18 (d, *J* = 2.0 Hz, 1H), 7.82 (dd, *J* = 3.7, 2.0 Hz, 1H), 7.40–7.35 (m, 2H), 7.28–7.23 (m, 2H), 7.14–7.10 (m, 1H), 6.97 (td, *J* = 8.7, 3.8 Hz, 4H), 5.25 (d, *J* = 3.4 Hz, 1H), 2.29 (s, 3H), 2.12 (s, 3H). ESI-MS *m*/*z* 323.50 [M + H]^+^.

- Synthesis of 1,1’-(4-methyl-2-oxo-6-(4-phenoxyphenyl)-3,6-dihydropyrimidine-1,5(2H)-diyl)bis(ethan-1-one) (**J3-2**).

Compound **J3-2** was prepared via a procedure similar to that for **J1-2**, affording 4.4 g of a brown powder in an 80% yield. ^1^H NMR (600 MHz, Chloroform-*d*) δ 7.54 (s, 1H), 7.35–7.32 (m, 2H), 7.30–7.27 (m, 2H), 7.13–7.10 (m, 1H), 7.01–6.98 (m, 2H), 6.93–6.90 (m, 2H), 6.69 (s, 1H), 2.56 (s, 3H), 2.42 (s, 3H), 2.23 (s, 3H). ESI-MS *m*/*z* 365.45 [M + H]^+^.

- Synthesis of 1,1’-(4-methyl-2-oxo-6-(4-phenoxyphenyl)-3-(3-(trimethylsilyl)prop-2-yn-1-yl)-3,6-dihydropyrimidine-1,5(2H)-diyl)bis(ethan-1-one) (**J3-3**).

Compound **J3-3** was prepared by a procedure similar to that for **J1-3**, affording 1.3 g of a pale yellow oil in a 50% yield. ^1^H NMR (600 MHz, Chloroform-*d*) δ 7.34 (dd, *J* = 8.5, 7.3 Hz, 2H), 7.30–7.27 (m, 2H), 7.14–7.10 (m, 1H), 7.02–6.99 (m, 2H), 6.90–6.87 (m, 2H), 6.63 (s, 1H), 4.80 (d, *J* = 18.1 Hz, 1H), 4.31 (d, *J* = 18.2 Hz, 1H), 2.67 (d, *J* = 0.8 Hz, 3H), 2.52 (s, 3H), 2.24 (s, 3H), 0.13 (s, 9H). ESI-MS *m*/*z* 474.55 [M + H]^+^.

- Synthesis of 5-acetyl-6-methyl-4-(4-phenoxyphenyl)-1-(3-(trimethylsilyl)prop-2-yn-1-yl)-3,4-dihydropyrimidin-2(1H)-one (**J3-4**).

Compound **J3-4** was prepared by a procedure similar to that for **J1-4**, affording 986 mg of a white powder in an 85% yield. ^1^H NMR (600 MHz, Chloroform-*d*) δ 7.36–7.32 (m, 2H), 7.24–7.22 (m, 2H), 7.14–7.11 (m, 1H), 7.02–6.99 (m, 2H), 6.94–6.91 (m, 2H), 5.55 (d, *J* = 11.6 Hz, 1H), 5.28–5.26 (m, 1H), 4.83 (d, *J* = 18.3 Hz, 1H), 4.38 (d, *J* = 18.2 Hz, 1H), 2.62 (s, 3H), 2.16 (s, 3H), 0.14 (s, 9H). ESI-MS *m*/*z* 433.60 [M + H]^+^.

- Synthesis of 5-acetyl-6-methyl-4-(4-phenoxyphenyl)-1-(3-(trimethylsilyl)prop-2-yn-1-yl)pyrimidin-2(1H)-one (**J3-5**).

Compound **J3-5** was prepared by a procedure similar to that for **J1-5**, affording 352 mg of a pale yellow powder in a 71% yield. ^1^H NMR (600 MHz, Chloroform-*d*) δ 7.67–7.64 (m, 2H), 7.42–7.37 (m, 2H), 7.19 (tt, *J* = 7.5, 1.1 Hz, 1H), 7.08–7.05 (m, 2H), 7.04–7.01 (m, 2H), 4.97 (s, 2H), 2.62 (d, *J* = 6.6 Hz, 3H), 2.03 (s, 3H), 0.17 (s, 9H). ESI-MS *m*/*z* 431.65 [M + H]^+^.

- Synthesis of 4-acetyl-3-(4-phenoxyphenyl)-6-(trimethylsilyl)-1H-pyrido[1,2-*c*]pyrimidin-1-one (**J3**).

Compound **J3** was prepared via a procedure similar to that for **J1**, affording 107 mg of a pale yellow powder in a 43% yield. ^1^H NMR (600 MHz, Chloroform-*d*) δ 9.24–9.22 (m, 1H), 8.40 (t, *J* = 1.0 Hz, 1H), 7.72–7.69 (m, 2H), 7.43 (dd, *J* = 6.9, 1.2 Hz, 1H), 7.40–7.37 (m, 2H), 7.18 (tt, *J* = 7.4, 1.1 Hz, 1H), 7.09–7.05 (m, 4H), 2.06 (s, 3H), 0.38 (s, 9H). ^13^C NMR (151 MHz, Chloroform-*d*) δ 201.61, 165.47, 160.52, 156.73, 156.05, 150.17, 144.99, 133.33, 131.48, 130.14, 128.94, 127.08, 124.45, 122.33, 119.96, 118.34, 111.95, 33.17, −1.89. HRMS (ESI): *m*/*z* calced for C_25_H_25_N_2_O_3_Si [M + H]^+^: 429.16290, found: 429.16253.

#### 3.2.4. Synthesis of Ethyl 1-Oxo-3-(4-phenoxyphenyl)-6-phenyl-1H-pyrido[1,2-*c*]pyrimidine-4-carboxylate (**J4**)

- Synthesis of ethyl 6-methyl-2-oxo-4-(4-phenoxyphenyl)-1,2,3,4-tetrahydropyrimidine-5-carboxylate (**J4-1**).

Compound **J4-1** was prepared by a procedure similar to that for **J1-1**, affording 14.2 g of a pale green powder in an 80% yield. ESI-MS *m*/*z* 353.53 [M + H]^+^.

- Synthesis of ethyl 3-acetyl-6-methyl-2-oxo-4-(4-phenoxyphenyl)-1,2,3,4-tetrahydropyrimidine-5-carboxylate (**J4-2**).

Compound **J4-2** was prepared by a procedure similar to that for **J1-2**, affording 7.4 g of a pale yellow powder in a 66% yield. ESI-MS *m*/*z* 395.25 [M + H]^+^.

- Synthesis of ethyl 3-acetyl-6-methyl-2-oxo-4-(4-phenoxyphenyl)-1-(3-phenylprop-2-yn-1-yl)-1,2,3,4-tetrahydropyrimidine-5-carboxylate (**J4-3**).

Compound **J4-3** was prepared by a procedure similar to that for **J1-3**, affording 990 mg of a colorless oil in a 77% yield. ^1^H NMR (600 MHz, Chloroform-*d*) δ 7.37–7.35 (m, 2H), 7.33–7.28 (m, 5H), 7.25 (dd, *J* = 8.1, 6.7 Hz, 2H), 7.13–7.07 (m, 1H), 6.98–6.94 (m, 2H), 6.83–6.79 (m, 2H), 6.65 (s, 1H), 5.03 (d, *J* = 18.1 Hz, 1H), 4.54 (d, *J* = 18.1 Hz, 1H), 4.19 (q, *J* = 7.1 Hz, 2H), 2.77 (s, 3H), 2.55 (s, 3H), 1.25 (t, *J* = 7.1 Hz, 3H). ESI-MS *m*/*z* 509.59 [M + H]^+^.

- Synthesis of ethyl 6-methyl-2-oxo-4-(4-phenoxyphenyl)-1-(3-phenylprop-2-yn-1-yl)-1,2,3,4-tetrahydropyrimidine-5-carboxylate (**J4-4**).

Compound **J4-4** was prepared by a procedure similar to that for **J1-4**, affording 861 mg of a white powder in a 95% yield. ^1^H NMR (600 MHz, Chloroform-*d*) δ 7.42–7.37 (m, 2H), 7.35–7.29 (m, 3H), 7.31–7.22 (m, 6H), 7.10 (tt, *J* = 7.4, 1.1 Hz, 1H), 7.00–6.94 (m, 2H), 6.89–6.84 (m, 2H), 5.78 (d, *J* = 3.3 Hz, 1H), 5.35 (d, *J* = 3.0 Hz, 1H), 5.00 (d, *J* = 18.1 Hz, 1H), 4.63 (d, *J* = 18.1 Hz, 1H), 4.11 (q, *J* = 7.1 Hz, 2H), 2.73 (d, *J* = 0.8 Hz, 3H), 1.19 (t, *J* = 7.1 Hz, 3H). ESI-MS *m*/*z* 467.41 [M + H]^+^.

- Synthesis of ethyl 6-methyl-2-oxo-4-(4-phenoxyphenyl)-1-(3-phenylprop-2-yn-1-yl)-1,2-dihydropyrimidine-5-carboxylate (**J4-5**).

Compound **J4-5** was prepared by a procedure similar to that for **J1-5**, affording 220 mg of a pale yellow powder in a 44% yield. ^1^H NMR (600 MHz, Chloroform-*d*) δ 7.66–7.62 (m, 2H), 7.46–7.42 (m, 2H), 7.39–7.36 (m, 2H), 7.32 (ddd, *J* = 14.3, 7.9, 6.2 Hz, 3H), 7.17 (t, *J* = 7.4 Hz, 1H), 7.06–7.03 (m, 2H), 7.02–7.00 (m, 2H), 5.19 (s, 2H), 4.10 (q, *J* = 7.1 Hz, 2H), 2.78 (s, 3H), 1.03 (t, *J* = 7.1 Hz, 3H). ESI-MS *m*/*z* 465.58 [M + H]^+^.

- Synthesis of ethyl 1-oxo-3-(4-phenoxyphenyl)-6-phenyl-1H-pyrido[1,2-*c*]pyrimidine-4-carboxylate (**J4**).

Compound **J4** was prepared via a procedure similar to that for **J1**, affording 107 mg of a pale yellow powder in a 54% yield. ^1^H NMR (600 MHz, Chloroform-*d*) δ 9.33 (d, *J* = 7.1 Hz, 1H), 8.56 (d, *J* = 1.8 Hz, 1H), 7.81–7.75 (m, 2H), 7.71–7.66 (m, 2H), 7.62 (dd, *J* = 7.3, 2.0 Hz, 1H), 7.57 (dd, *J* = 5.0, 1.9 Hz, 3H), 7.40–7.35 (m, 2H), 7.16 (t, *J* = 7.4 Hz, 1H), 7.09–7.02 (m, 4H), 4.12 (q, *J* = 7.2 Hz, 2H), 0.99 (t, *J* = 7.1 Hz, 3H). ^13^C NMR (151 MHz, Chloroform-*d*) δ 167.28, 166.77, 159.50, 156.58, 150.81, 150.07, 148.01, 135.85, 134.35, 131.72, 131.17, 130.52, 130.05, 129.74, 127.64, 124.07, 119.58, 118.71, 118.14, 117.46, 103.58, 61.70, 13.68. HRMS (ESI): *m*/*z* calced for C_29_H_23_N_2_O_4_ [M + H]^+^: 463.16523, found: 463.16437.

#### 3.2.5. Synthesis of Ethyl 6-Methyl-1-oxo-3-(4-phenoxyphenyl)-1H-pyrido[1,2-*c*]pyrimidine-4-carboxylate (**J5**)

- Synthesis of ethyl 3-acetyl-1-(but-2-yn-1-yl)-6-methyl-2-oxo-4-(4-phenoxyphenyl)-1,2,3,4-tetrahydropyrimidine-5-carboxylate (**J5-1**).

Compound **J5-1** was prepared via a procedure similar to that for **J1-3**, affording 815 mg of a colorless oil in a 72% yield. ^1^H NMR (600 MHz, Chloroform-*d*) δ 7.32 (dd, *J* = 8.6, 7.3 Hz, 2H), 7.26 (d, *J* = 8.7 Hz, 2H), 7.12–7.07 (m, 1H), 7.01–6.96 (m, 2H), 6.90–6.85 (m, 2H), 6.62 (s, 1H), 4.60 (dd, *J* = 17.8, 2.5 Hz, 1H), 4.35–4.28 (m, 1H), 4.19 (q, *J* = 7.1 Hz, 2H), 2.69 (s, 3H), 2.53 (s, 3H), 1.79 (t, *J* = 2.4 Hz, 3H), 1.25 (t, *J* = 7.1 Hz, 3H). ESI-MS *m*/*z* 447.52 [M + H]^+^.

- Synthesis of ethyl 1-(but-2-yn-1-yl)-6-methyl-2-oxo-4-(4-phenoxyphenyl)-1,2,3,4-tetrahydropyrimidine-5-carboxylate (**J5-2**).

Compound **J5-2** was prepared by a procedure similar to that for **J1-4**, affording 631 mg of a white powder in an 85% yield. ^1^H NMR (600 MHz, Chloroform-*d*) δ 7.35–7.31 (m, 2H), 7.26–7.23 (m, 2H), 7.10 (tt, *J* = 7.4, 1.1 Hz, 1H), 7.00–6.97 (m, 2H), 6.93–6.90 (m, 2H), 5.49 (d, *J* = 3.1 Hz, 1H), 5.33 (d, *J* = 2.8 Hz, 1H), 4.63–4.57 (m, 1H), 4.45–4.40 (m, 1H), 4.11 (q, *J* = 7.1 Hz, 2H), 2.66 (d, *J* = 0.8 Hz, 3H), 1.81 (t, *J* = 2.3 Hz, 3H), 1.19 (t, *J* = 7.1 Hz, 3H). ESI-MS *m*/*z* 405.57 [M + H]^+^.

- Synthesis of ethyl 1-(but-2-yn-1-yl)-6-methyl-2-oxo-4-(4-phenoxyphenyl)-1,2-dihydropyrimidine-5-carboxylate (**J5-3**).

Compound **J5-3** was prepared via a procedure similar to that for **J1-5**, affording 160 mg of a pale yellow powder in a 32% yield. ^1^H NMR (600 MHz, Chloroform-*d*) δ 7.65–7.60 (m, 2H), 7.39–7.35 (m, 2H), 7.18–7.15 (m, 1H), 7.05–7.02 (m, 2H), 7.02–6.99 (m, 2H), 4.88 (s, 2H), 4.13–4.08 (m, 2H), 2.70 (s, 3H), 1.82 (d, *J* = 2.0 Hz, 3H), 1.03 (t, *J* = 7.1 Hz, 3H). ESI-MS *m*/*z* 403.68 [M + H]^+^.

- Synthesis of ethyl 6-methyl-1-oxo-3-(4-phenoxyphenyl)-1H-pyrido[1,2-*c*]pyrimidine-4-carboxylate (**J5**).

Compound **J5** was prepared via a procedure similar to that for **J1**, affording 28 mg of a pale yellow powder in an 18% yield. ^1^H NMR (600 MHz, Chloroform-*d*) δ 9.20 (d, *J* = 7.1 Hz, 1H), 8.11 (s, 1H), 7.68–7.63 (m, 2H), 7.37 (t, *J* = 7.8 Hz, 2H), 7.21 (dd, *J* = 7.2, 1.8 Hz, 1H), 7.15 (t, *J* = 7.4 Hz, 1H), 7.07–7.03 (m, 4H), 4.10 (q, *J* = 7.2 Hz, 2H), 2.57 (s, 3H), 0.98 (t, *J* = 7.2 Hz, 3H). ^13^C NMR (151 MHz, Chloroform-*d*) δ 159.47, 156.58, 151.88, 149.92, 147.64, 131.00, 130.47, 130.05, 124.07, 121.08, 120.97, 119.57, 118.15, 102.99, 61.70, 27.12, 22.38, 13.67. HRMS (ESI): *m*/*z* calced for C_24_H_21_N_2_O_4_ [M + H]^+^: 401.14958, found: 401.14815.

#### 3.2.6. Synthesis of Ethyl 3-(4-(Benzyloxy)phenyl)-1-oxo-1H-pyrido[1,2-*c*]pyrimidine-4-carboxylate (**K1**)

To a 25 mL round-bottom flask were added J1 (460 mg, 1.0 eq.), TBAF (978 μL, 1 M in THF, 1.0 eq.), and THF (2.5 mL). The mixture was stirred at room temperature for 1 h and was then quenched with water and extracted with DCM. The organic layer was washed with saturated brine, dried over anhydrous sodium sulfate, filtered, and concentrated in vacuo. Purification by silica gel column chromatography (dry loading, eluent: PE/EA = 20:1 → 10:1 → 5:1 → 3:1 → 1:1) afforded 193 mg of **K1** as a pale yellow powder in a 49% yield. ^1^H NMR (600 MHz, Chloroform-*d*) δ 9.28 (d, *J* = 7.0 Hz, 1H), 8.30 (d, *J* = 9.0 Hz, 1H), 7.91–7.85 (m, 1H), 7.67 (d, *J* = 8.4 Hz, 2H), 7.44 (d, *J* = 7.6 Hz, 2H), 7.39 (t, *J* = 7.5 Hz, 2H), 7.36–7.31 (m, 2H), 7.02 (d, *J* = 8.5 Hz, 2H), 5.14 (s, 2H), 4.08 (q, *J* = 7.2 Hz, 2H), 0.93 (t, *J* = 7.1 Hz, 3H). ^13^C NMR (151 MHz, Chloroform-*d*) δ 167.26, 166.54, 160.75, 150.07, 147.91, 138.36, 136.66, 131.88, 131.32, 130.53, 128.77, 128.24, 127.60, 122.47, 118.17, 114.75, 103.56, 70.19, 61.77, 13.69. HRMS (ESI): *m*/*z* calced for C_24_H_21_N_2_O_4_ [M + H]^+^: 401.14958, found: 401.14869. Compounds **K2**–**K3** were prepared by referring to the synthetic procedure of compound **K1**.

#### 3.2.7. Synthesis of Ethyl 3-(4-Bromophenyl)-1-oxo-1H-pyrido[1,2-*c*]pyrimidine-4-carboxylate (**K2**)

Compound **K2** was prepared by a procedure similar to that for **K1**, affording 503 mg of a yellow solid in a 73% yield. ^1^H NMR (600 MHz, Chloroform-*d*) δ 9.33 (dt, *J* = 7.0, 1.2 Hz, 1H), 8.39 (dt, *J* = 9.0, 1.0 Hz, 1H), 7.95 (ddd, *J* = 8.8, 6.9, 1.4 Hz, 1H), 7.60–7.52 (m, 4H), 7.42 (td, *J* = 7.0, 1.3 Hz, 1H), 4.08 (q, *J* = 7.1 Hz, 2H), 0.94 (t, *J* = 7.1 Hz, 3H). ^13^C NMR (151 MHz, Chloroform-d) δ 166.48, 166.10, 149.77, 147.95, 138.77, 138.26, 131.40, 131.37, 130.07, 124.65, 122.63, 118.72, 103.60, 61.73, 13.44.

#### 3.2.8. Synthesis of 4-Acetyl-3-(4-phenoxyphenyl)-1H-pyrido[1,2-*c*]pyrimidin-1-one (**K3**)

Compound **K3** was prepared by a procedure similar to that for **K1**, affording 40 mg of a pale yellow powder in a 65% yield. ^1^H NMR (600 MHz, Chloroform-*d*) δ 9.33 (d, *J* = 6.9 Hz, 1H), 8.31 (d, *J* = 9.0 Hz, 1H), 7.90 (ddd, *J* = 8.7, 6.8, 1.4 Hz, 1H), 7.73–7.69 (m, 2H), 7.38 (dtd, *J* = 9.3, 7.3, 1.6 Hz, 3H), 7.19 (tt, *J* = 7.4, 1.1 Hz, 1H), 7.09–7.05 (m, 4H), 2.06 (s, 3H). ^13^C NMR (151 MHz, Chloroform-*d*) δ 201.45, 165.61, 160.66, 156.00, 150.05, 146.98, 138.83, 133.12, 131.53, 131.45, 130.17, 124.51, 122.40, 120.00, 118.64, 118.34, 112.25, 33.08. HRMS (ESI): *m*/*z* calced for C_22_H_17_N_2_O_3_ [M + H]^+^: 357.12337, found: 357.12315.

#### 3.2.9. Synthesis of Ethyl 3-(4-Hydroxyphenyl)-1-oxo-1H-pyrido[1,2-*c*]pyrimidine-4-carboxylate (**M0**)

To a 25 mL round-bottom flask were added **K1** (140 mg) and TFA (2 mL). The mixture was stirred at 80 °C for 1 h, and the reaction was complete as indicated by TLC. For workup: the reaction was quenched with saturated aqueous NaHCO_3_ solution, and the mixture was extracted with ethyl acetate (EA). The organic layer was washed with brine, dried over anhydrous sodium sulfate, filtered, and concentrated in vacuo to afford 110 mg of compound **M0** as a brown powder in a 99% yield. ^1^H NMR (600 MHz, DMSO-d_6_) δ 9.96 (s, 1H), 9.12 (d, J = 7.0 Hz, 1H), 8.14–8.08 (m, 2H), 7.55 (td, J = 6.5, 2.2 Hz, 1H), 7.45 (d, J = 8.5 Hz, 2H), 6.86 (d, J = 8.5 Hz, 2H), 4.09 (q, J = 7.1 Hz, 2H), 0.96 (t, J = 7.1 Hz, 3H). ^13^C NMR (151 MHz, DMSO-d_6_) δ 166.75, 164.61, 159.45, 149.25, 146.99, 139.57, 130.88, 130.02, 129.78, 121.51, 118.99, 115.02, 102.45, 61.16, 13.44. HRMS (ESI): *m*/*z* calced for C_17_H_15_N_2_O_4_ [M + H]^+^: 311.10263, found: 311.10177.

#### 3.2.10. Synthesis of Ethyl 3-(4-(Cyclopropylmethoxy)phenyl)-1-oxo-1H-pyrido[1,2-*c*]pyrimidine-4-carboxylate (**M1**)

Compound **M0** (30 mg), (bromomethyl)cyclopropane (26 mg), Cs_2_CO_3_ (63 mg), and DMF (1.5 mL) were placed in a 10 mL round-bottom flask. The mixture was stirred at 80 °C for 12 h until the reaction was complete, as indicated by TLC. For workup, the reaction was quenched with water and extracted with ethyl acetate (EA). The organic layer was washed with saturated brine, dried over anhydrous Na_2_SO_4_, filtered, and concentrated. Purification by preparative TLC afforded 11 mg of compound M1 as a yellow powder in a 31% yield. ^1^H NMR (600 MHz, Chloroform-*d*) δ 9.29 (dt, *J* = 7.0, 1.2 Hz, 1H), 8.29 (dt, *J* = 9.0, 1.1 Hz, 1H), 7.93–7.87 (m, 1H), 7.69–7.65 (m, 2H), 7.37 (dd, *J* = 6.9, 1.3 Hz, 1H), 6.97–6.93 (m, 2H), 4.11 (q, *J* = 7.2 Hz, 2H), 3.86 (d, *J* = 6.9 Hz, 2H), 1.33–1.28 (m, 1H), 0.98 (t, *J* = 7.1 Hz, 3H), 0.69–0.65 (m, 2H), 0.37 (dt, *J* = 6.1, 4.7 Hz, 2H). ^13^C NMR (151 MHz, Chloroform-*d*) δ 167.15, 165.93, 161.30, 149.71, 147.89, 138.69, 131.44, 130.87, 130.64, 122.52, 118.35, 114.45, 103.61, 73.09, 61.86, 13.74, 10.31, 3.37. HRMS (ESI): *m*/*z* calced for C_21_H_21_N_2_O_4_ [M + H]^+^: 365.14958, found: 365.14981. Compounds **M2**–**M14** were prepared according to the synthetic procedure of compound **M1**.

#### 3.2.11. Synthesis of Ethyl 3-(4-(But-2-yn-1-yloxy)phenyl)-1-oxo-1H-pyrido[1,2-*c*]pyrimidine-4-carboxylate (**M2**)

Compound **M2** was prepared by a procedure similar to that for **M1**, affording 12 mg of a yellow powder in a 34% yield. ^1^H NMR (600 MHz, Chloroform-*d*) δ 9.29 (dt, *J* = 7.1, 1.2 Hz, 1H), 8.31 (dt, *J* = 9.0, 1.1 Hz, 1H), 7.90 (ddd, *J* = 8.5, 6.7, 1.4 Hz, 1H), 7.69–7.65 (m, 2H), 7.36 (td, *J* = 6.9, 1.3 Hz, 1H), 7.04–7.00 (m, 2H), 4.71 (q, *J* = 2.3 Hz, 2H), 4.10 (q, *J* = 7.1 Hz, 2H), 1.86 (t, *J* = 2.3 Hz, 3H), 0.95 (t, *J* = 7.1 Hz, 3H). ^13^C NMR (151 MHz, Chloroform-*d*) δ 167.12, 166.20, 159.85, 149.82, 147.92, 138.63, 131.82, 131.41, 130.45, 122.54, 118.37, 114.75, 103.63, 84.33, 61.81, 56.64, 13.64, 3.80. HRMS (ESI): *m*/*z* calced for C_21_H_19_N_2_O_4_ [M + H]^+^: 363.13393, found: 363.13549.

#### 3.2.12. Synthesis of Ethyl 3-(4-(Cyanomethoxy)phenyl)-1-oxo-1H-pyrido[1,2-*c*]pyrimidine-4-carboxylate (**M3**)

Compound **M3** was prepared by a procedure similar to that for **M1**, affording 10 mg of a yellow powder in a 30% yield. ^1^H NMR (600 MHz, Chloroform-*d*) δ 9.36 (d, *J* = 6.8 Hz, 1H), 8.38 (d, *J* = 8.9 Hz, 1H), 8.06–8.01 (m, 1H), 7.74–7.71 (m, 2H), 7.50 (t, *J* = 6.9 Hz, 1H), 7.09–7.05 (m, 2H), 4.85 (s, 2H), 4.10 (q, *J* = 7.1 Hz, 2H), 0.95 (t, *J* = 7.1 Hz, 3H). ^13^C NMR (151 MHz, Chloroform-*d*) δ 166.30, 158.46, 147.97, 140.07, 131.98, 130.93, 122.98, 119.50, 114.80, 104.12, 62.14, 53.60, 13.68. HRMS (ESI): *m*/*z* calced for C_19_H_16_N_3_O_4_+ [M + H]^+^: 350.11353, found: 350.11357.

#### 3.2.13. Ethyl 3-(4-(Oxazol-2-yloxy)phenyl)-1-oxo-1H-pyrido[1,2-*c*]pyrimidine-4-carboxylate (**M4**)

Compound **M4** was prepared by a procedure similar to that for **M1**, affording 23 mg of a yellow powder in a 45% yield. ^1^H NMR (600 MHz, Chloroform-*d*) δ 9.34 (d, *J* = 6.8 Hz, 1H), 8.40 (d, *J* = 8.9 Hz, 1H), 7.97 (ddd, *J* = 8.7, 6.8, 1.4 Hz, 1H), 7.77–7.72 (m, 2H), 7.47–7.41 (m, 3H), 7.36 (d, *J* = 1.1 Hz, 1H), 6.93 (d, *J* = 1.1 Hz, 1H), 4.09 (q, *J* = 7.1 Hz, 2H), 0.96 (t, *J* = 7.2 Hz, 3H). ^13^C NMR (151 MHz, Chloroform-*d*) δ 166.62, 165.51, 159.67, 155.06, 149.56, 148.06, 139.17, 136.09, 134.73, 131.63, 130.38, 126.90, 122.83, 118.99, 118.98, 103.96, 61.99, 29.85, 13.61. HRMS (ESI): *m*/*z* calced for C_20_H_16_N_3_O_5_ [M + H]^+^: 378.10845, found: 378.10741.

#### 3.2.14. Ethyl 1-Oxo-3-(4-(thiazol-2-yloxy)phenyl)-1H-pyrido[1,2-*c*]pyrimidine-4-carboxylate (**M5**)

Compound **M5** was prepared by a procedure similar to that for **M1**, affording 12 mg of a pale yellow powder in a 31% yield. ^1^H NMR (600 MHz, Chloroform-*d*) δ 9.36 (dd, *J* = 7.1, 1.4 Hz, 1H), 8.43–8.38 (m, 1H), 8.02–7.98 (m, 1H), 7.77–7.72 (m, 2H), 7.47 (t, *J* = 6.9 Hz, 1H), 7.40–7.36 (m, 2H), 7.29 (d, *J* = 3.8 Hz, 1H), 6.89 (d, *J* = 3.8 Hz, 1H), 4.10 (q, *J* = 7.1 Hz, 2H), 0.97 (t, *J* = 7.2 Hz, 3H). ^13^C NMR (151 MHz, Chloroform-*d*) δ 172.80, 166.51, 157.33, 149.32, 148.05, 139.44, 137.79, 135.78, 131.74, 130.59, 130.04, 122.89, 119.61, 119.15, 113.85, 104.01, 62.03, 29.47, 13.66. HRMS (ESI): *m*/*z* calced for C_20_H_16_N_3_O_4_S [M + H]^+^: 394.08560, found: 394.08599.

#### 3.2.15. Ethyl 1-Oxo-3-(4-((4-phenylthiazol-2-yl)oxy)phenyl)-1H-pyrido[1,2-*c*]pyrimidine-4-carboxylate (**M6**)

Compound **M6** was prepared by a procedure similar to that for **M1**, affording 15 mg of a yellow powder in a 33% yield. ^1^H NMR (600 MHz, Chloroform-*d*) δ 9.39 (d, *J* = 5.9 Hz, 1H), 8.44 (d, *J* = 8.7 Hz, 1H), 8.05 (t, *J* = 7.6 Hz, 1H), 7.83–7.79 (m, 2H), 7.79–7.75 (m, 2H), 7.55–7.50 (m, 1H), 7.48 (d, *J* = 8.4 Hz, 2H), 7.39 (dd, *J* = 8.4, 6.9 Hz, 2H), 7.34–7.30 (m, 1H), 7.06 (s, 1H), 4.11 (q, *J* = 7.1 Hz, 2H), 0.98 (t, *J* = 7.1 Hz, 3H). ^13^C NMR (151 MHz, Chloroform-*d*) δ 171.70, 166.22, 157.36, 149.99, 148.05, 140.15, 134.21, 132.06, 130.63, 128.82, 128.36, 126.08, 123.09, 119.84, 119.64, 107.00, 104.19, 62.18, 13.69. HRMS (ESI): *m*/*z* calced for C_26_H_20_N_3_O_4_S [M + H]^+^: 470.11690, found: 470.11711.

#### 3.2.16. Ethyl 3-(4-((3-Chlorobenzyl)oxy)phenyl)-1-oxo-1H-pyrido[1,2-*c*]pyrimidine-4-carboxylate (**M7**)

Compound **M7** was prepared by a procedure similar to that for **M1**, affording 15 mg of a yellow powder in a 36% yield. ^1^H NMR (600 MHz, Chloroform-*d*) δ 9.32 (d, *J* = 6.8 Hz, 1H), 8.33 (d, *J* = 8.9 Hz, 1H), 7.96 (t, *J* = 7.8 Hz, 1H), 7.71–7.66 (m, 2H), 7.45 (d, *J* = 2.0 Hz, 1H), 7.42 (t, *J* = 6.9 Hz, 1H), 7.34–7.29 (m, 3H), 7.04–7.00 (m, 2H), 5.12 (s, 2H), 4.09 (q, *J* = 7.1 Hz, 2H), 0.94 (t, *J* = 7.1 Hz, 3H). ^13^C NMR (151 MHz, Chloroform-*d*) δ 166.81, 160.64, 147.92, 139.30, 138.69, 134.75, 131.68, 130.77, 130.08, 128.39, 127.53, 125.50, 122.70, 118.82, 114.79, 103.77, 69.32, 61.96, 27.13, 13.68. HRMS (ESI): *m*/*z* calced for C_24_H_20_ClN_2_O_4_ [M + H]^+^: 435.11061, found: 435.11094.

#### 3.2.17. Ethyl 3-(4-((4-Chlorobenzyl)oxy)phenyl)-1-oxo-1H-pyrido[1,2-*c*]pyrimidine-4-carboxylate (**M8**)

Compound **M8** was prepared by a procedure similar to that for **M1**, affording 16 mg of a yellow powder in a 38% yield. ^1^H NMR (600 MHz, Chloroform-*d*) δ 9.32–9.29 (m, 1H), 8.31 (dt, *J* = 9.0, 1.1 Hz, 1H), 7.92 (ddd, *J* = 8.6, 6.8, 1.4 Hz, 1H), 7.69–7.66 (m, 2H), 7.41–7.34 (m, 5H), 7.03–6.99 (m, 2H), 5.10 (s, 2H), 4.09 (q, *J* = 7.2 Hz, 2H), 0.93 (t, *J* = 7.1 Hz, 3H). ^13^C NMR (151 MHz, Chloroform-*d*) δ 166.99, 165.68, 160.60, 149.56, 147.91, 138.90, 135.13, 134.08, 131.52, 131.38, 130.67, 128.96, 128.94, 122.59, 118.55, 114.76, 103.67, 69.42, 61.86, 13.68. HRMS (ESI): *m*/*z* calced for C_24_H_20_ClN_2_O_4_ [M + H]^+^: 435.11061, found: 435.11152.

#### 3.2.18. Ethyl 3-(4-((2-Chlorobenzyl)oxy)phenyl)-1-oxo-1H-pyrido[1,2-*c*]pyrimidine-4-carboxylate (**M9**)

Compound **M9** was prepared by a procedure similar to that for **M1**, affording 15 mg of a yellow powder in a 50% yield. ^1^H NMR (600 MHz, Chloroform-*d*) δ 9.32 (d, *J* = 6.8 Hz, 1H), 8.33 (d, *J* = 8.9 Hz, 1H), 7.95 (t, *J* = 7.9 Hz, 1H), 7.71–7.68 (m, 2H), 7.54 (dd, *J* = 6.7, 2.7 Hz, 1H), 7.41 (dd, *J* = 6.3, 3.1 Hz, 2H), 7.29 (td, *J* = 6.9, 6.1, 4.0 Hz, 2H), 7.05 (d, *J* = 8.5 Hz, 2H), 5.24 (s, 2H), 4.10 (q, *J* = 7.1 Hz, 2H), 0.94 (t, *J* = 7.1 Hz, 3H). ^13^C NMR (151 MHz, Chloroform-*d*) δ 166.84, 160.73, 147.92, 139.25, 134.33, 132.80, 131.66, 130.76, 129.61, 129.30, 128.91, 127.14, 122.69, 118.77, 114.80, 103.76, 67.33, 61.95, 13.69. HRMS (ESI): *m*/*z* calced for C_24_H_20_ClN_2_O_4_ [M + H]^+^: 435.11061, found: 435.11136.

#### 3.2.19. Ethyl 1-Oxo-3-(4-((2-(trifluoromethyl)benzyl)oxy)phenyl)-1H-pyrido[1,2-*c*]pyrimidine-4-carboxylate (**M10**)

Compound **M10** was prepared by a procedure similar to that for **M1**, affording 15 mg of a yellow powder in a 33% yield. ^1^H NMR (600 MHz, Chloroform-*d*) δ 9.30 (d, *J* = 6.9 Hz, 1H), 8.32 (d, *J* = 8.9 Hz, 1H), 7.92 (ddd, *J* = 8.7, 6.7, 1.3 Hz, 1H), 7.72 (t, *J* = 8.3 Hz, 2H), 7.70–7.65 (m, 2H), 7.57 (t, *J* = 7.6 Hz, 1H), 7.44 (t, *J* = 7.6 Hz, 1H), 7.37 (t, *J* = 6.9 Hz, 1H), 7.04–7.00 (m, 2H), 5.34 (s, 2H), 4.09 (q, *J* = 7.2 Hz, 2H), 0.93 (t, *J* = 7.1 Hz, 3H). ^13^C NMR (151 MHz, Chloroform-*d*) δ 166.89, 160.22, 149.56, 147.81, 138.62, 135.14, 132.17, 131.66, 131.34, 130.52, 128.66, 127.91, 126.06, 126.02, 122.45, 118.32, 114.60, 103.53, 66.21, 61.70, 13.50. HRMS (ESI): *m*/*z* calced for C_25_H_20_F_3_N_2_O_4_ [M + H]^+^: 469.13697, found: 469.13685.

#### 3.2.20. Ethyl 3-(4-((4-Methoxybenzyl)oxy)phenyl)-1-oxo-1H-pyrido[1,2-*c*]pyrimidine-4-carboxylate (**M11**)

Compound **M11** was prepared by a procedure similar to that for **M1**, affording 12 mg of a yellow powder in a 29% yield. ^1^H NMR (600 MHz, Chloroform-*d*) δ 9.29 (dt, *J* = 7.1, 1.1 Hz, 1H), 8.30 (dt, *J* = 9.0, 1.1 Hz, 1H), 7.92–7.88 (m, 1H), 7.69–7.65 (m, 2H), 7.38–7.34 (m, 3H), 7.04–7.00 (m, 2H), 6.94–6.90 (m, 2H), 5.06 (s, 2H), 4.09 (q, *J* = 7.1 Hz, 2H), 3.82 (s, 3H), 0.94 (t, *J* = 7.2 Hz, 3H). ^13^C NMR (151 MHz, Chloroform-*d*) δ 167.15, 166.10, 160.95, 159.71, 149.79, 147.90, 138.62, 131.41, 131.32, 130.58, 129.41, 128.64, 122.52, 118.33, 114.79, 114.19, 103.61, 70.04, 61.83, 55.47, 13.70. HRMS (ESI): *m*/*z* calced for C_25_H_23_N_2_O_5_ [M + H]^+^: 431.16015, found: 431.16140.

#### 3.2.21. Ethyl 1-Oxo-3-(4-(pyridin-3-ylmethoxy)phenyl)-1H-pyrido[1,2-*c*]pyrimidine-4-carboxylate (**M12**)

Compound **M12** was prepared by a procedure similar to that for **M1**, affording 14 mg of a pale yellow powder in a 36% yield. ^1^H NMR (600 MHz, Chloroform-*d*) δ 9.28 (dt, *J* = 7.0, 1.2 Hz, 1H), 8.73 (d, *J* = 2.2 Hz, 1H), 8.64–8.59 (m, 1H), 8.31 (dt, *J* = 9.1, 1.1 Hz, 1H), 7.90–7.85 (m, 2H), 7.71–7.67 (m, 2H), 7.40 (dd, *J* = 7.8, 4.9 Hz, 1H), 7.34 (td, *J* = 6.9, 1.3 Hz, 1H), 7.04–7.01 (m, 2H), 5.17 (s, 2H), 4.10 (q, *J* = 7.1 Hz, 2H), 0.95 (t, *J* = 7.1 Hz, 3H). ^13^C NMR (151 MHz, Chloroform-*d*) δ 167.25, 166.66, 160.08, 150.27, 148.75, 148.15, 147.95, 138.28, 136.26, 132.81, 131.29, 130.61, 128.74, 124.02, 122.47, 118.19, 114.61, 103.57, 67.55, 61.74, 13.73. HRMS (ESI): *m*/*z* calced for C_23_H_20_N_3_O_4_ [M + H]^+^: 402.14483, found: 402.14530.

#### 3.2.22. Ethyl 3-(4-(Cyclohexyloxy)phenyl)-1-oxo-1H-pyrido[1,2-*c*]pyrimidine-4-carboxylate (**M13**)

Compound **M13** was prepared by a procedure similar to that for **M1**, affording 18 mg of a pale yellow powder in a 47% yield. ^1^H NMR (600 MHz, Chloroform-*d*) δ 9.30 (d, *J* = 6.9 Hz, 1H), 8.31 (d, *J* = 8.9 Hz, 1H), 7.94 (t, *J* = 7.7 Hz, 1H), 7.69–7.64 (m, 2H), 7.40 (t, *J* = 5.6 Hz, 1H), 6.97–6.92 (m, 2H), 4.34 (tt, *J* = 8.7, 3.8 Hz, 1H), 4.11 (q, *J* = 7.1 Hz, 2H), 2.00 (dd, *J* = 13.2, 5.2 Hz, 2H), 1.81 (ddd, *J* = 10.1, 6.5, 3.2 Hz, 2H), 1.63–1.54 (m, 2H), 1.43–1.31 (m, 4H), 0.97 (t, *J* = 7.1 Hz, 3H). ^13^C NMR (151 MHz, Chloroform-*d*) δ 166.97, 160.36, 147.90, 139.15, 131.62, 130.75, 130.72, 122.62, 118.62, 115.69, 103.67, 61.96, 31.77, 25.70, 23.82, 13.67. HRMS (ESI): *m*/*z* calced for C_23_H_25_N_2_O_4_ [M + H]^+^: 393.18088, found: 393.18096.

#### 3.2.23. Ethyl 3-(4-(Cyclopentyloxy)phenyl)-1-oxo-1H-pyrido[1,2-*c*]pyrimidine-4-carboxylate (**M14**)

Compound **M14** was prepared by a procedure similar to that for **M1**, affording 13 mg of a pale yellow powder in a 35% yield. ^1^H NMR (600 MHz, Chloroform-*d*) δ 9.29 (dt, *J* = 6.9, 1.2 Hz, 1H), 8.30 (dt, *J* = 9.0, 1.1 Hz, 1H), 7.90 (ddd, *J* = 8.7, 6.8, 1.4 Hz, 1H), 7.67–7.64 (m, 2H), 7.36 (td, *J* = 6.9, 1.3 Hz, 1H), 6.94–6.90 (m, 2H), 4.83 (tt, *J* = 6.0, 2.7 Hz, 1H), 4.11 (q, *J* = 7.2 Hz, 2H), 1.98–1.90 (m, 2H), 1.88–1.76 (m, 4H), 1.69–1.58 (m, 2H), 0.97 (t, *J* = 7.1 Hz, 3H). ^13^C NMR (151 MHz, Chloroform-*d*) δ 167.17, 160.50, 149.64, 147.90, 138.72, 131.46, 130.60, 130.37, 122.52, 118.34, 115.40, 103.57, 61.86, 32.97, 24.20, 13.70. HRMS (ESI): *m*/*z* calced for C_22_H_23_N_2_O_4_ [M + H]^+^: 379.16523, found: 379.16651.

#### 3.2.24. Ethyl 3-(4-((2,6-Dimethylphenyl)amino)phenyl)-1-oxo-6-(trimethylsilyl)-1H-pyrido[1,2-*c*]pyrimidine-4-carboxylate (**N1**)

To a 10 mL single-necked flask, **J2** (50 mg, 1.0 eq.), 2,6-dimethylaniline (17 µL, 1.2 eq.), Pd_2_(dba)_3_ (5 mg, 0.05 eq.), Xantphos (13 mg, 0.2 eq.), Cs_2_CO_3_ (44 mg, 1.2 eq.), and toluene (2 mL) were added. The flask was purged with N_2_, and the mixture was stirred at 100 °C for 12 h until the reaction was complete, as monitored by TLC. The reaction mixture was filtered, washed with EA, and the filtrate was concentrated. Purification by preparative TLC afforded 30.2 mg of compound **N1** as a yellow solid in a 55% yield. ^1^H NMR (600 MHz, Chloroform-*d*) δ 9.14–9.12 (m, 1H), 8.32 (d, *J* = 1.1 Hz, 1H), 7.56 (d, *J* = 8.4 Hz, 2H), 7.32 (dd, *J* = 6.9, 1.2 Hz, 1H), 7.13 (d, *J* = 2.0 Hz, 3H), 6.52–6.49 (m, 2H), 5.47 (s, 1H), 4.14 (q, *J* = 7.2 Hz, 2H), 2.21 (s, 6H), 1.01 (t, *J* = 7.2 Hz, 3H), 0.37 (s, 9H). ^13^C NMR (151 MHz, Chloroform-*d*) δ 167.61, 148.65, 145.79, 137.09, 136.41, 130.61, 128.65, 126.91, 126.54, 121.11, 112.56, 102.58, 61.50, 18.26, 13.57, −2.05. HRMS (ESI): *m*/*z* calced for C_28_H_32_N_3_O_3_Si [M + H]^+^: 486.2207, found: 486.2196. Compounds **N2**–**N4** were prepared according to the synthetic procedure of compound **N1**.

#### 3.2.25. Ethyl 3-(4-((2,6-Dichlorophenyl)amino)phenyl)-1-oxo-6-(trimethylsilyl)-1H-pyrido[1,2-*c*]pyrimidine-4-carboxylate (**N2**)

Compound **N2** was prepared by a procedure similar to that for **N1**, affording 36.5 mg of a yellow solid in a 62% yield. ^1^H NMR (600 MHz, Chloroform-*d*) δ 9.16 (dd, *J* = 6.9, 0.9 Hz, 1H), 8.37 (d, *J* = 1.1 Hz, 1H), 7.62–7.59 (m, 2H), 7.40 (d, *J* = 8.1 Hz, 2H), 7.36 (dd, *J* = 6.9, 1.2 Hz, 1H), 7.12 (t, *J* = 8.1 Hz, 1H), 6.72–6.69 (m, 2H), 5.94 (s, 1H), 4.12 (q, *J* = 7.2 Hz, 2H), 0.99 (t, *J* = 7.2 Hz, 3H), 0.38 (s, 9H). ^13^C NMR (151 MHz, Chloroform-d) δ 167.42, 145.82, 145.68, 135.64, 132.10, 129.97, 128.96, 128.68, 127.03, 126.29, 121.45, 115.07, 102.92, 61.64, 13.55, -2.04. HRMS (ESI): *m*/*z* calced for C_26_H_26_Cl_2_N_3_O_3_Si [M + H]^+^: 526.1115, found: 526.1098.

#### 3.2.26. Ethyl 3-(4-((2-Methoxy-4-methylphenyl)amino)phenyl)-1-oxo-6-(trimethylsilyl)-1H-pyrido[1,2-*c*]pyrimidine-4-carboxylate (**N3**)

Compound **N3** was prepared by a procedure similar to that for **N1**, affording 32.7 mg of a yellow solid in a 58% yield. ^1^H NMR (600 MHz, Chloroform-*d*) δ 9.15 (d, *J* = 6.8 Hz, 1H), 8.33 (s, 1H), 7.66–7.62 (m, 2H), 7.34 (dd, *J* = 6.9, 1.3 Hz, 1H), 7.26 (d, *J* = 8.5 Hz, 2H), 7.09–7.06 (m, 2H), 6.76–6.72 (m, 2H), 4.18 (q, *J* = 7.1 Hz, 2H), 3.87 (s, 3H), 2.34 (s, 3H), 1.06 (t, *J* = 7.1 Hz, 3H), 0.38 (s, 9H). ^13^C NMR (151 MHz, Chloroform-*d*) δ 167.51, 149.45, 146.03, 145.77, 131.76, 130.42, 128.68, 128.41, 126.99, 121.29, 121.01, 117.53, 115.57, 111.89, 102.84, 61.62, 55.61, 21.28, 13.73, −2.04. HRMS (ESI): *m*/*z* calced for C_28_H_32_N_3_O_4_Si [M + H]^+^: 502.2156, found: 502.2142.

#### 3.2.27. Ethyl 3-(4-((2-Chloro-4-(trifluoromethoxy)phenyl)amino)phenyl)-1-oxo-6-(Trimethylsilyl)-1H-pyrido[1,2-*c*]pyrimidine-4-carboxylate (**N4**)

Compound **N4** was prepared by a procedure similar to that for **N1**, affording 21 mg of a yellow solid in a 33% yield. ^1^H NMR (600 MHz, Chloroform-*d*) δ 9.20 (dt, *J* = 6.9, 0.9 Hz, 1H), 8.39 (t, *J* = 1.0 Hz, 1H), 7.71–7.67 (m, 2H), 7.41 (dt, *J* = 7.0, 1.4 Hz, 1H), 7.35 (d, *J* = 9.0 Hz, 1H), 7.31 (d, *J* = 2.7 Hz, 1H), 7.18–7.15 (m, 2H), 7.09–7.06 (m, 1H), 6.23 (s, 1H), 4.17 (q, *J* = 7.1 Hz, 2H), 1.05 (t, *J* = 7.1 Hz, 3H), 0.39 (s, 9H). ^13^C NMR (151 MHz, Chloroform-*d*) δ 167.14, 145.83, 143.33, 142.30, 138.22, 130.38, 128.77, 127.13, 123.18, 122.68, 121.82, 120.75, 118.21, 117.01, 103.10, 61.61, 13.71, −2.04. HRMS (ESI): *m*/*z* calced for C_27_H_26_ClF_3_N_3_O_4_Si [M + H]^+^: 576.1328, found: 576.1328.

#### 3.2.28. Ethyl 1-Oxo-3-(4-(phenylamino)phenyl)-1H-pyrido[1,2-*c*]pyrimidine-4-carboxylate (**O1**)

To a 10 mL single-necked flask were added K_2_ (30 mg, 1.0 eq.), aniline (9 mg, 1.2 eq.), Pd_2_(dba)_3_ (4 mg, 0.05 eq.), Xantphos (9 mg, 0.2 eq.), Cs_2_CO_3_ (32 mg, 1.2 eq.), and toluene (2 mL). The flask was purged with N_2_, and the mixture was stirred at 100 °C for 12 h until the reaction was complete, as monitored by TLC. The reaction mixture was filtered, washed with ethyl acetate (EA), and the filtrate was concentrated. Purification by preparative TLC afforded 18 mg of the product as a yellow solid in a 58% yield. ^1^H NMR (600 MHz, Chloroform-*d*) δ 9.25 (dt, *J* = 7.0, 1.1 Hz, 1H), 8.26 (dt, *J* = 9.0, 1.1 Hz, 1H), 7.85 (ddd, *J* = 8.6, 6.7, 1.4 Hz, 1H), 7.65–7.62 (m, 2H), 7.34–7.28 (m, 3H), 7.17–7.13 (m, 2H), 7.08–7.05 (m, 2H), 7.01 (tt, *J* = 7.3, 1.1 Hz, 1H), 4.15 (q, *J* = 7.1 Hz, 2H), 1.04 (t, *J* = 7.1 Hz, 3H). ^13^C NMR (151 MHz, Chloroform-*d*) δ 167.36, 150.01, 147.74, 145.85, 141.57, 138.04, 131.15, 130.47, 129.47, 122.36, 122.22, 119.38, 117.74, 115.58, 103.13, 61.65, 13.69. HRMS (ESI): *m*/*z* calced for C_23_H_20_N_3_O_3_ [M + H]^+^: 386.1499, found: 386.1482. Compounds **O2**–**O7** were prepared according to the synthetic procedure of compound **O1**.

#### 3.2.29. Ethyl 3-(4-((2-Chloro-4-(trifluoromethoxy)phenyl)amino)phenyl)-1-oxo-1H-pyrido[1,2-*c*]pyrimidine-4-carboxylate (**O2**)

Compound **O2** was prepared by a procedure similar to that for **O1**, affording 6.4 mg of a yellow solid in a 43% yield. ^1^H NMR (600 MHz, Chloroform-*d*) δ 9.31 (d, *J* = 7.0 Hz, 1H), 8.33 (d, *J* = 9.0 Hz, 1H), 7.91 (ddd, *J* = 8.7, 6.8, 1.5 Hz, 1H), 7.74–7.69 (m, 2H), 7.38–7.35 (m, 2H), 7.33 (d, *J* = 2.7 Hz, 1H), 7.20–7.17 (m, 2H), 7.10 (dd, *J* = 8.9, 2.7 Hz, 1H), 6.25 (s, 1H), 4.17 (q, *J* = 7.1 Hz, 2H), 1.05 (t, *J* = 7.1 Hz, 3H). ^13^C NMR (151 MHz, Chloroform-*d*) δ 167.12, 166.28, 150.08, 147.83, 143.36, 142.32, 142.31, 138.19, 133.21, 131.20, 130.38, 123.19, 122.69, 122.33, 120.75, 118.18, 118.05, 116.98, 103.31, 61.63, 13.68. HRMS (ESI): *m*/*z* calced for C_24_H_18_ClF_3_N_3_O_4_ [M + H]^+^: 504.0932, found: 504.0916.

#### 3.2.30. Ethyl 3-(4-((2-Methoxy-4-methylphenyl)amino)phenyl)-1-oxo-1H-pyrido[1,2-*c*]pyrimidine-4-carboxylate (**O3**)

Compound **O3** was prepared by a procedure similar to that for **O1**, affording 8 mg of a yellow solid in a 31% yield. ^1^H NMR (600 MHz, Chloroform-*d*) δ 9.24 (d, *J* = 7.0 Hz, 1H), 8.25 (d, *J* = 9.0 Hz, 1H), 7.83 (ddd, *J* = 8.5, 6.8, 1.4 Hz, 1H), 7.64 (d, *J* = 8.5 Hz, 2H), 7.28 (d, *J* = 7.1 Hz, 1H), 7.08 (d, *J* = 8.6 Hz, 2H), 6.74 (d, *J* = 6.5 Hz, 2H), 4.15 (q, *J* = 7.1 Hz, 2H), 3.87 (s, 3H), 2.34 (s, 3H), 1.04 (t, *J* = 7.1 Hz, 3H). ^13^C NMR (151 MHz, Chloroform-*d*) δ 167.51, 166.38, 150.14, 149.45, 147.72, 146.05, 137.84, 131.79, 131.10, 130.43, 130.07, 128.38, 122.15, 121.01, 117.56, 117.51, 115.54, 111.90, 103.07, 61.64, 55.61, 21.28, 13.70. HRMS (ESI): *m*/*z* calced for C_25_H_24_N_3_O_4_ [M + H]^+^: 430.1761, found: 430.1747.

#### 3.2.31. Ethyl 3-(4-((2,6-Dichlorophenyl)amino)phenyl)-1-oxo-1H-pyrido[1,2-*c*]pyrimidine-4-carboxylate (**O4**)

Compound **O4** was prepared by a procedure similar to that for **O1**, affording 4 mg of a yellow solid in a 17% yield. ^1^H NMR (600 MHz, Chloroform-d) δ 9.38 (t, J = 7.2 Hz, 1H), 8.72 (d, J = 5.9 Hz, 1H), 7.94 (d, J = 7.0 Hz, 1H), 7.51–7.46 (m, 3H), 7.42 (d, J = 7.8 Hz, 1H), 7.19 (q, J = 7.8 Hz, 2H), 6.71 (t, J = 7.4 Hz, 2H), 4.17 (p, J = 7.0 Hz, 2H), 0.99 (q, J = 7.2 Hz, 3H). HRMS (ESI): *m*/*z* calced for C_23_H_18_Cl_2_N_3_O_3_ [M + H]^+^: 454.0719, found: 454.0717.

#### 3.2.32. Ethyl 3-(4-((2,6-Dimethylphenyl)amino)phenyl)-1-oxo-1H-pyrido[1,2-*c*]pyrimidine-4-carboxylate (**O5**)

Compound **O5** was prepared by a procedure similar to that for **O1**, affording 4.3 mg of a yellow solid in a 22% yield. ^1^H NMR (600 MHz, Chloroform-*d*) δ 9.23 (d, *J* = 7.0 Hz, 1H), 8.25 (d, *J* = 9.0 Hz, 1H), 7.82 (ddd, *J* = 8.8, 6.8, 1.4 Hz, 1H), 7.57 (d, *J* = 8.4 Hz, 2H), 7.28–7.26 (m, 1H), 7.15–7.13 (m, 3H), 6.51 (d, *J* = 8.4 Hz, 2H), 4.12 (q, *J* = 7.2 Hz, 2H), 2.21 (s, 6H), 1.00 (t, *J* = 7.1 Hz, 3H). ^13^C NMR (151 MHz, Chloroform-*d*) δ167.52, 148.76, 147.73, 137.90, 137.02, 136.41, 131.13, 130.67, 128.67, 126.59, 122.12, 117.49, 112.55, 102.85, 61.55, 18.25, 13.54. HRMS (ESI): *m*/*z* calced for C_25_H_24_N_3_O_3_ [M + H]^+^: 414.1812, found: 414.1803.

#### 3.2.33. Ethyl 3-(4-(Benzylamino)phenyl)-1-oxo-1H-pyrido[1,2-*c*]pyrimidine-4-carboxylate (**O6**)

Compound **O6** was prepared by a procedure similar to that for **O1**, affording 6 mg of a yellow solid in a 19% yield. ^1^H NMR (600 MHz, Chloroform-*d*) δ 9.23–9.20 (m, 1H), 8.21 (dt, *J* = 9.0, 1.1 Hz, 1H), 7.79 (ddd, *J* = 9.1, 6.8, 1.5 Hz, 1H), 7.61–7.58 (m, 2H), 7.36–7.33 (m, 4H), 7.30–7.27 (m, 2H), 7.25–7.22 (m, 1H), 6.65–6.63 (m, 2H), 4.41 (s, 2H), 4.12 (q, *J* = 7.1 Hz, 2H), 1.01 (t, *J* = 7.1 Hz, 3H). ^13^C NMR (151 MHz, Chloroform-*d*) δ 167.71, 166.63, 150.24, 150.12, 147.67, 138.67, 137.58, 131.03, 130.66, 128.82, 128.72, 127.87, 127.84, 127.43, 127.41, 122.03, 117.26, 112.11, 102.84, 61.55, 47.78, 29.71, 18.46, 13.68. HRMS (ESI): *m*/*z* calced for C_24_H_22_N_3_O_3_ [M + H]^+^: 400.1656, found: 400.1638.

#### 3.2.34. Ethyl 3-(4-((4-Chlorobenzyl)amino)phenyl)-1-oxo-1H-pyrido[1,2-*c*]pyrimidine-4-carboxylate (**O7**)

Compound **O7** was prepared by a procedure similar to that for **O1**, affording 9 mg of a yellow solid in a 26% yield. ^1^H NMR (600 MHz, Chloroform-*d*) δ 9.22 (dt, *J* = 7.0, 1.2 Hz, 1H), 8.21 (dt, *J* = 9.1, 1.1 Hz, 1H), 7.80 (ddd, *J* = 8.7, 6.8, 1.5 Hz, 1H), 7.60–7.57 (m, 2H), 7.32–7.27 (m, 4H), 7.24 (dd, *J* = 7.0, 1.3 Hz, 1H), 6.63–6.60 (m, 2H), 4.40–4.37 (m, 2H), 4.11 (q, *J* = 7.1 Hz, 2H), 0.99 (t, *J* = 7.1 Hz, 3H). ^13^C NMR (151 MHz, Chloroform-*d*) δ 167.64, 166.57, 150.23, 147.67, 137.64, 137.25, 133.11, 131.03, 130.64, 128.83, 128.63, 128.16, 122.06, 117.35, 112.17, 102.87, 61.54, 47.09, 13.65. HRMS (ESI): *m*/*z* calced for C_24_H_21_ClN_3_O_3_ [M + H]^+^: 434.1266, found: 434.1245.

#### 3.2.35. N-(((1s,3s)-Adamantan-1-yl)methyl)-3-(4-bromophenyl)-1-oxo-1H-pyrido[1,2-*c*]pyrimidine-4-carboxamide (**P1**)

Compound **K2** (30 mg, 1.0 eq.), ((1s,3s)-adamantan-1-yl)methanamine (16 mg, 1.2 eq.), Cs_2_CO_3_ (32 mg, 1.2 eq.), and THF (2 mL) were added to a 10 mL single-necked flask. The mixture was stirred at reflux at 70 °C for 12 h, and the reaction was monitored by TLC until completion. Water and ethyl acetate (EA) were added to the reaction mixture for liquid–liquid extraction. The organic phase was washed with saturated aqueous NaCl solution, dried over anhydrous sodium sulfate, filtered, and concentrated. Purification by preparative thin-layer chromatography (DCM/MeOH = 30/1) afforded 12.7 mg of compound **P1** as a yellow solid, with a yield of 32%. ^1^H NMR (600 MHz, Chloroform-*d*) δ 8.47–8.45 (m, 1H), 7.69–7.63 (m, 1H), 7.63–7.57 (m, 1H), 7.44–7.41 (m, 2H), 7.38 (d, *J* = 8.1 Hz, 1H), 7.20 (d, *J* = 8.2 Hz, 1H), 7.13 (d, *J* = 8.1 Hz, 2H), 3.72 (q, *J* = 7.0 Hz, 2H), 1.95–1.91 (m, 3H), 1.69–1.59 (m, 9H), 1.24 (t, *J* = 7.0 Hz, 4H). ^13^C NMR (151 MHz, Chloroform-*d*) δ 131.96, 130.13, 128.99, 58.50, 51.89, 41.09, 36.78, 35.93, 28.41, 18.45. HRMS (ESI): *m*/*z* calced for C_26_H_27_BrN_3_O_2_ [M + H]^+^: 492.1281, found: 492.1258.

#### 3.2.36. 3-(4-Bromophenyl)-N-cyclohexyl-1-oxo-1H-pyrido[1,2-*c*]pyrimidine-4-carboxamide (**P2**)

The preparation procedure was similar to that of compound **P1**, affording 12 mg of compound **P2** as a yellow solid, with a yield of 26%. ^1^H NMR (600 MHz, Chloroform-*d*) δ 9.82 (s, 1H), 8.51 (d, *J* = 5.0 Hz, 1H), 7.73 (s, 1H), 7.45–7.41 (m, 2H), 7.36 (s, 1H), 7.14 (d, *J* = 8.1 Hz, 2H), 4.80–4.73 (m, 1H), 2.25 (d, *J* = 12.8 Hz, 2H), 1.81 (d, *J* = 13.1 Hz, 2H), 1.70–1.61 (m, 3H), 1.33 (dddd, *J* = 16.9, 13.3, 8.5, 3.7 Hz, 2H), 1.14 (dtd, *J* = 13.2, 9.6, 3.6 Hz, 1H). ^13^C NMR (151 MHz, Chloroform-*d*) δ 151.88, 131.97, 130.20, 28.44, 26.28, 25.25. HRMS (ESI): *m*/*z* calced for C_21_H_21_BrN_3_O_2_ [M + H]^+^: 426.0812, found: 426.0792.

### 3.3. Biological Experimental Methods

#### 3.3.1. Cell and Virus

African green monkey kidney (Vero) cells were purchased (Procell, CN, Wuhan, China). Cells were preserved at 37 °C in 5% CO_2_ atmosphere as adherent culture in Dulbecco’s modified Eagle’s medium (DMEM/High glucose, Cytiva, Marlborough, MA, USA) mixed with 10% fetal bovine serum (FBS, TransGen Biotech, CN, Beijing, China). Upon reaching confluence of 90–100%, the cells were washed with PBS and harvested from the culture vessel surface using 0.06% trypsin.

The PEDV strain AJ1102 (GenBank accession No. JX188454) (isolated in China in 2011 from a sucking piglet suffering from acute diarrhea) was transfected into Vero cells in DMEM containing 10 µg·mL^−1^ trypsin [36].

#### 3.3.2. In Vitro Anti-PEDV Activity Evaluation

Vero cells were incubated in DMEM supplemented with 2% FBS for 2 h. Then the cells were infected with PEDV at a multiplicity of infection (MOI) of 0.01, and the infection was maintained at 37 °C for 1 h. After virus adsorption for 1h, the supernatant was discarded, and the cells were washed twice with DMEM, followed by incubation separately for 24 h. The antiviral effect of test compounds was evaluated using an RT-qPCR assay. Total RNA was extracted from cell samples with RNAiso Plus reagent (Takara Bio Inc., JPN, Kusatsu, Japan) and was transcribed into cDNA by using PrimeScript^TM^ FAST RT reagent Kit (Takara Bio Inc., JPN). The copy number of viral genomic RNA was detected with TB Green^®^ Premix Ex Taq^TM^ II (Tli RNaseH Plus) (Takara Bio Inc., JPN) and specific primer pairs (PEDV-N-F: 5′-GAATGCAAAACCCCAGAGAA-3′ and PEDV-N-R: 5′-GTGTCACCACCATCAACAGC-3′.

The titer of PEDV was determined using trypsin-containing DMEM via the 50% tissue culture infectious dose (TCID_50_) assay. Vero cells were seeded in 96-well plates and cultured at 37 °C under 5% CO_2_ until 90% confluence was attained. The viral suspension was serially diluted from 10^−1^ to 10^−10^ in 1.5 mL Eppendorf tubes on ice. A 100 µL volume of each dilution was added to columns 2 to 11 of the plate, with eight replicates per dilution. Columns 1 and 12 were used as uninfected negative controls. All experiments were performed in triplicate. Plates were incubated at 37 °C under 5% CO_2_, and the number of wells displaying cytopathic effect (CPE) was recorded daily until no additional CPE was observed. Viral titers were calculated using the Reed–Muench method.

#### 3.3.3. In Vitro Cytotoxicity Assay

Vero cells were seeded into 96-well cell culture plates and cultured until they formed a confluent monolayer. The concentrations of **J3** and **J4** were 0, 5, 10, 20, 40, 80, 160 µM; the concentrations of **J1** and **N1** were 0, 2.5, 5, 10, 20, 40, 80 µM; the concentrations of **N2** and **N4** were 0, 1, 2, 4, 8, 16, 32 µM. Normal cells were used as the blank group, and PEDV was added as the infection group. PEDV (MOI = 0.01) was added, and the cells were incubated for 2 h. After this, the maintenance medium supplemented with the same concentration of compounds was added, and the cells were cultured for 24 h. The medium was discarded, 10 μL of CCK-8 solution and 90 μL of medium were added to each well, the plate was placed in an incubator for 1–4 h, the absorbance of the samples was measured at a wavelength of 450 nm, and the data were recorded. Cell viability = (Treatment group OD value − Blank control group OD value)/(Negative control group OD value − Blank control group OD value) × 100%.

### 3.4. Microsomal Stability Assay

#### 3.4.1. Preparation of Solutions

Stock solution (10 mM) of test compounds was prepared in DMSO. The stock solution for the test compound was then diluted to 200 μM with acetonitrile.

#### 3.4.2. Microsome Incubations

Incubation mixtures were prepared in a total volume of 200 μL with final component concentrations as follows: 0.1 M PBS (pH 7.4), 3 mM MgCl_2_, NADPH (2 mM), liver microsomes (0.2 mg/mL) and test compound (1 μM) or positive control (1 μM). NADPH or buffer (negative control) was added after a 5-min preincubation of all other components at 37 °C. This was pipette-mixed to achieve a homogenous suspension and immediately transferred 20 µL incubate as a 0 min sample to wells in a “Quenching” plate, followed by pipette-mixing. At 5, 15, 30, and 60 min, pipette-mixed the incubate and serially transferred samples of 20 µL incubate per time point to wells in a separate “Quenching” plate, followed by pipette-mixing. In ‘Quenching’ plates, 200 µL of acetonitrile was added with IS.

#### 3.4.3. Sample Analysis

The 96-well plate was centrifuged at 4000 rpm, 4 °C for 10 min. 30 μL of supernatant was mixed with 90 μL of ddH_2_O and then injected onto the LC-MS/MS system for analysis.

## 4. Conclusions

In summary, building upon the novel PPO scaffold identified in our previous study, we designed and synthesized a diverse array of derivatives and systematically investigated the SAR governing their anti-PEDV potential. The incorporation of bulky hydrophobic substituents at the R_1_, R_2_, and R_3_ positions emerged as the core determinant for potent antiviral activity. Specifically, sterically demanding hydrophobic moieties at these sites—including aromatic rings at R_1_, adamantyl at R_2_, and trimethylsilyl (TMS) at R_3_—markedly enhanced antiviral efficacy. Furthermore, the steric and electronic properties of substituents on the R_1_ aromatic ring served as critical modulators, enabling fine-tuning of the antiviral activity within this scaffold. Among all synthesized derivatives, compounds **N1** and **N2** exhibited exceptional in vitro anti-PEDV potency with EC_50_ values of 0.32 μM and 0.37 μM and SI values of 43.78 and 42.89, respectively. However, both compounds exhibited high metabolism in human and mouse liver microsomes. Furthermore, although compound **J4** did not possess the most potent antiviral activity, it exhibited moderate metabolic stability in mouse liver microsomes along with low cytotoxicity. Further structural optimization of this series will be carried out to improve druggability, including metabolic stability and safety profiles.

## Figures and Tables

**Table 1:**
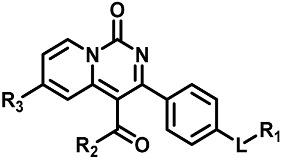
The Antiviral Activities of Target Compounds against PEDV.

Compd.	L	R_1_	R_2_	R_3_	Inhibition Rate (%) ^a^	Viral Yield Reduction ^b^
**J1**	O	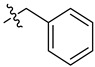	OEt	TMS	99.99 ± 0.01	4.49 ± 0.74
**J2**	Br	/	OEt	TMS	78.12 ± 10.18	0.70 ± 0.21
**J3**	O	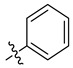	Me	TMS	99.99 ± 0.00	4.09 ± 0.13
**J4**	O	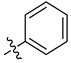	OEt	Ph	100.00 ± 0.00	4.64 ± 0.29
**J5**	O	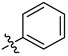	OEt	Me	93.47 ± 1.64	1.20 ± 0.10
**K1**	O	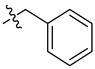	OEt	H	86.86 ± 5.45	0.92 ± 0.21
**K2**	Br	/	OEt	H	35.96 ± 9.37	0.20 ± 0.06
**K3**	O	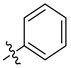	Me	H	98.46 ± 0.17	1.81 ± 0.04
**M0**	O	H	OEt	H	38.90 ± 3.49	0.22 ± 0.07
**M1**	O	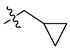	OEt	H	40.14 ± 9.44	0.23 ± 0.07
**M2**	O	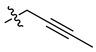	OEt	H	45.91 ± 7.23	0.27 ± 0.06
**M3**	O	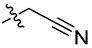	OEt	H	4.85 ± 1.38	0.02 ± 0.01
**M4**	O	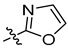	OEt	H	51.47 ±17.00	0.34 ± 0.16
**M5**	O	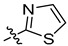	OEt	H	96.19 ± 2.19	1.48 ± 0.27
**M6**	O	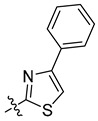	OEt	H	99.86 ± 0.03	2.85 ± 0.09
**M7**	O	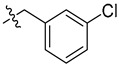	OEt	H	99.26 ± 0.11	2.14 ± 0.06
**M8**	O	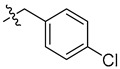	OEt	H	99.21 ± 0.23	2.12 ± 0.13
**M9**	O	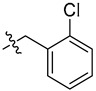	OEt	H	99.61 ± 0.04	2.41 ± 0.04
**M10**	O	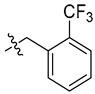	OEt	H	99.86 ± 0.04	2.87 ± 0.14
**M11**	O	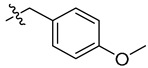	OEt	H	99.53 ± 0.05	2.33 ± 0.05
**M12**	O	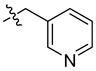	OEt	H	97.86 ± 1.93	1.92 ± 0.56
**M13**	O	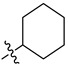	OEt	H	92.61 ± 2.22	1.15 ± 0.13
**M14**	O	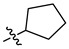	OEt	H	68.51 ± 13.12	0.53 ± 0.19
**N1**	NH	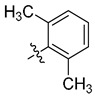	OEt	TMS	99.99 ± 0.01	3.88 ± 0.01
**N2**	NH	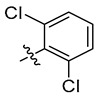	OEt	TMS	100.00 ± 0.00	4.48 ± 0.47
**N3**	NH	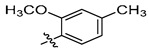	OEt	TMS	97.31 ± 0.85	1.59 ± 0.16
**N4**	NH	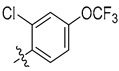	OEt	TMS	99.98 ± 0.01	3.78 ± 0.14
**O1**	NH	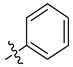	OEt	H	90.80 ± 2.08	1.05 ± 0.09
**O2**	NH	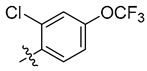	OEt	H	99.78 ± 0.08	2.68 ± 0.20
**O3**	NH	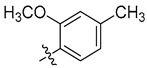	OEt	H	88.43 ± 1.72	0.94 ± 0.07
**O4**	NH	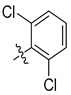	OEt	H	84.79 ± 6.82	0.86 ± 0.21
**O5**	NH	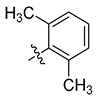	OEt	H	80.59 ± 4.97	0.72 ± 0.11
**O6**	NH	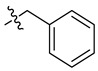	OEt	H	27.44 ± 10.94	0.14 ± 0.06
**O7**	NH	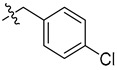	OEt	H	96.02 ± 3.29	1.60 ± 0.49
**P1**	Br	/	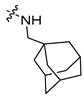	H	98.79 ± 0.89	2.11 ± 0.49
**P2**	Br	/	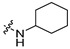	H	43.08 ± 6.28	0.25 ± 0.05
Lycorine ^c^	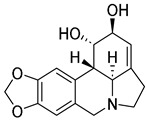				99.94 ± 0.04	3.69 ± 0.28

^a^ The target compounds were evaluated at a concentration of 10 μM, Values are given as mean ± SE of three replicates (*n* = 3). ^b^ Viral yield reduction, obtained from three replicates in independent experiments, is calculated based on the averaged log10-transformed virus titer of the mock-treated group divided by that of the compound-treated group. ^c^ The positive control and it was evaluated at a concentration of 3 μM.

**Table 2:** EC_50_, CC_50_, and SI of Preferred Compounds.

Compd.	Structure	EC_50_ (μM) ^a^	CC_50_ (μM) ^a^	SI ^b^
**J1**	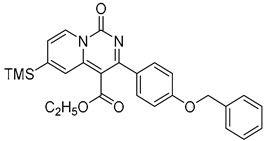	0.70 ± 0.06	10.80 ± 0.81	15.43
**J3**	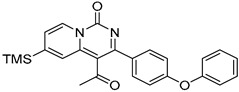	1.37 ± 0.07	18.28 ± 1.41	13.64
**J4**	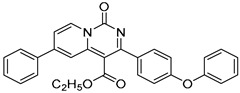	1.77 ± 0.20	60.47 ± 9.90	34.16
**N1**	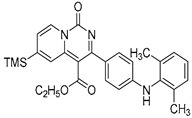	0.32 ± 0.02	14.01 ± 1.17	43.78
**N2**	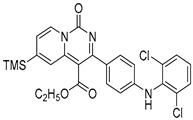	0.37 ± 0.02	15.87 ± 2.14	42.89
**N4**	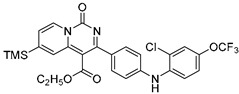	0.79 ± 0.08	18.16 ± 2.59	23.54

^a^ Values are given as mean ± SE of three replicates (*n* = 3). ^b^ SI = CC_50_/EC_50_.

**Table 3:** Microsomal Stability of the Preferred Compounds.

Compd.	Species	T_1/2_ (min)	Cl_int_ (mL/min/mg)	E_h_ (%)	Clearance Classified
**J1**	Human	12.7	0.273	93.9	High
	Mouse	8.58	0.404	94.7	High
**J3**	Human	30.4	0.114	86.6	High
	Mouse	72.7	0.0477	67.7	Medium
**J4**	Human	71.9	0.0482	73.1	High
	Mouse	92.0	0.0377	62.4	Medium
**N1**	Human	13.7	0.252	93.4	High
	Mouse	18.0	0.192	89.4	High
**N2**	Human	50.8	0.0682	79.4	High
	Mouse	39.0	0.0888	79.6	High

## Data Availability

All data presented in this study are available in the article and in the Supplementary Material.

## Supplementary Materials

The following supporting information can be downloaded at: https://www.mdpi.com/article/10.3390/molecules31091480/s1, Table S1: Structures of Target Compounds; ^1^H NMR, ^13^C NMR and HRMS Spectrums of target compounds; Analytical Report of HPLC Chromatograms of the Preferred Compounds; Dose–Response Curves.

## Abbreviations

The following abbreviations are used in this manuscript:

PEDV	Porcine Epidemic Diarrhea Virus
PPO	Pyrido[1,2-*c*]pyrimidin-1-one
SAR	Structure–Activity Relationships
PED	Porcine Epidemic Diarrhea
EC_50_	Half-maximal Inhibitory Concentration
SI	Selectivity Index
M^pro^	Main Protease
3CL^pro^	3C-like Protease
FIPV	Feline Infectious Peritonitis Virus
TMS	Trimethylsilyl
Ph	Phenyl
Me	Methyl
OEt	Ethoxy
THF	Tetrahydrofuran
TBAF	Tetra-n-butylammonium Fluoride
TFA	Trifluoroacetic Acid
DMF	N,N-Dimethylformamide
DIAD	Diisopropyl Azodicarboxylate
PPh_3_	Triphenylphosphine
BPO	Benzoyl Peroxide
HAuCl_4_·3H_2_O	Chloroauric Acid Trihydrate
Cs_2_CO_3_	Cesium Carbonate
Pd_2_(dba)_3_	Tris(dibenzylideneacetone)dipalladium(0)
DCE	1,2-Dichloroethane
Ac_2_O	Acetic Anhydride
Clint	Intrinsic Clearance
Eh	Hepatic Extraction Ratio
Vero	African green monkey kidney cells
DMEM	Dulbecco’s Modified Eagle Medium
FBS	Fetal Bovine Serum
PBS	Phosphate Buffered Saline
RT-qPCR	Real-time Quantitative Polymerase Chain Reaction
RNA	Ribonucleic Acid
cDNA	Complementary DNA
NMR	Nuclear Magnetic Resonance
HRMS	High-Resolution Mass Spectrometry
ESI	Electrospray Ionization
HPLC	High-Performance Liquid Chromatography
TLC	Thin-Layer Chromatography
MOI	Multiplicity of Infection
SD	Standard Deviation
NADPH	Reduced Nicotinamide Adenine Dinucleotide Phosphate
