# Supplementary material for: Design, Synthesis and Antiviral Evaluation of Pyrido[1,2-c]pyrimidin-1-one Derivatives Against Porcine Epidemic Diarrhea Virus (PEDV)
Source: Molecules. 2026 Apr 29;31(9):1480. doi: 10.3390/molecules31091480 (PMC13165446; doi:10.3390/molecules31091480)

Design, Synthesis and Antiviral Evaluation of Pyrido[1,2-*c*]pyrimidin-1-one Derivatives against Porcine Epidemic Diarrhea Virus (PEDV)

Wenlong Xu [1,2,†], Wu Ni [5,†], Ziyan Zhou [2,6,†], Zhenhui Ju [2,6], Sisi Liu[5,*], Shixiang Pan[2,3,*], and Xiangrui Jiang[2,3,4,*]

[1] School of Pharmacy, Anhui University of Chinese Medicine, Hefei, Anhui 230012, China.; wlxu@baridd.ac.cn

[2] Shandong Laboratory of Yantai Drug Discovery, Bohai Rim Advanced Research Institute for Drug Discovery, Yantai, Shandong 264117, China; zyzhou@baridd.ac.cn (Z.Z.); zhju@baridd.ac.cn (Z.J.)

[3] Shanghai Institute of Materia Medica, Chinese Academy of Sciences, Shanghai 201203, China; wuni@webmail.hzau.edu.cn

[4] University of Chinese Academy of Sciences, Beijing 100049, China;

[5] College of Chemistry, Huazhong Agricultural University, Wuhan, HuBei 430070, China;

[6] Key Laboratory of Structure-Based Drug Design and Discovery of Ministry of Education, Shenyang Pharmaceutical University, Shenyang, Liaoning 110016, China;

* Correspondence:
liusisi@mail.hzau.edu.cn(S.L.);
panshixiang@cau.edu.cn(S.P.);
jiangxiangrui@simm.ac.cn(X.J.).

† These authors contributed equally to this work.

Contents

1. Table S1. Structures of Target Compounds..S3
2. ^{1}H NMR, ^{13}C NMR, and HRMS..S5
3. Analytical Report of HPLC Chromatograms...S57
4. Dose-response curve..S62

Table S1. Structures of Target Compounds

Compd.	Structure	Compd.	Structure
**J1**	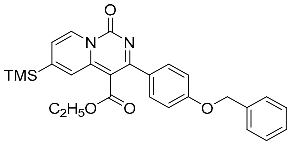	**M10**	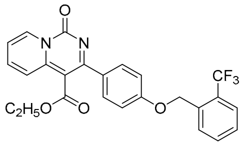
**J2**	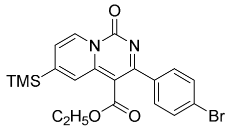	**M11**	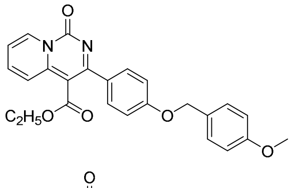
**J3**	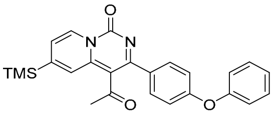	**M12**	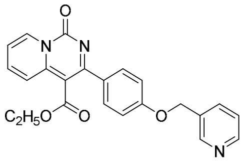
**J4**	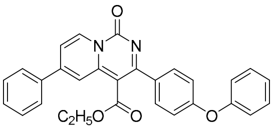	**M13**	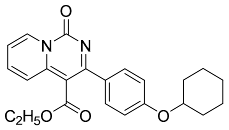
**J5**	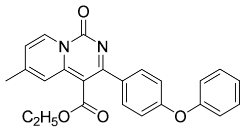	**M14**	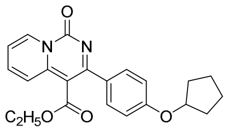
**K1**	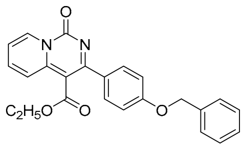	**N1**	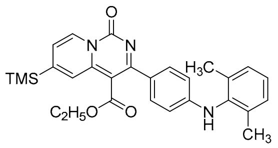
**K2**	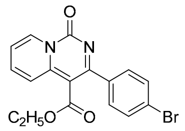	**N2**	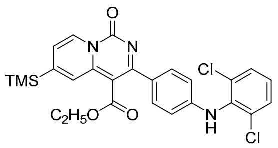
**K3**	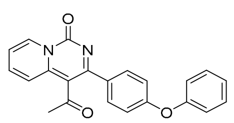	**N3**	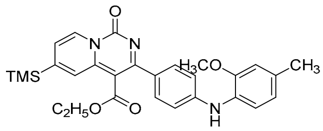
**M0**	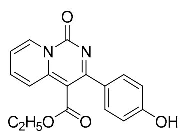	**N4**	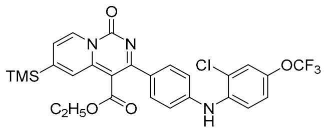
**M1**	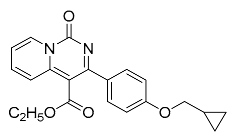	**O1**	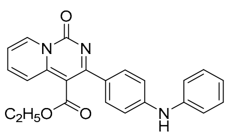

**M2**	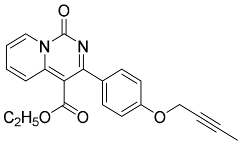	**O2**	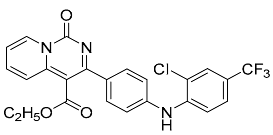
**M3**	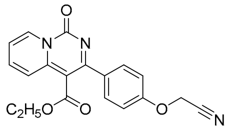	**O3**	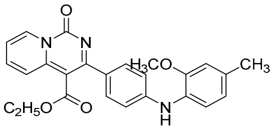
**M4**	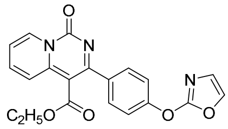	**O4**	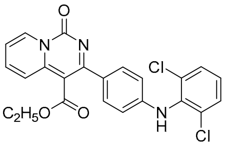
**M5**	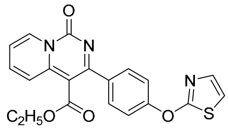	**O5**	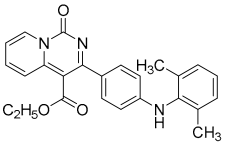
**M6**	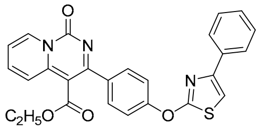	**O6**	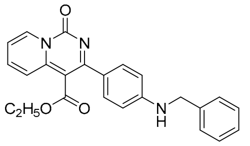
**M7**	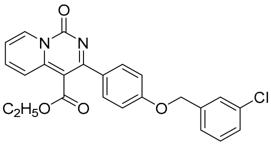	**O7**	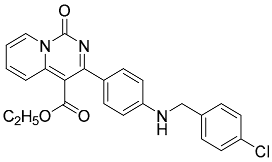
**M8**	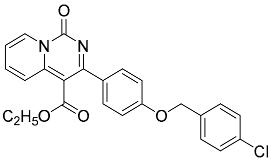	**P1**	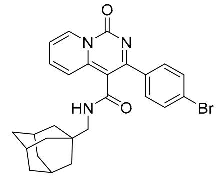
**M9**	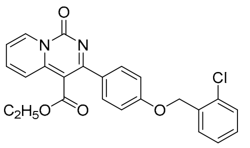	**P2**	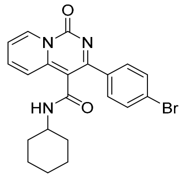

2. ^{1}H NMR, ^{13}C NMR and HRMS

2.1 ^{1}H NMR, ^{13}C NMR and HRMS of J1

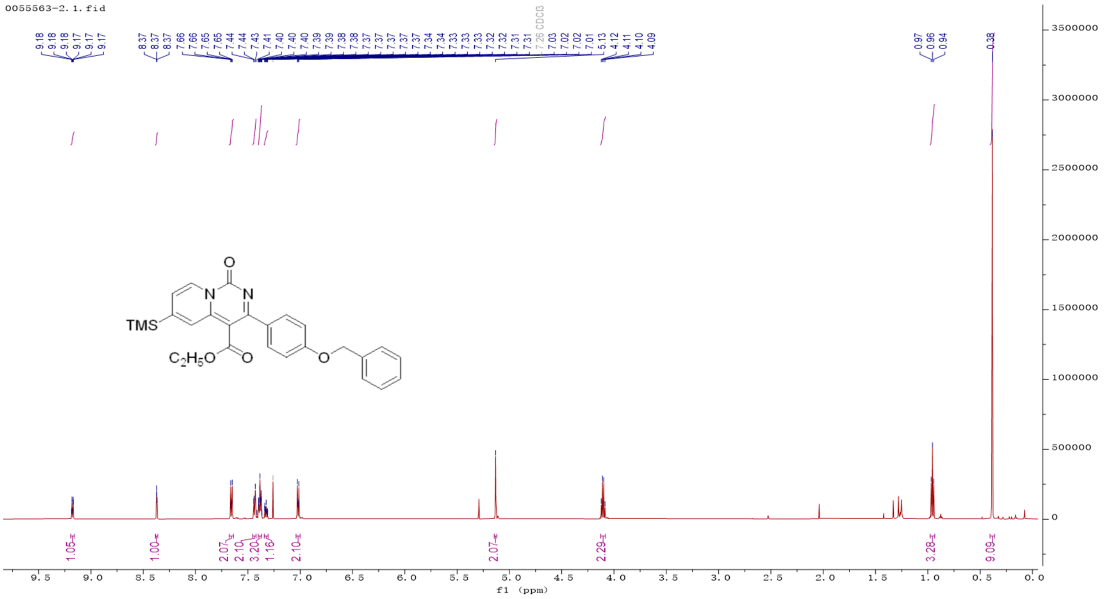

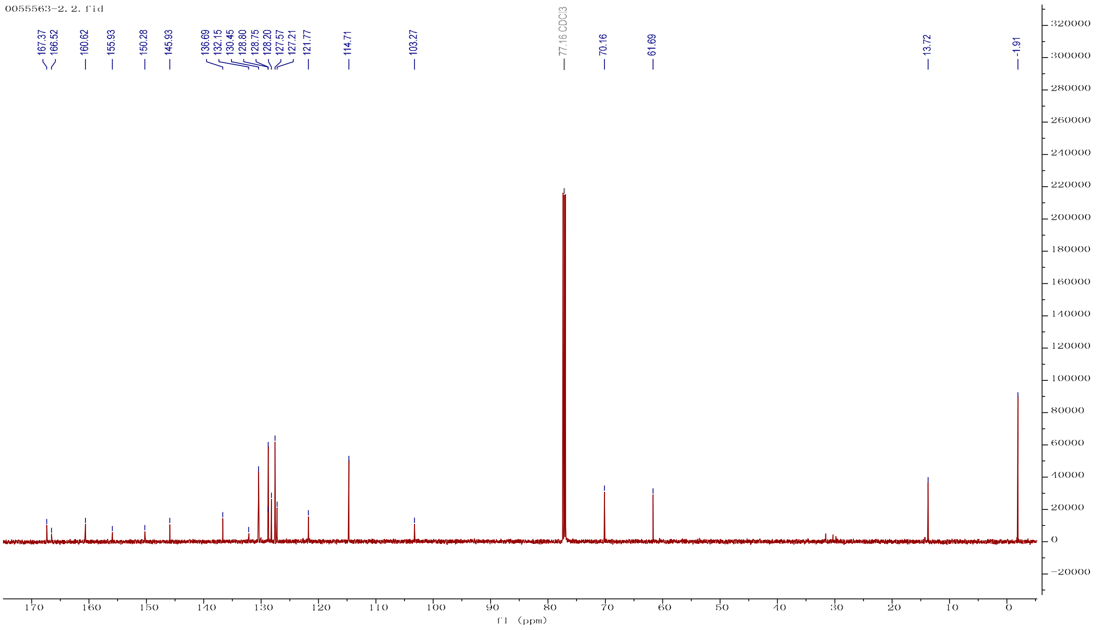

MS spectra

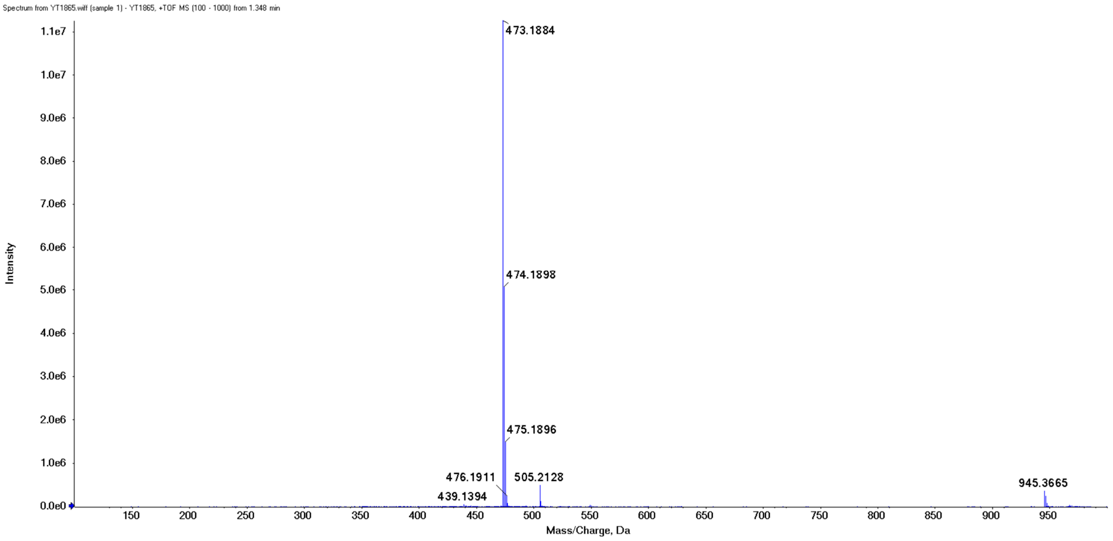

Formula Calculator Results

Measured m/z	Cal m/z	Error(mmu)	Error(ppm)	Ion Formula	Ion
473.18845	473.18911	-0.7	-1.4	$C_{27}H_{29}N_2O_4Si$	$[M+H]^+$

2.2 ^{1}H NMR and HRMS of J2

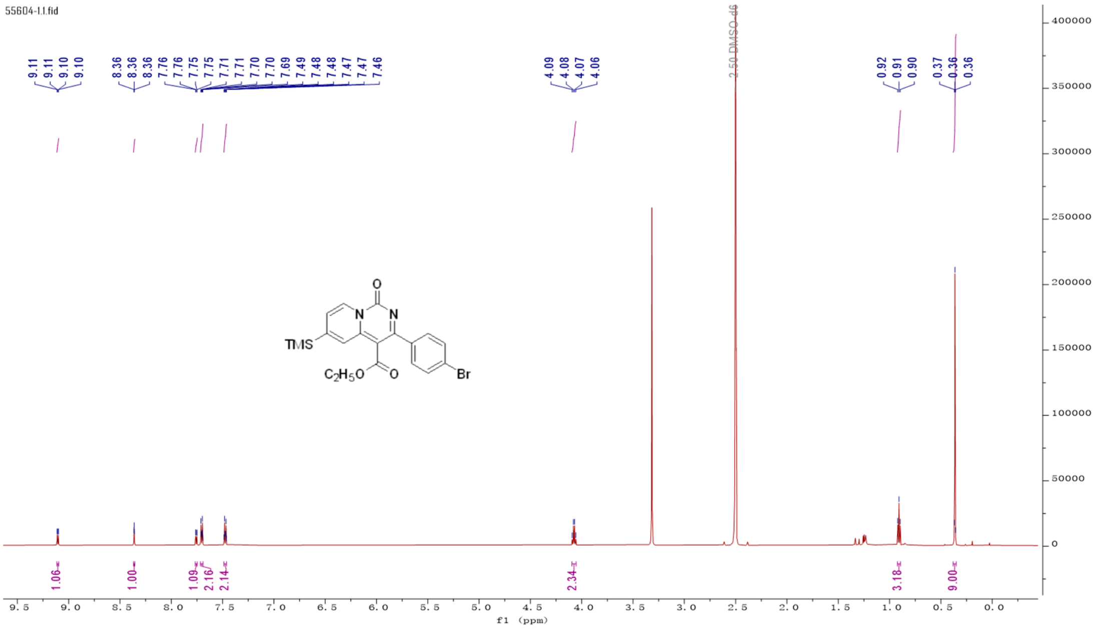

MS spectra

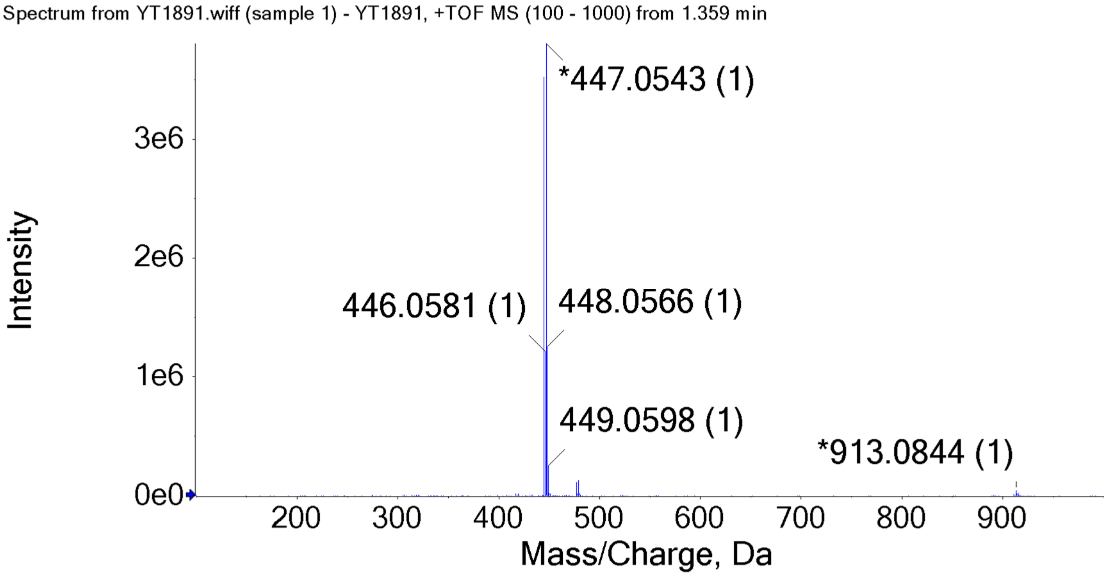

Formula Calculator Results

Measured m/z	Cal m/z	Error(mmu)	Error(ppm)	Ion Formula	Ion
445.0559	445.0577	-1.9	-4.3	$C_{20}H_{22}BrN_2O_3Si$	$[M+H]^+$

2.3 ^{1}H NMR, ^{13}C NMR and HRMS of J3

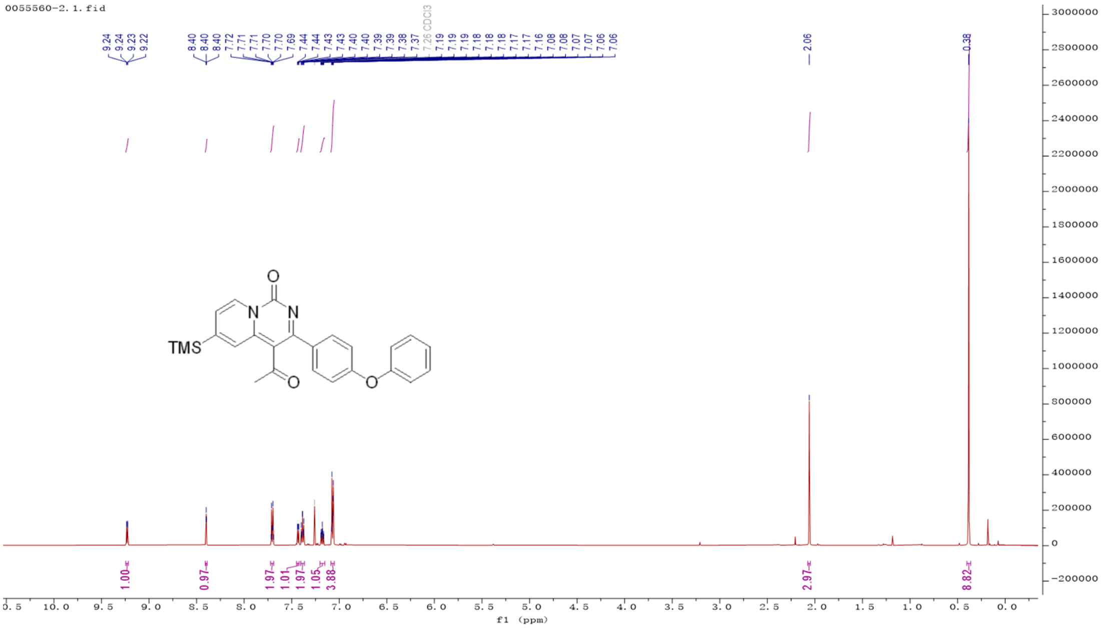

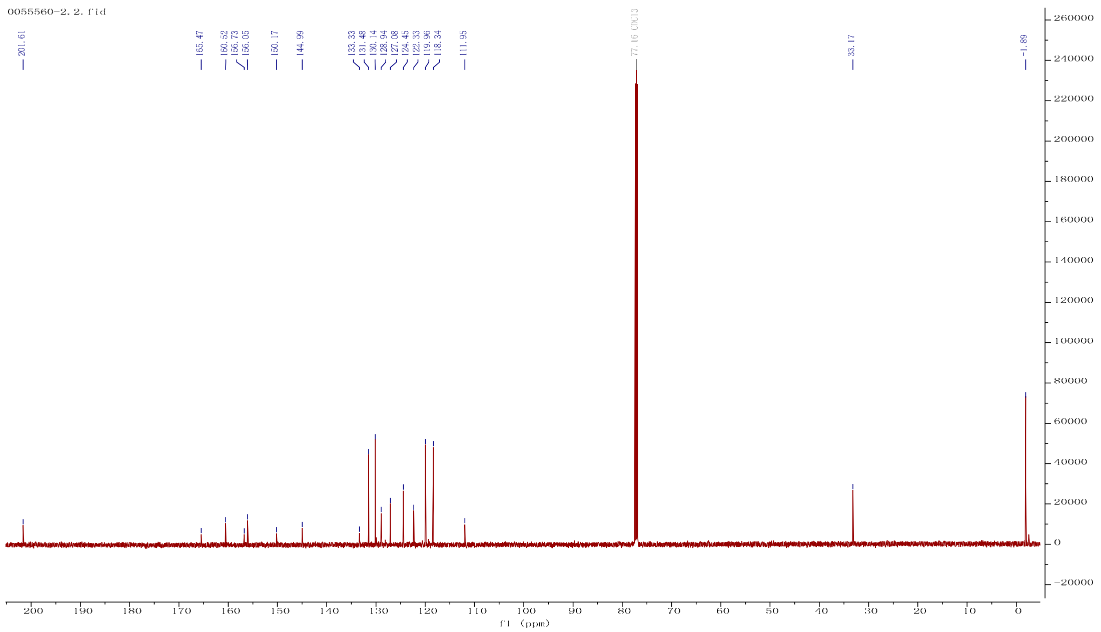

MS spectra

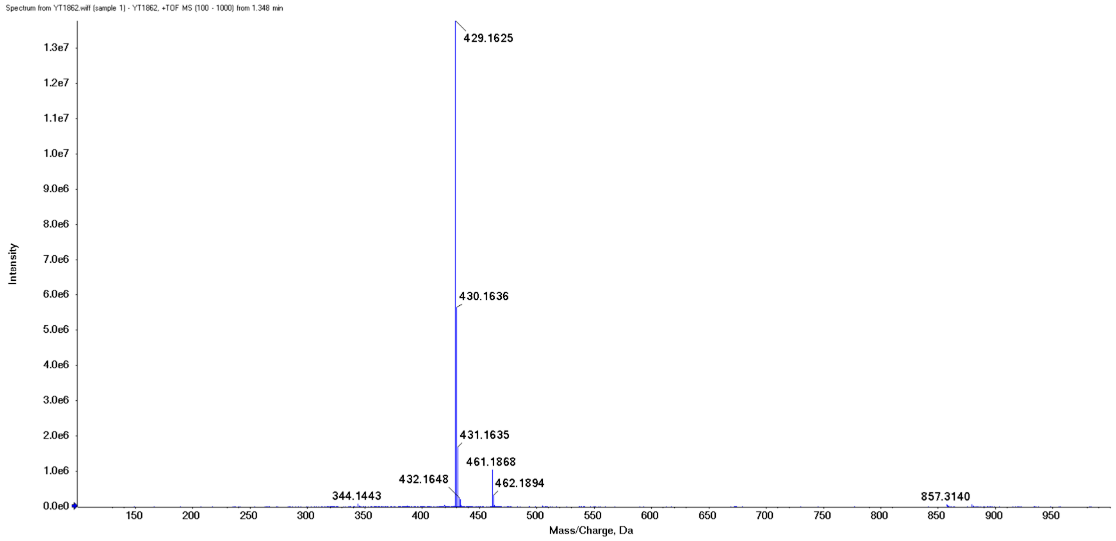

Formula Calculator Results

Measured m/z	Cal m/z	Error(mmu)	Error(ppm)	Ion Formula	Ion
429.16253	429.16290	-0.4	-0.9	$C_{25}H_{25}N_2O_3Si$	$[M+H]^+$

2.4 ^{1}H NMR, ^{13}C NMR and HRMS of J4

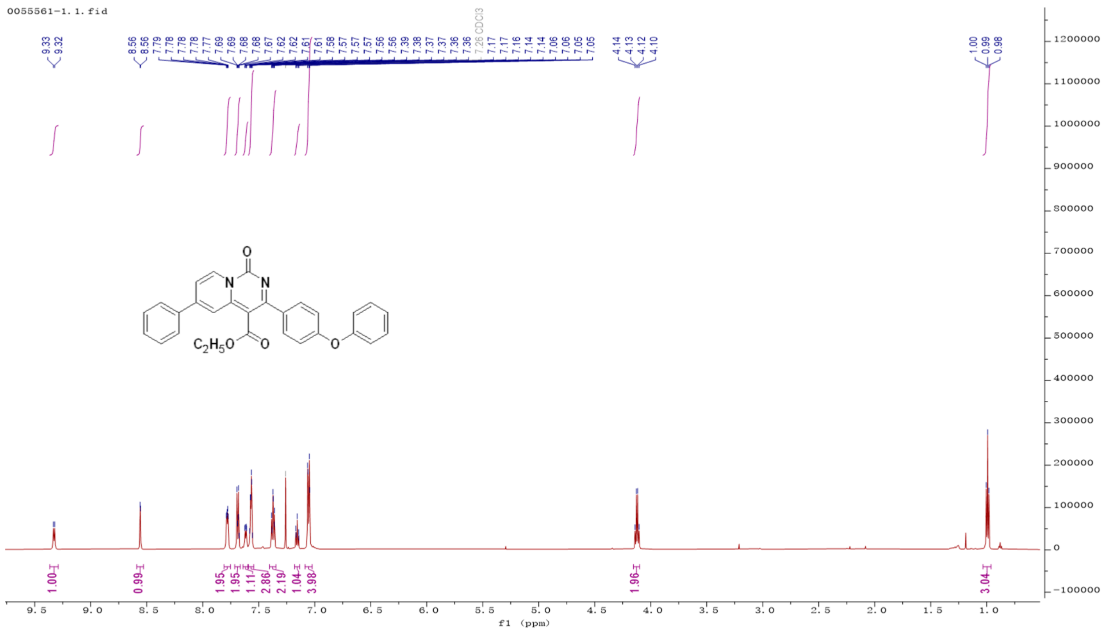

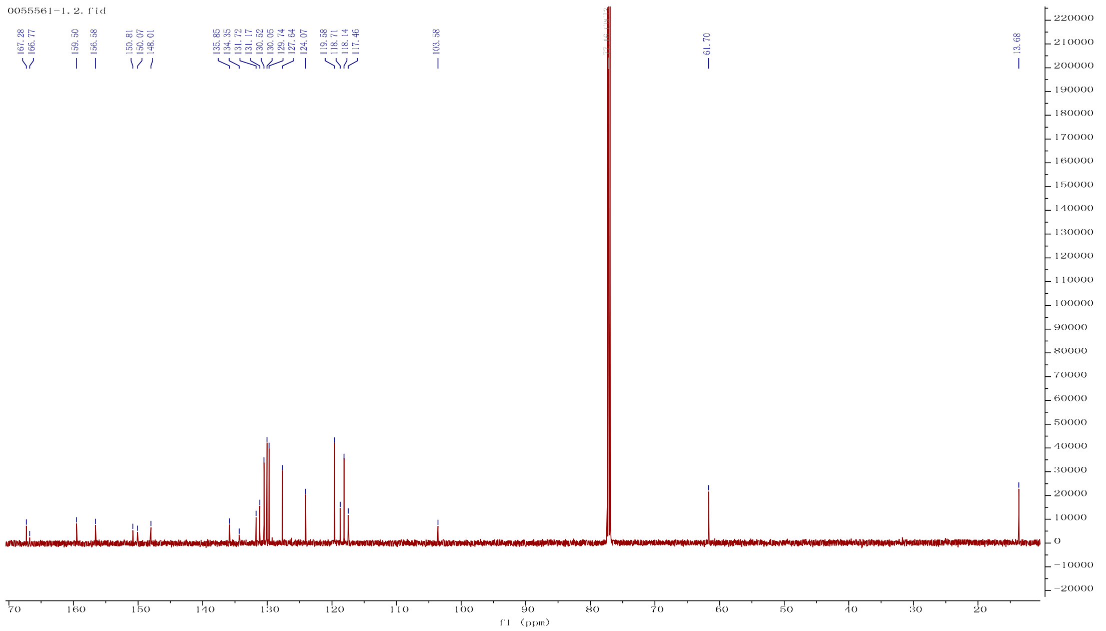

MS spectra

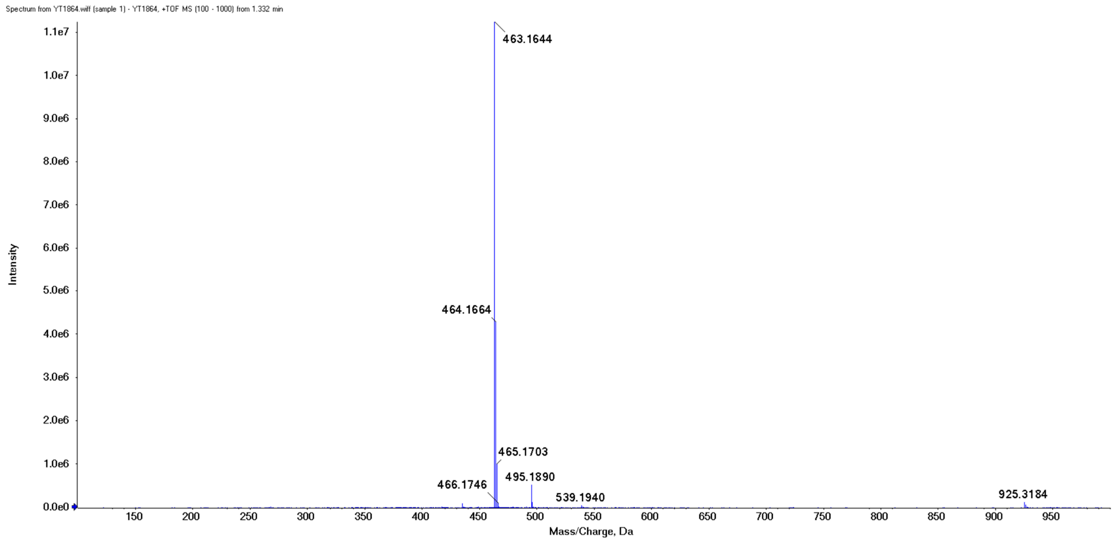

Formula Calculator Results

Measured m/z	Cal m/z	Error(mmu)	Error(ppm)	Ion Formula	Ion
463.16437	463.16523	-0.9	-1.9	$C_{29}H_{23}N_2O_4$	$[M+H]^+$

2.5 ^{1}H NMR, ^{13}C NMR and HRMS of J5

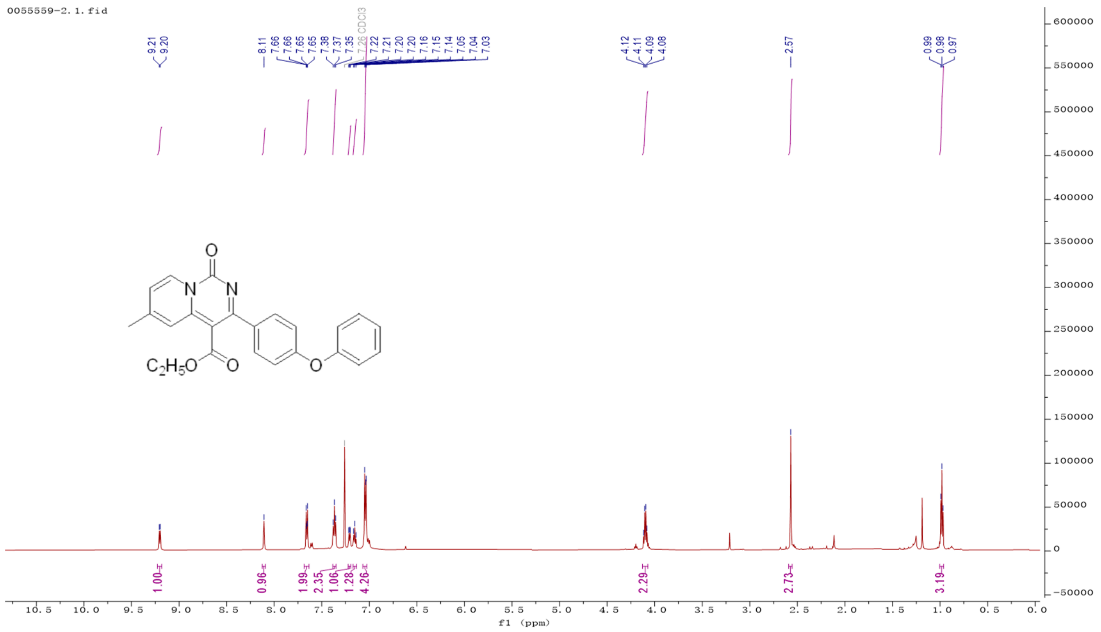

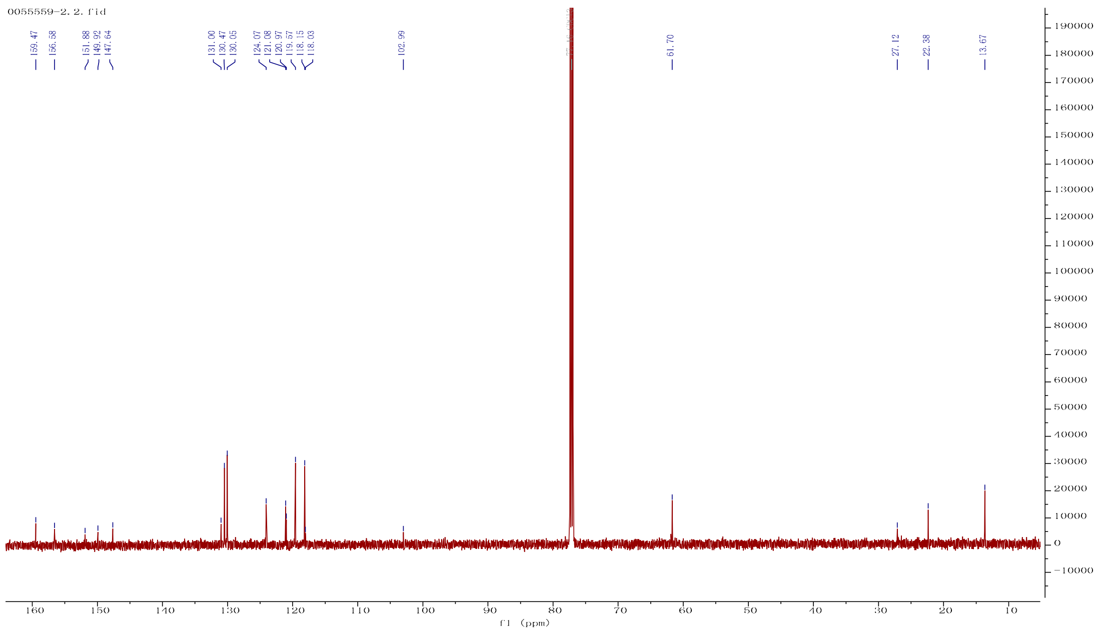

MS spectra

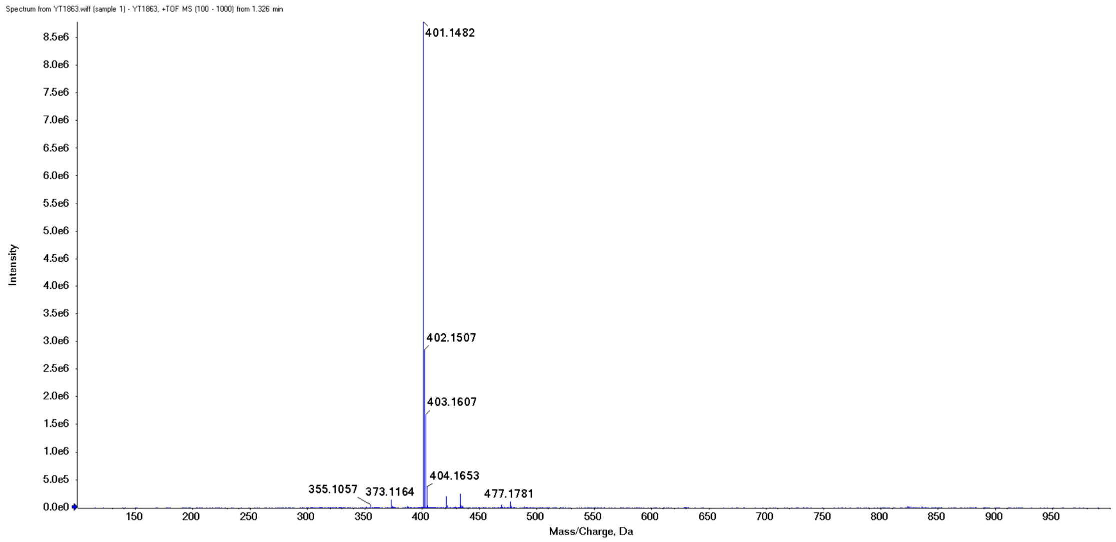

Formula Calculator Results

Measured m/z	Cal m/z	Error(mmu)	Error(ppm)	Ion Formula	Ion
401.14815	401.14958	-1.4	-3.6	$C_{24}H_{21}N_2O_4$	$[M+H]^+$

2.6 ^{1}H NMR, ^{13}C NMR and HRMS of K1

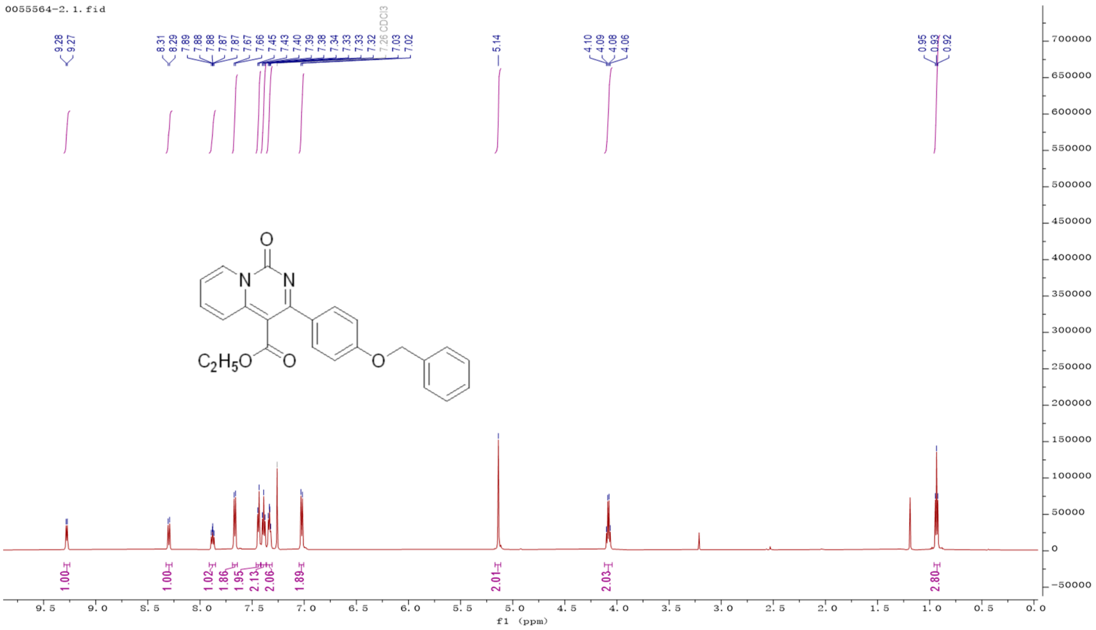

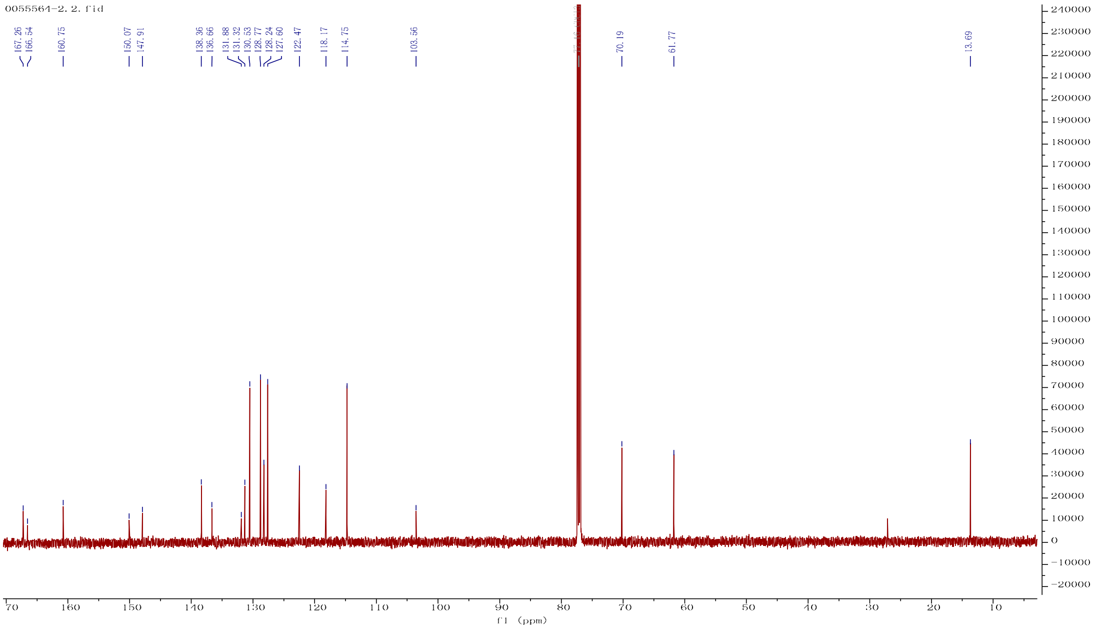

MS spectra

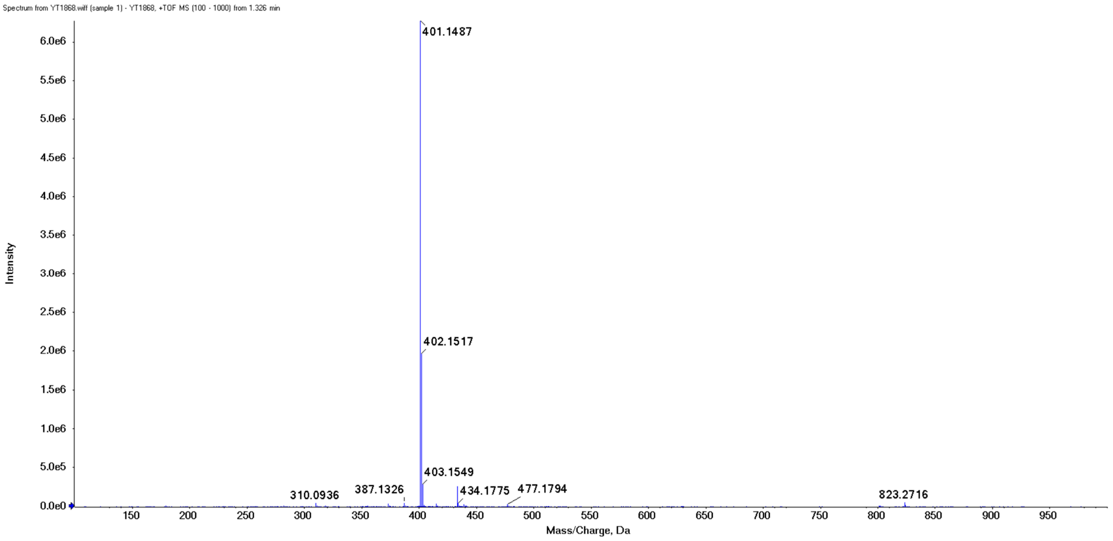

Formula Calculator Results

Measured m/z	Cal m/z	Error(mmu)	Error(ppm)	Ion Formula	Ion
401.14869	401.14958	-0.9	-2.2	$C_{24}H_{21}N_2O_4$	$[M+H]^+$

2.7 ^{1}H NMR and ^{13}C NMR of K2

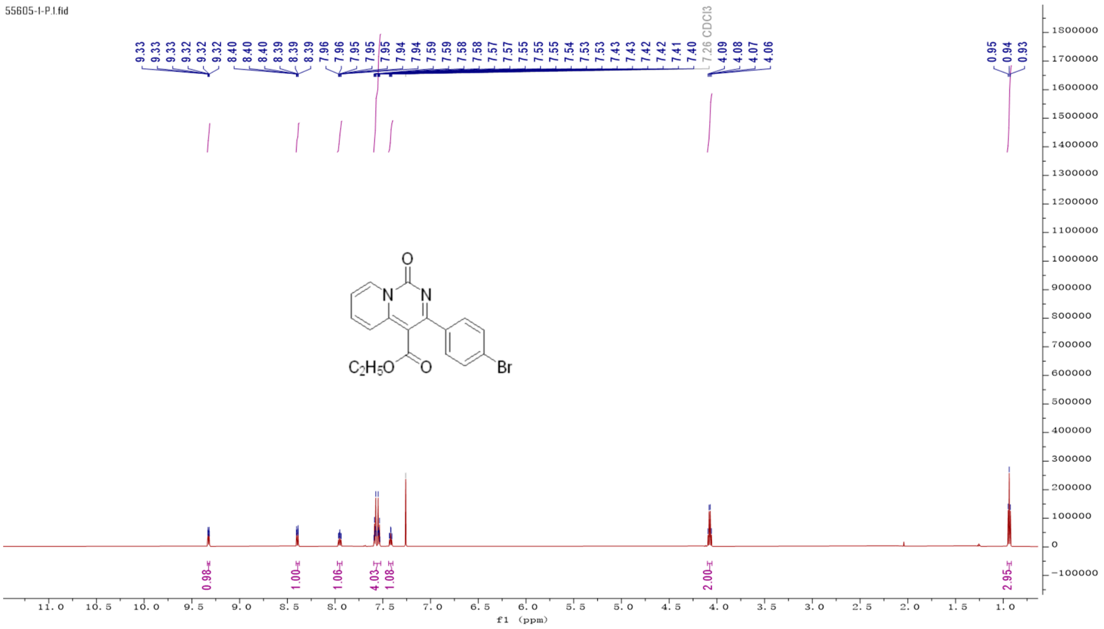

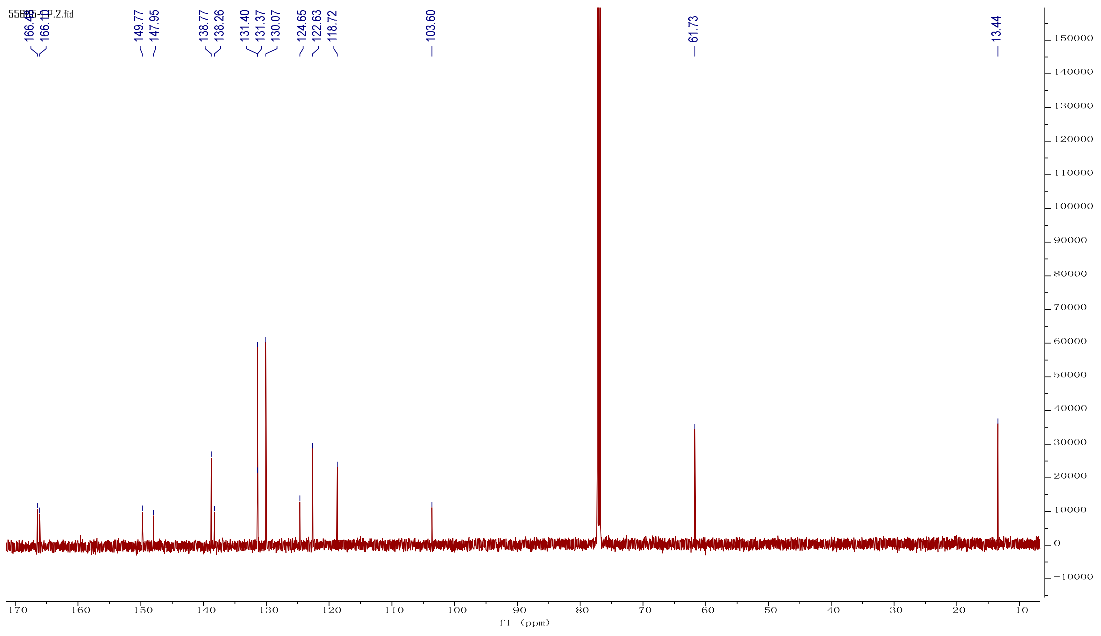

2.8 ^{1}H NMR, ^{13}C NMR and HRMS of K3

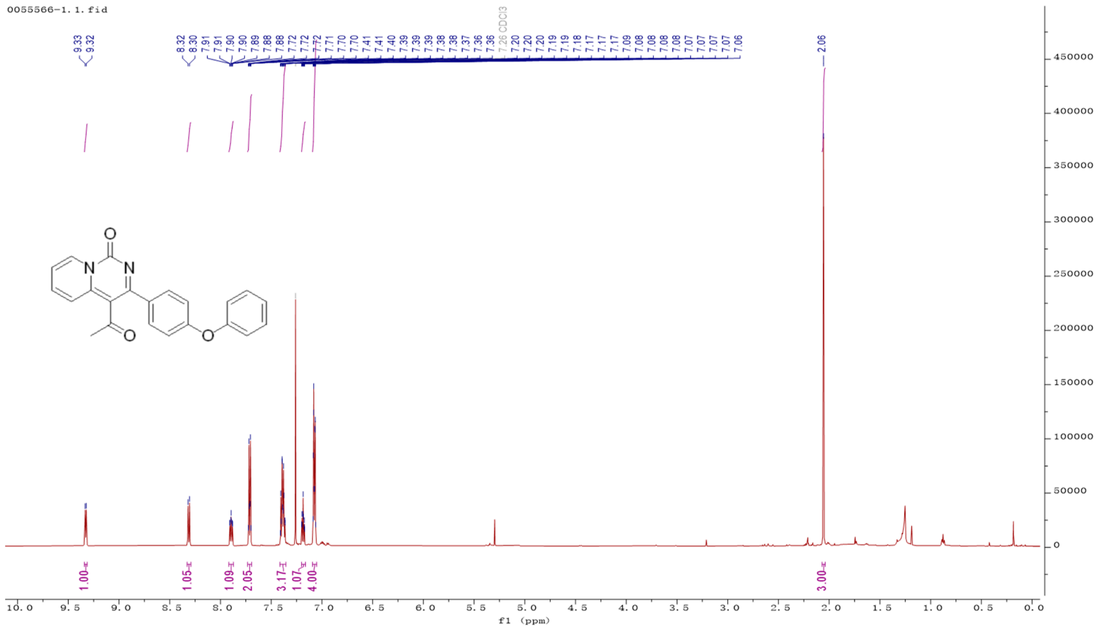

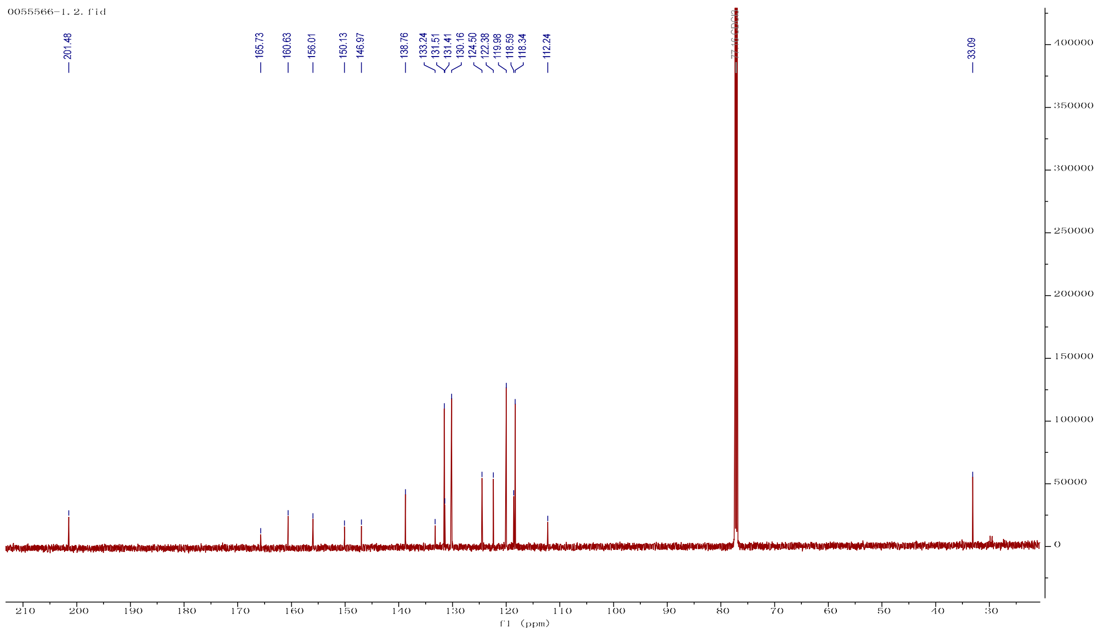

MS spectra

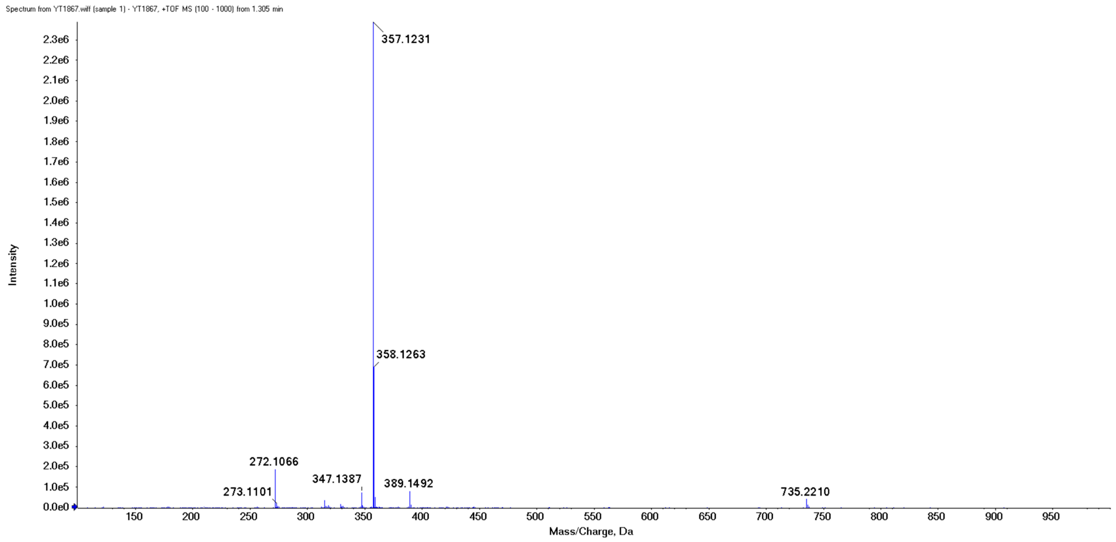

Formula Calculator Results

Measured m/z	Cal m/z	Error(mmu)	Error(ppm)	Ion Formula	Ion
357.12315	357.12337	-0.2	-0.6	$C_{22}H_{17}N_2O_3$	$[M+H]^+$

2.9 ^{1}H NMR, ^{13}C NMR and HRMS of M0

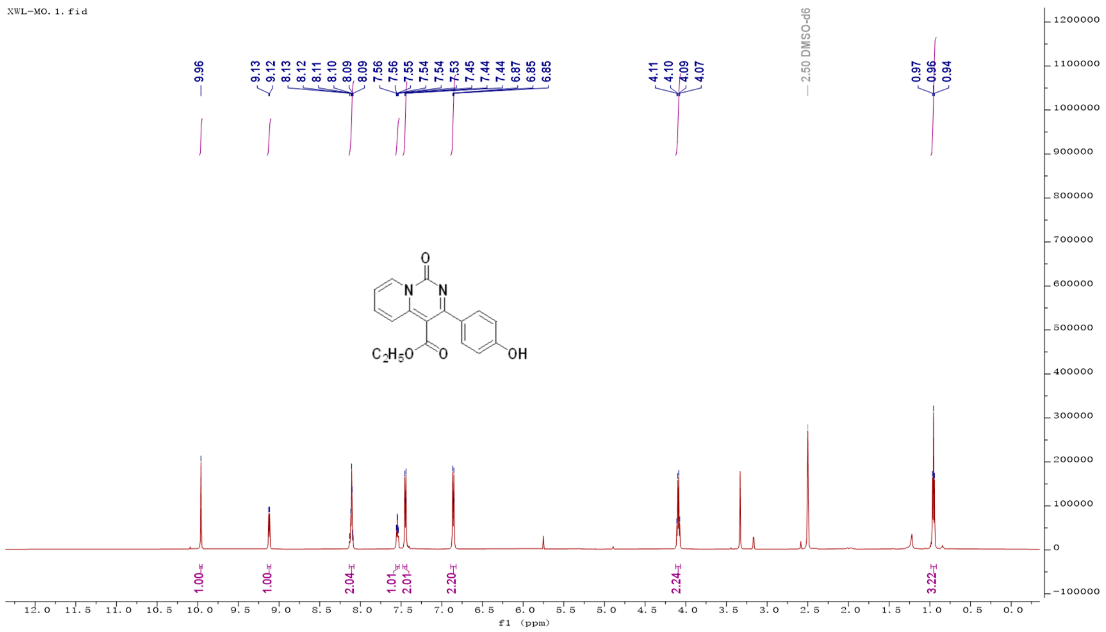
XWL-MO.1.fid
9.96
9.13
9.12
8.13
8.12
8.11
8.10
8.09
8.09
7.56
7.55
7.54
7.54
7.53
7.45
7.44
7.44
6.87
6.85
6.85
4.11
4.10
4.09
4.07
2.50 DMSO-d6
0.97
0.96
0.94
O
N
N
C2H5O
O
OH
1.00
1.00
2.04
1.01
2.01
2.20
2.24
3.22
f1 (ppm)

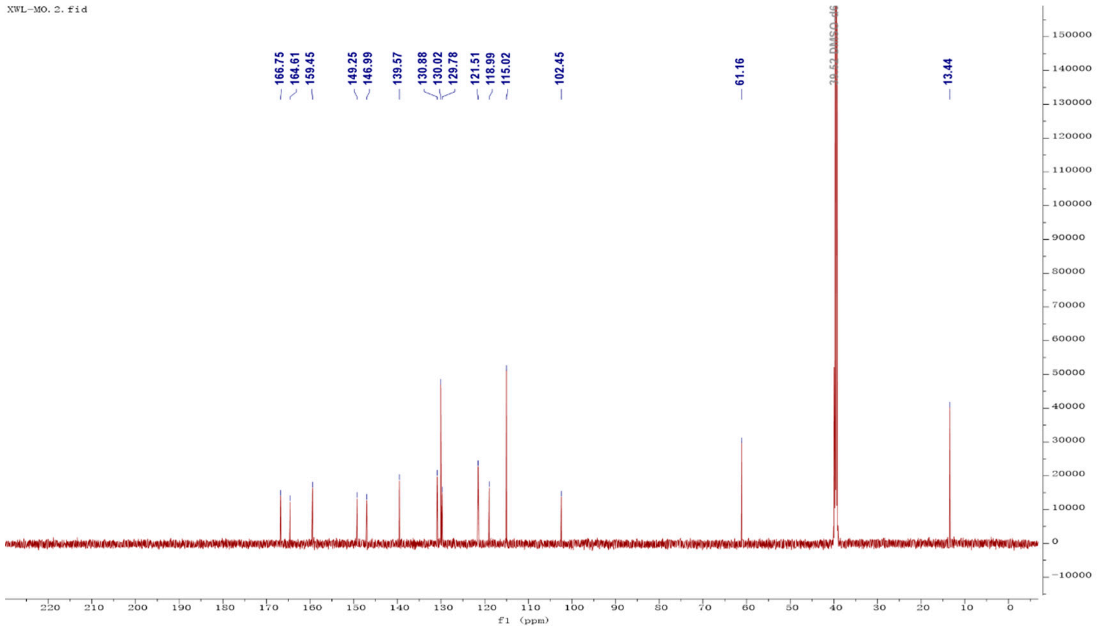
XWL-MO.2.fid
166.75
164.61
159.45
149.25
146.99
139.57
130.88
130.02
129.78
121.51
118.99
115.02
102.45
61.16
13.44
f1 (ppm)

MS spectra

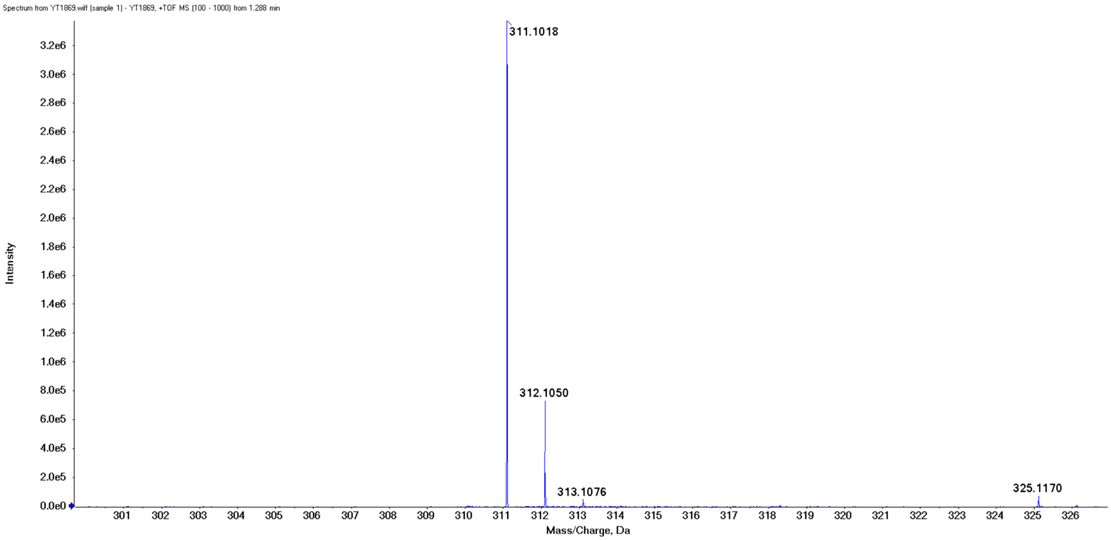

Formula Calculator Results

Measured m/z	Cal m/z	Error(mmu)	Error(ppm)	Ion Formula	Ion
311.10177	311.10263	-0.9	-2.8	$C_{17}H_{15}N_2O_4$	$[M+H]^+$

2.10 ^{1}H NMR, ^{13}C NMR and HRMS of M1

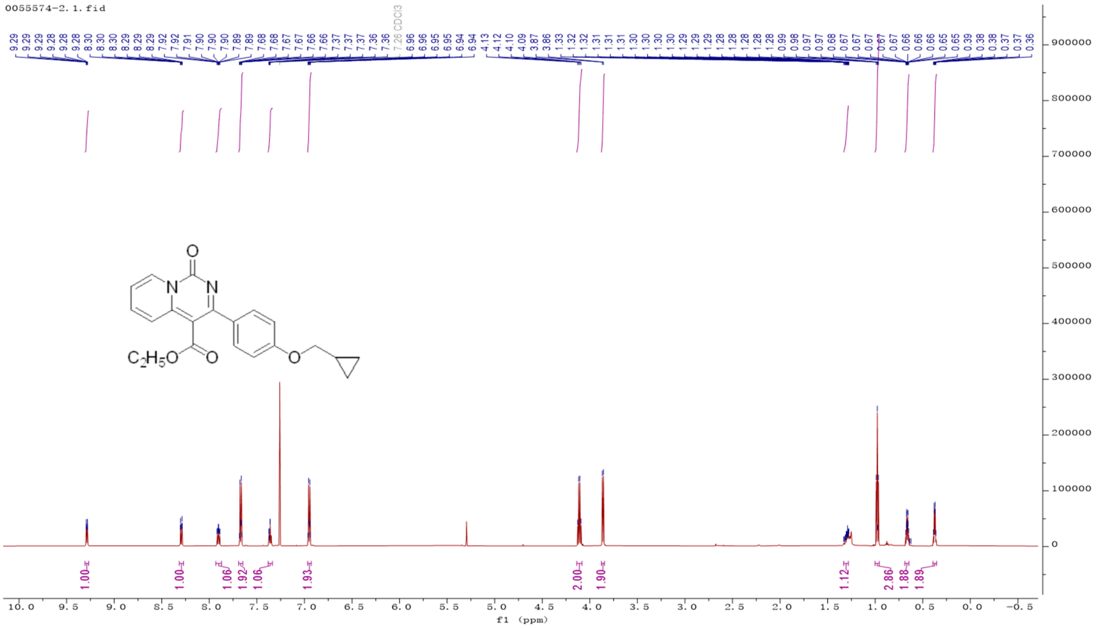

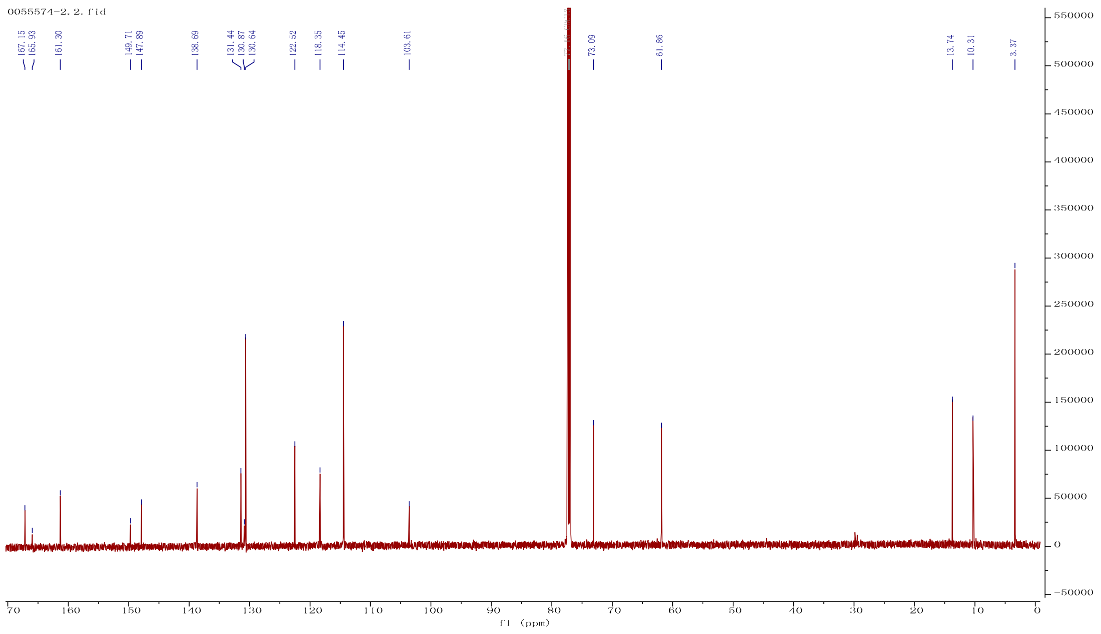

MS spectra

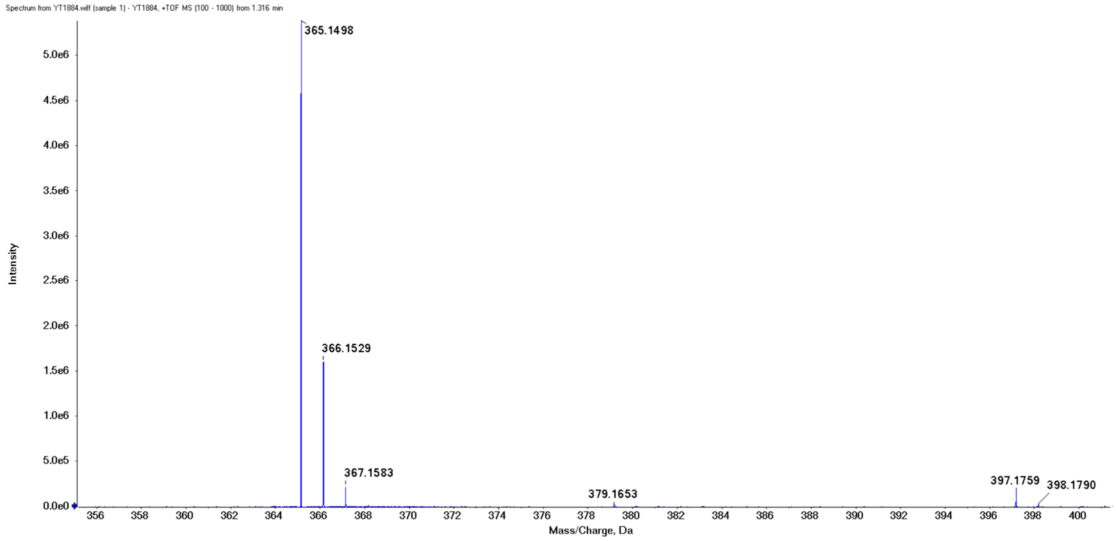

Formula Calculator Results

Measured m/z	Cal m/z	Error(mmu)	Error(ppm)	Ion Formula	Ion
**365.14981**	**365.14958**	**0.2**	**0.6**	$C_{21}H_{21}N_2O_4$	$[M+H]^+$

2.11 ^{1}H NMR, ^{13}C NMR and HRMS of M2

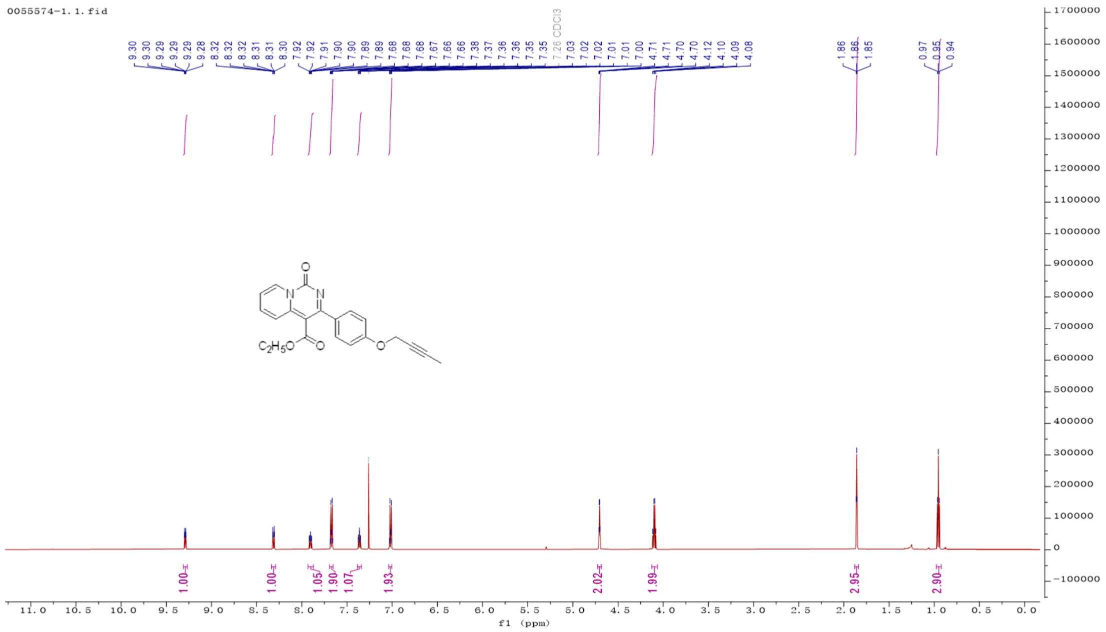
0055574-1.1.fid
9.30 9.30 9.29 9.29 9.28 8.32 8.32 8.32 8.31 8.31 8.30 7.92 7.91 7.90 7.90 7.89 7.68 7.68 7.67 7.66 7.66 7.38 7.37 7.36 7.36 7.35 7.35 7.26 CDCl3 7.03 7.02 7.02 7.01 7.01 7.00 4.71 4.71 4.70 4.70 4.12 4.10 4.09 4.08 1.86 1.86 1.85 0.97 0.95 0.94
C_2H_5O
1.00 1.00 1.05 1.90 1.07 1.93 2.02 1.99 2.95 2.90
f1 (ppm)

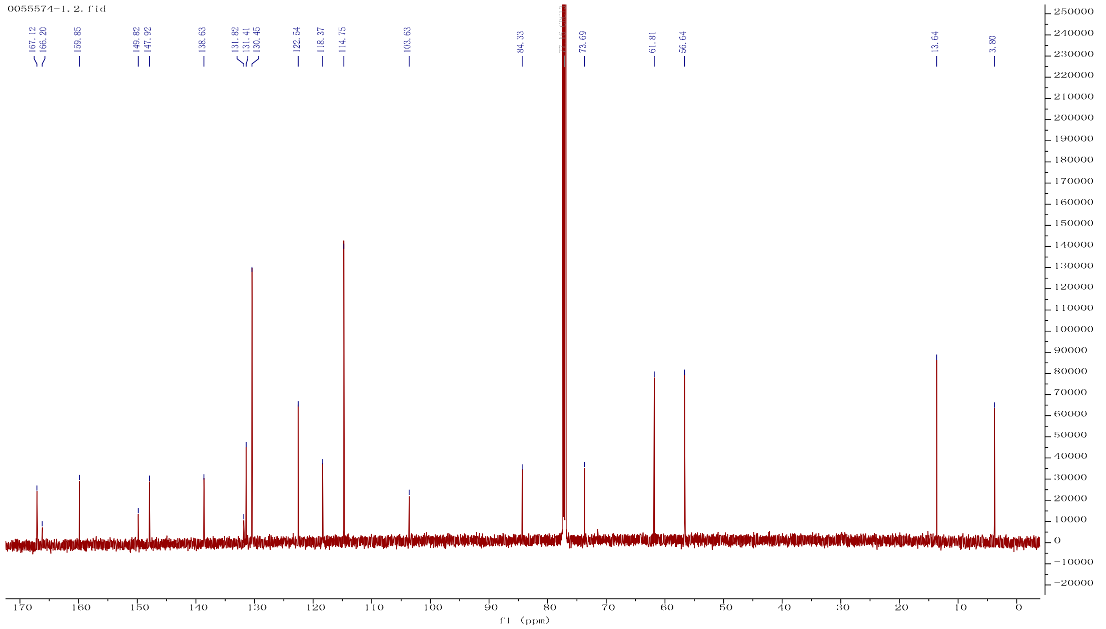
0055574-1.2.fid
167.12 166.20 159.85 149.82 147.92 138.63 131.82 131.41 130.45 122.54 118.37 114.75 103.63 84.33 73.69 61.81 56.64 13.64 3.80
f1 (ppm)

MS spectra

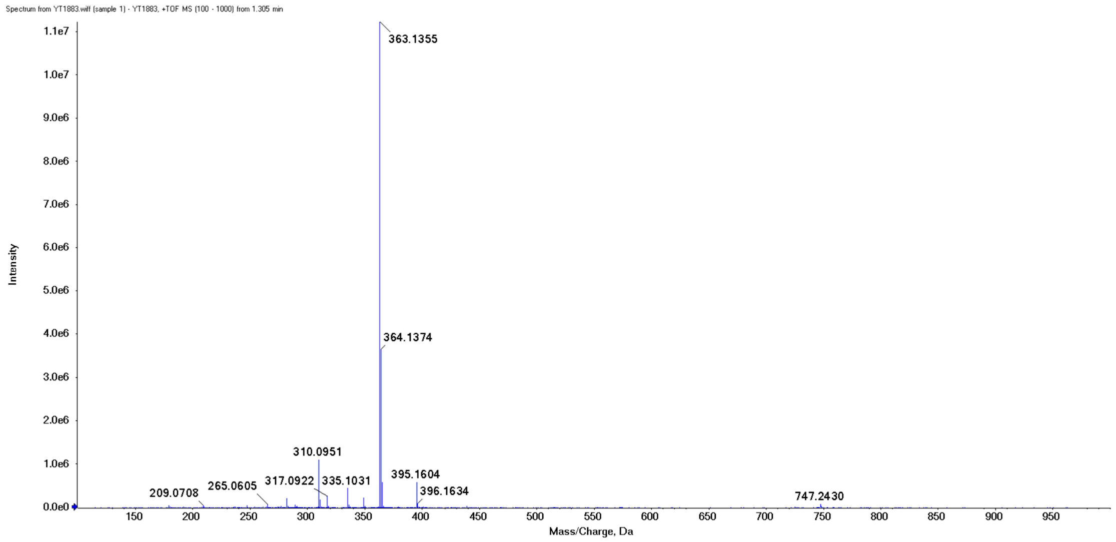

Formula Calculator Results

Measured m/z	Cal m/z	Error(mmu)	Error(ppm)	Ion Formula	Ion
363.13549	363.13393	1.6	4.3	$C_{21}H_{19}N_2O_4$	$[M+H]^+$

2.12 ^{1}H NMR, ^{13}C NMR and HRMS of M3

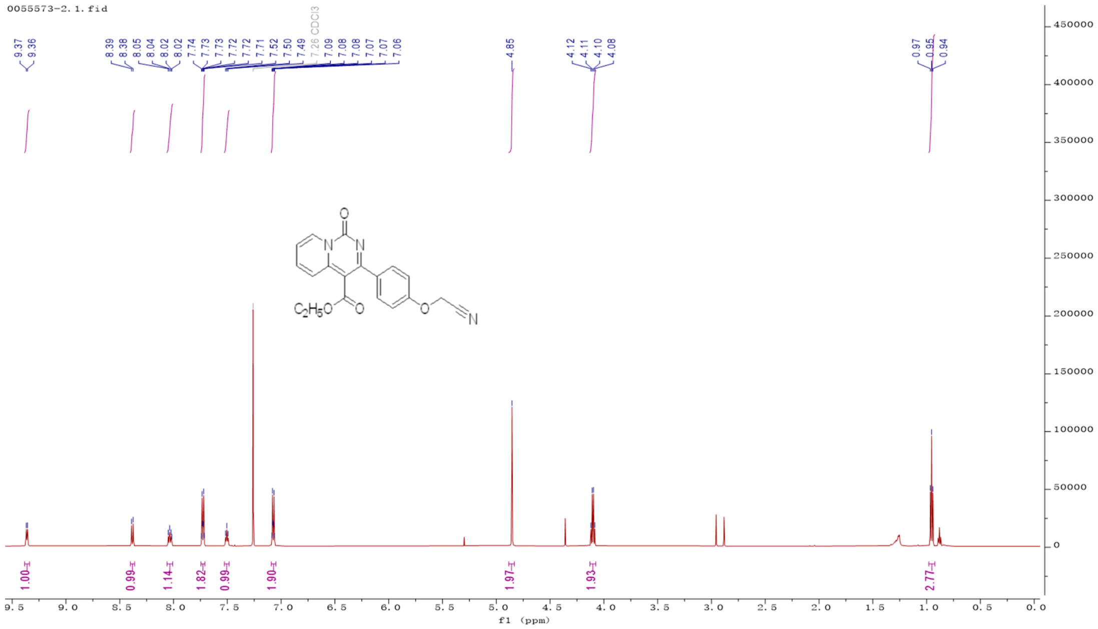

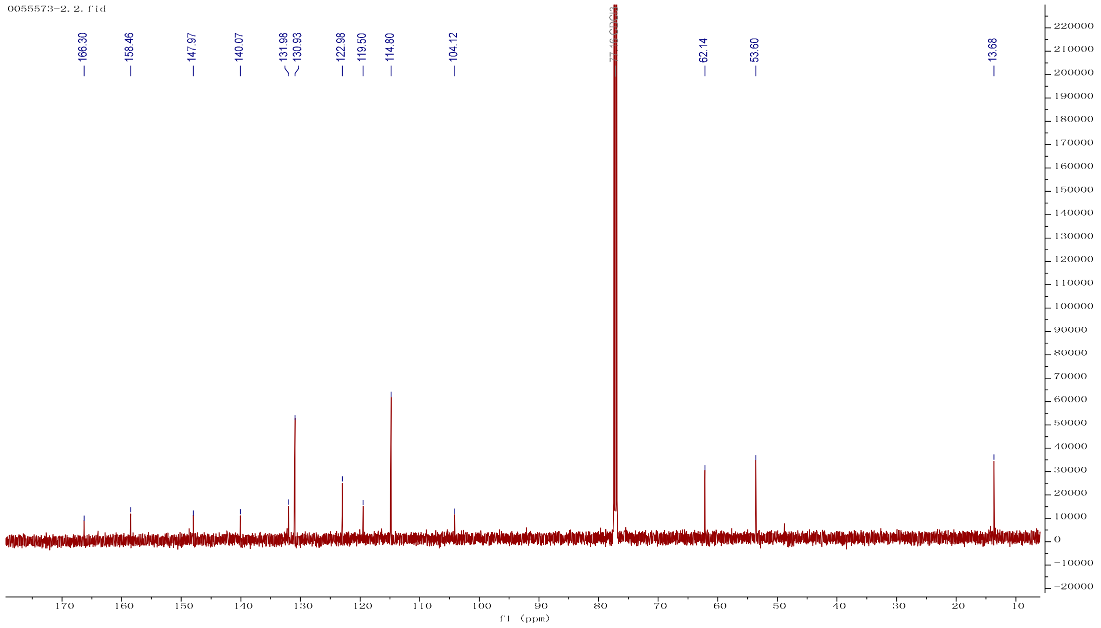

MS spectra

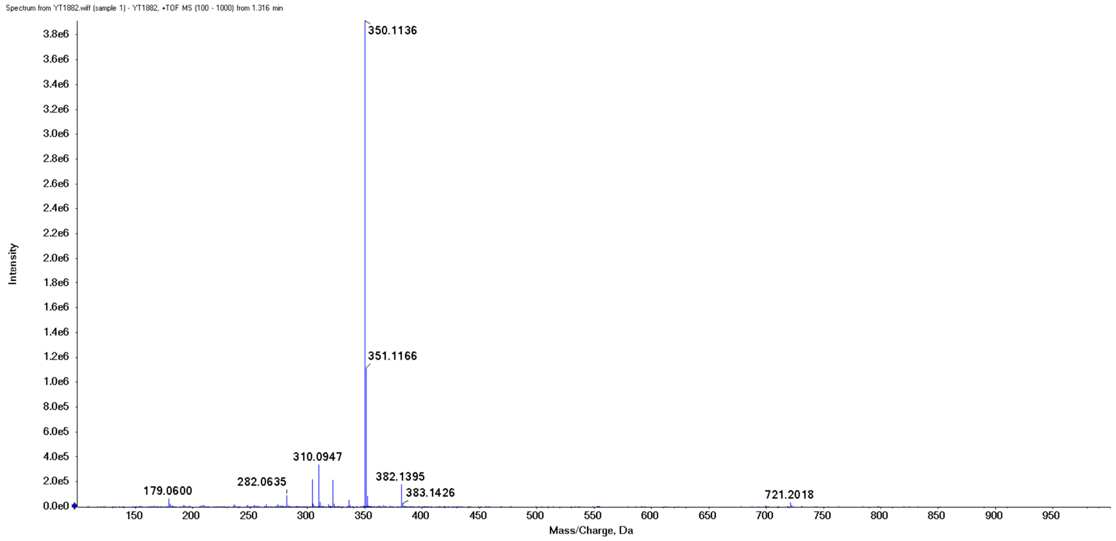

Formula Calculator Results

Measured m/z	Cal m/z	Error(mmu)	Error(ppm)	Ion Formula	Ion
350.11357	350.11353	0.0	0.1	$C_{19}H_{16}N_3O_4$	$[M+H]^+$

2.13 ^{1}H NMR, ^{13}C NMR and HRMS of M4

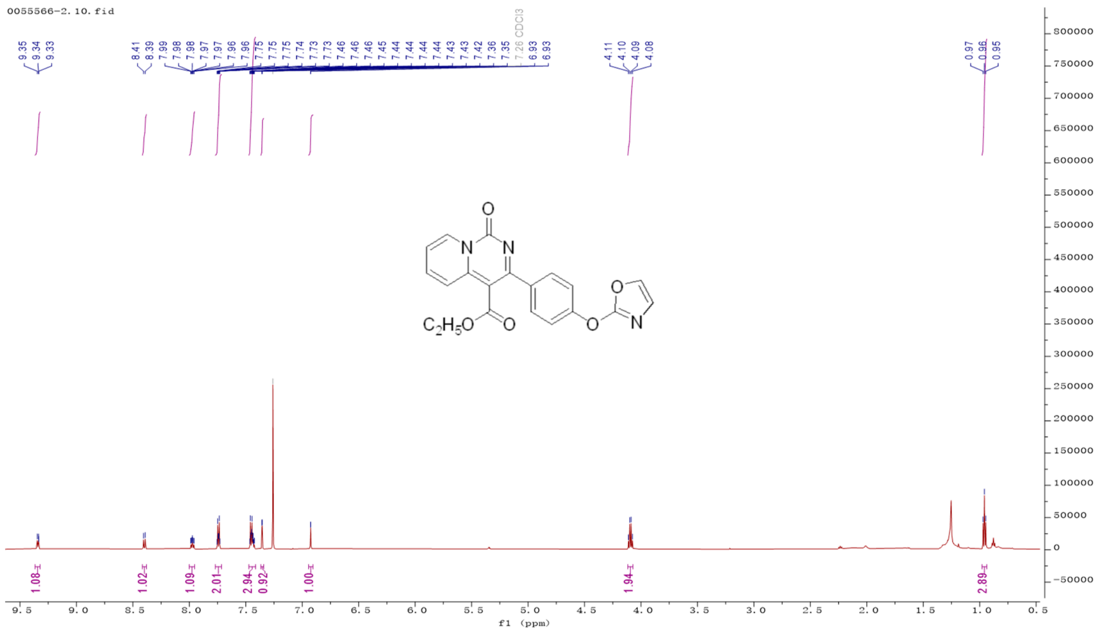

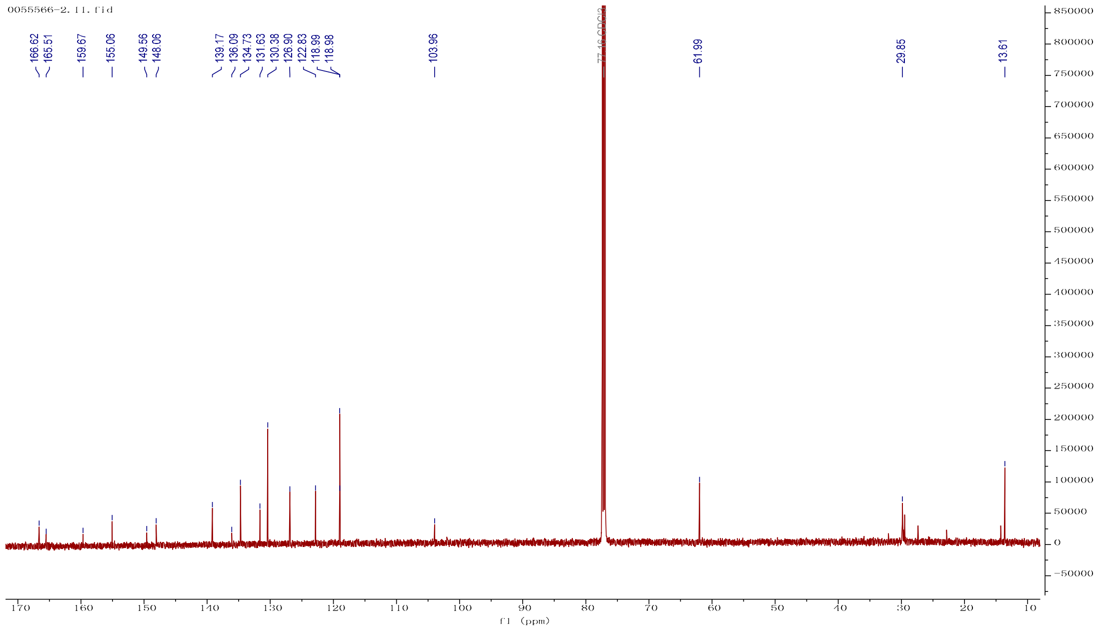

MS spectra

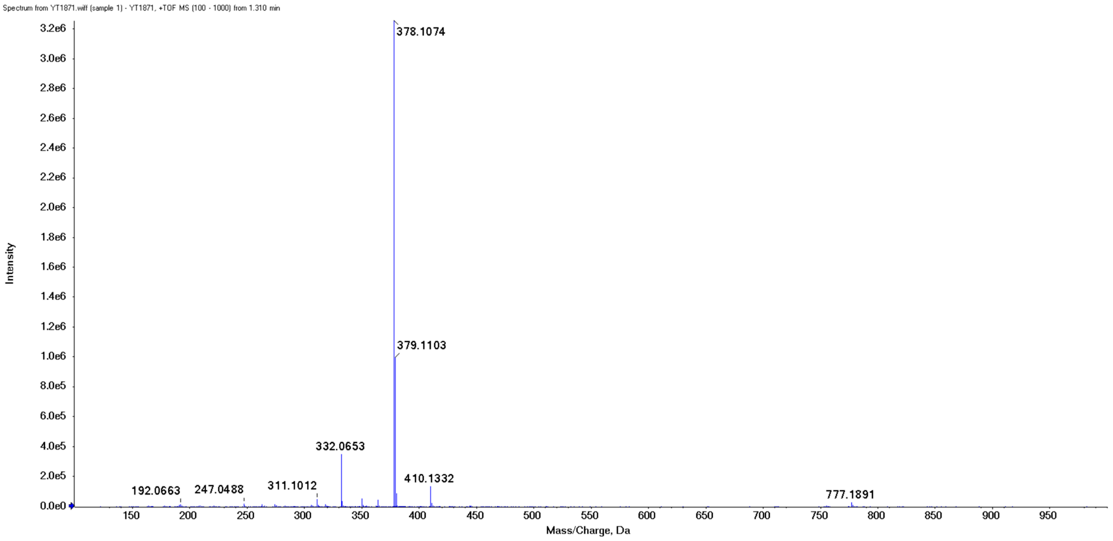

Formula Calculator Results

Measured m/z	Cal m/z	Error(mmu)	Error(ppm)	Ion Formula	Ion
378.10741	378.10845	-1.0	-2.8	$C_{20}H_{16}N_3O_5$	$[M+H]^+$

2.14 ^{1}H NMR, ^{13}C NMR and HRMS of M5

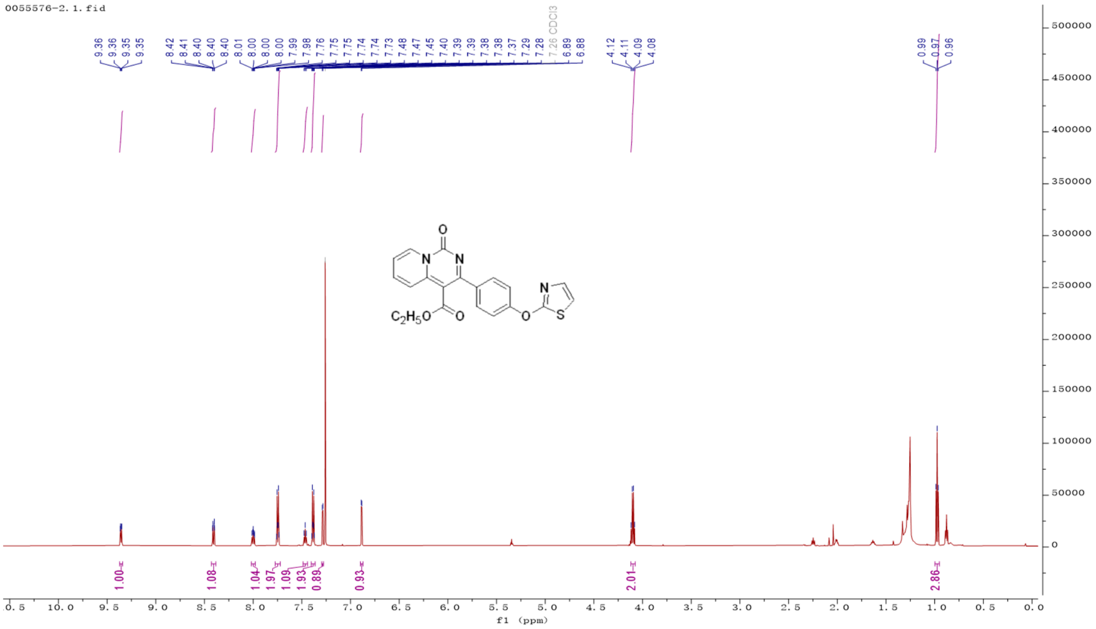

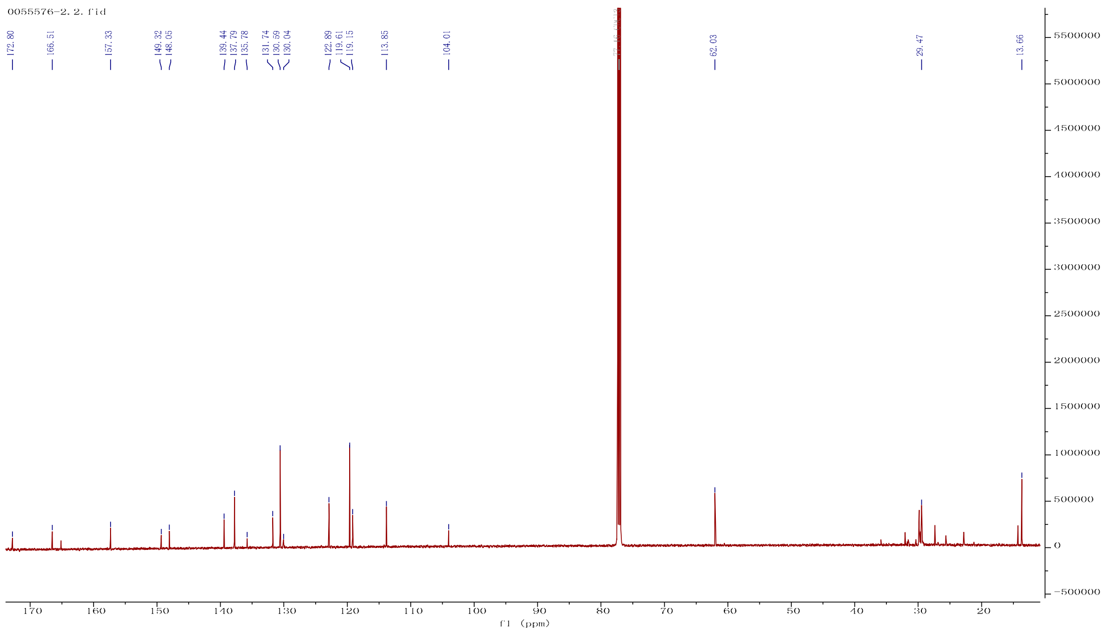

MS spectra

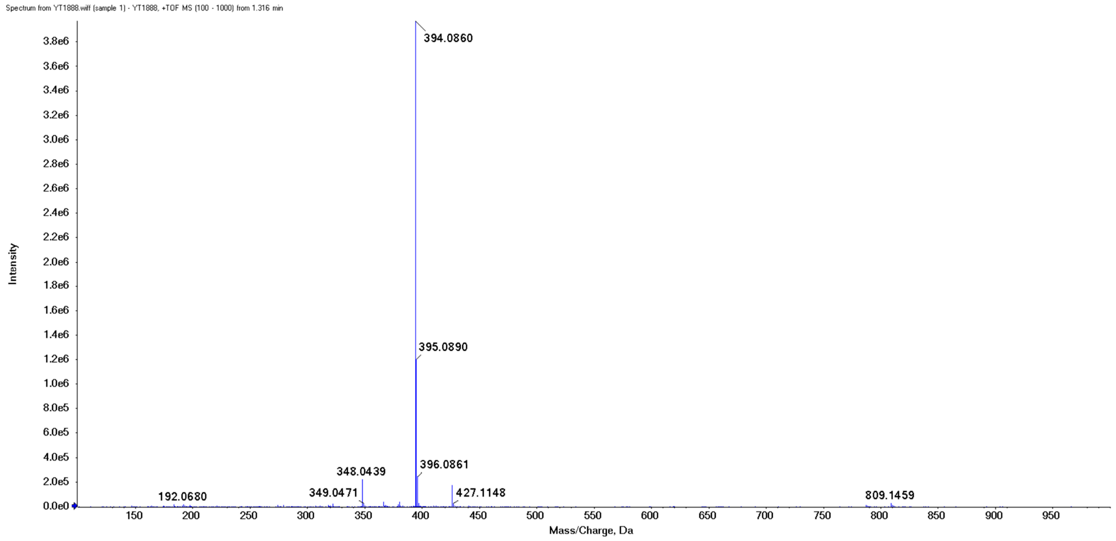

Formula Calculator Results

Measured m/z	Cal m/z	Error(mmu)	Error(ppm)	Ion Formula	Ion
394.08599	394.08560	0.4	1.0	$C_{20}H_{16}N_3O_4$	$[M+H]^+$

2.15 ^{1}H NMR, ^{13}C NMR and HRMS of M6

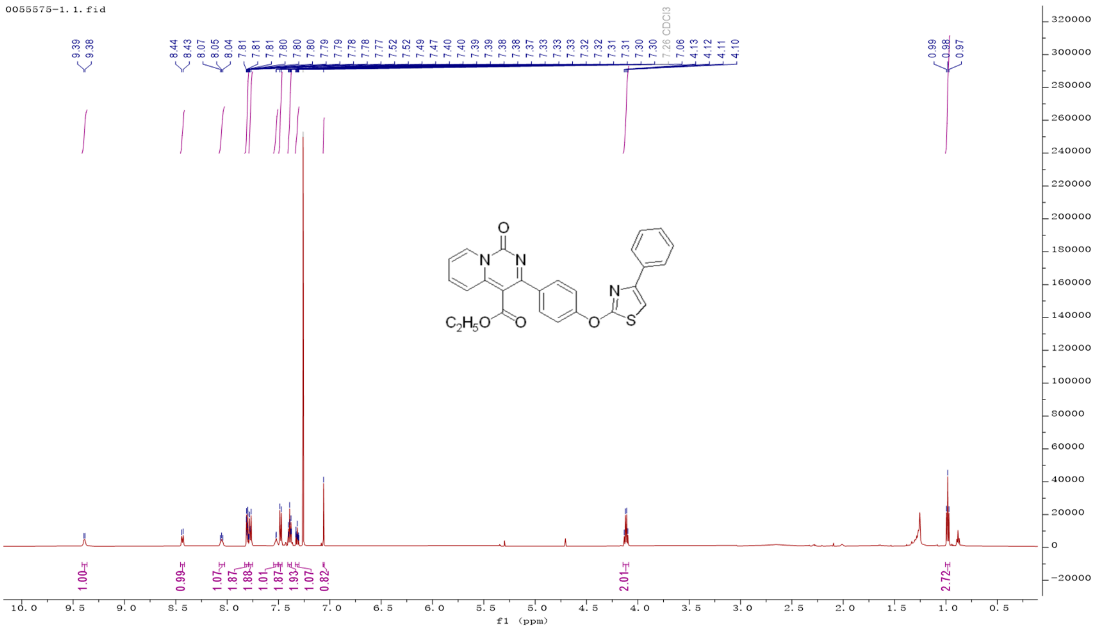

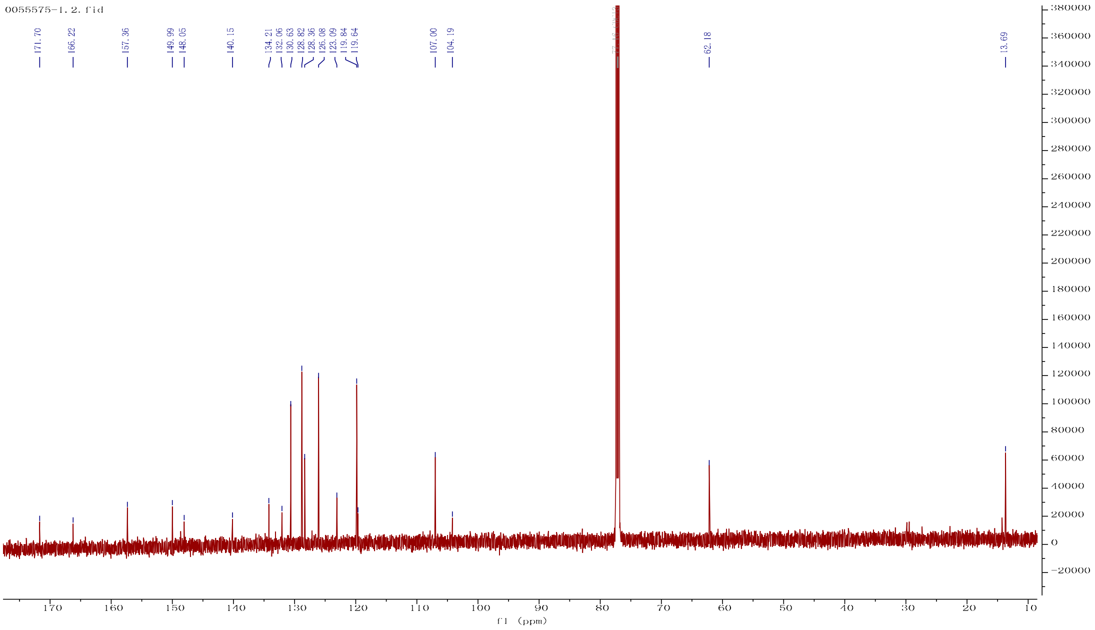

MS spectra

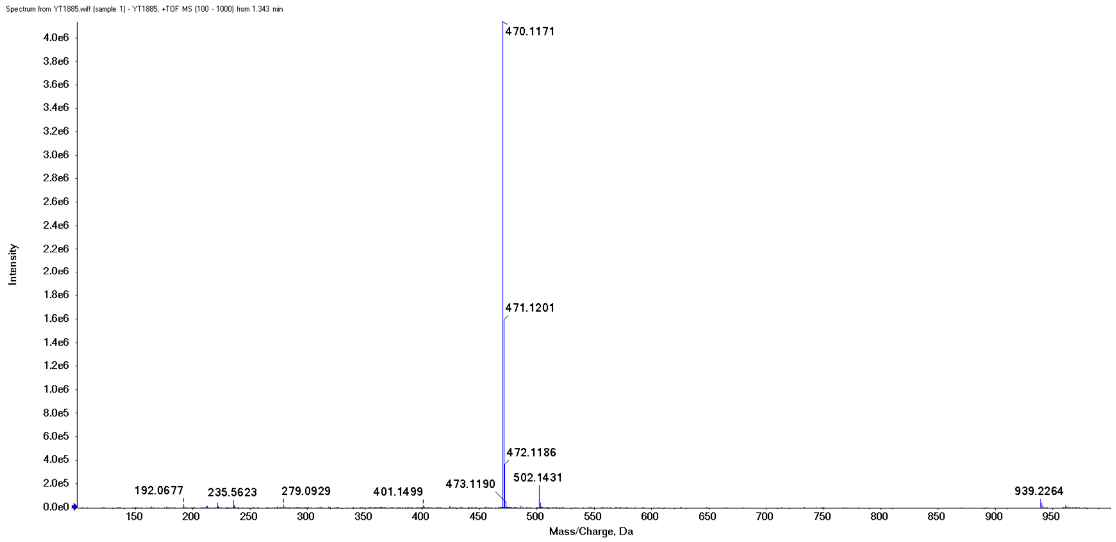

Formula Calculator Results

Measured m/z	Cal m/z	Error(mmu)	Error(ppm)	Ion Formula	Ion
470.11711	470.11690	0.2	0.4	$C_{26}H_{20}N_3O_4S$	$[M+H]^+$

2.16 ^{1}H NMR, ^{13}C NMR and HRMS of M7

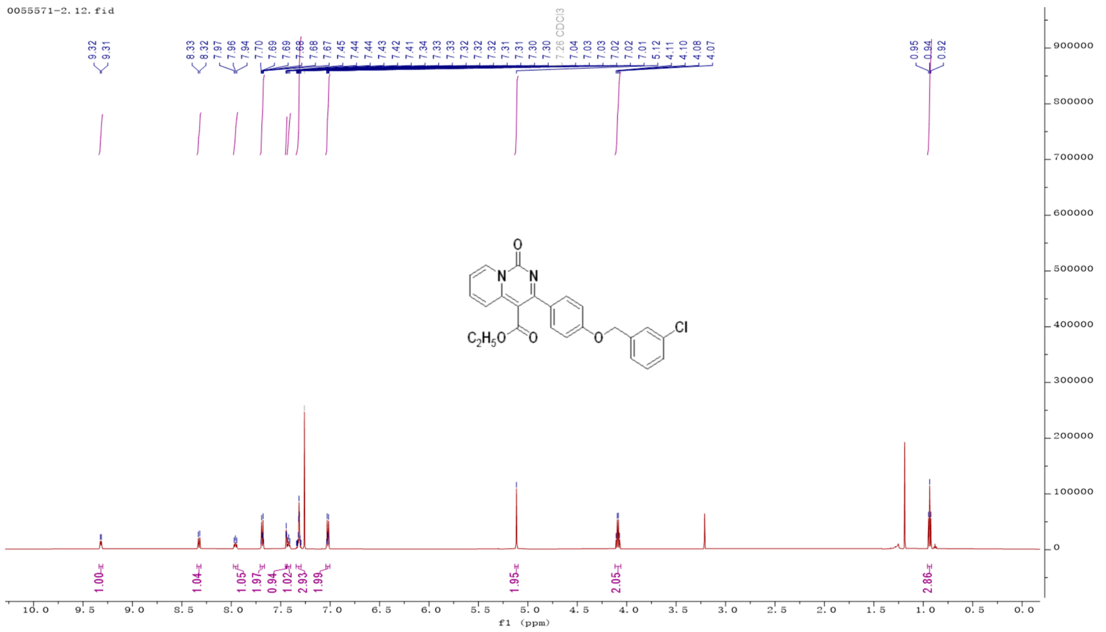

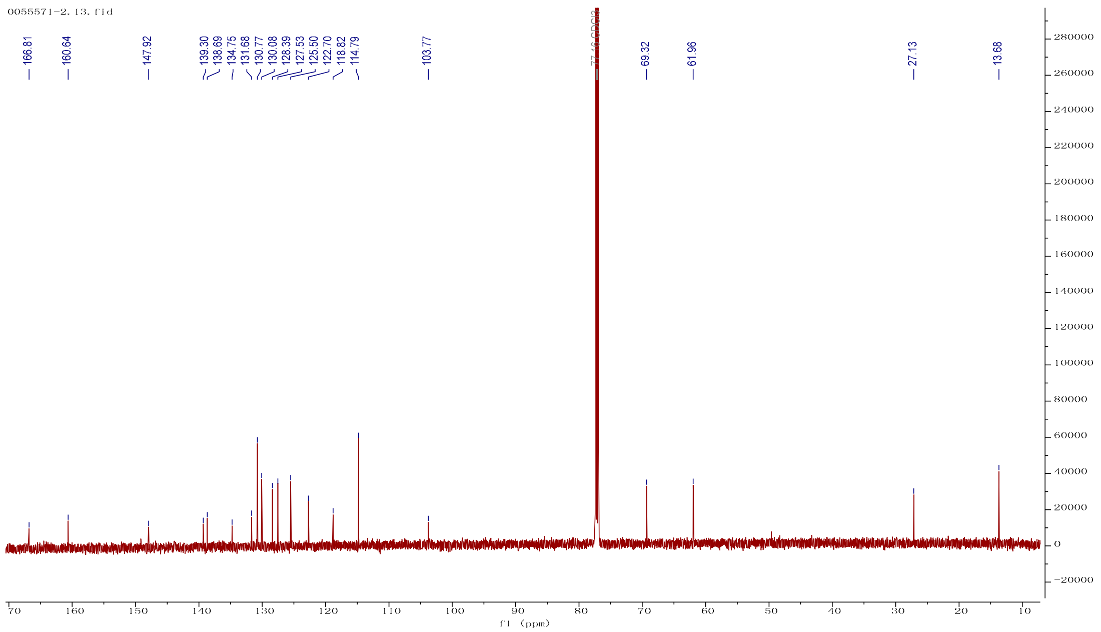

MS spectra

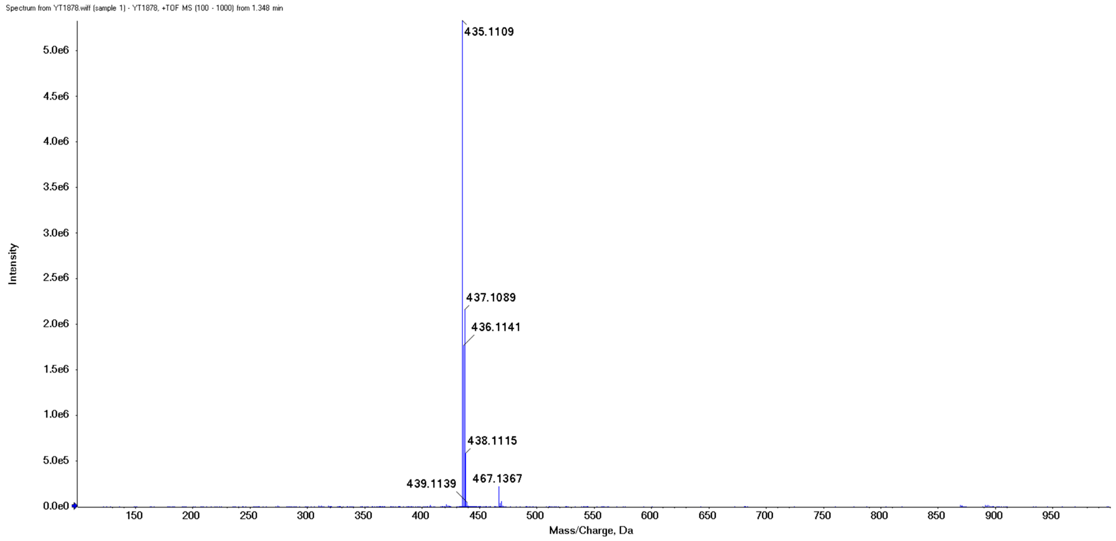

Formula Calculator Results

Measured m/z	Cal m/z	Error(mmu)	Error(ppm)	Ion Formula	Ion
**435.11094**	**435.11061**	**0.3**	**0.8**	$C_{24}H_{20}ClN_2O_4$	**[M+H]$^+$**

2.17 ^{1}H NMR, ^{13}C NMR and HRMS of M8

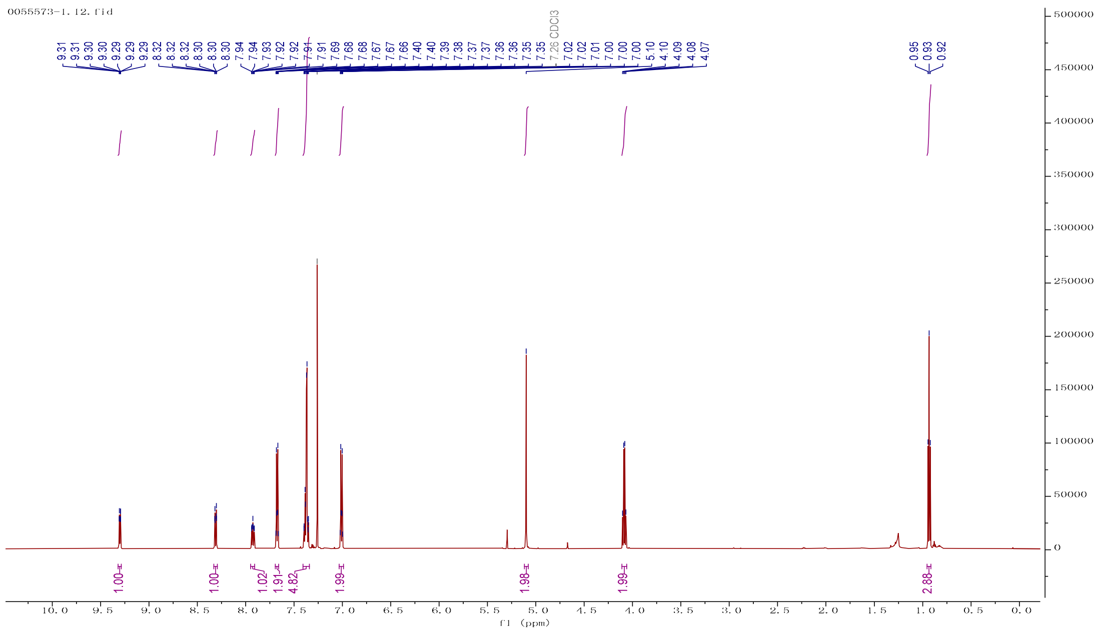
0055573-1.12.fid
9.31 9.31 9.30 9.30 9.29 9.29 9.29 8.32 8.32 8.32 8.30 8.30 8.30 7.94 7.94 7.93 7.92 7.92 7.91 7.91 7.69 7.68 7.68 7.67 7.67 7.66 7.40 7.40 7.39 7.38 7.37 7.37 7.36 7.36 7.35 7.35 7.26 CDCl3 7.02 7.02 7.01 7.00 7.00 5.10 4.10 4.09 4.08 4.07
0.95 0.93 0.92
1.00 1.00 1.02 1.91 4.82 1.99 1.98 1.99 2.88
f1 (ppm)

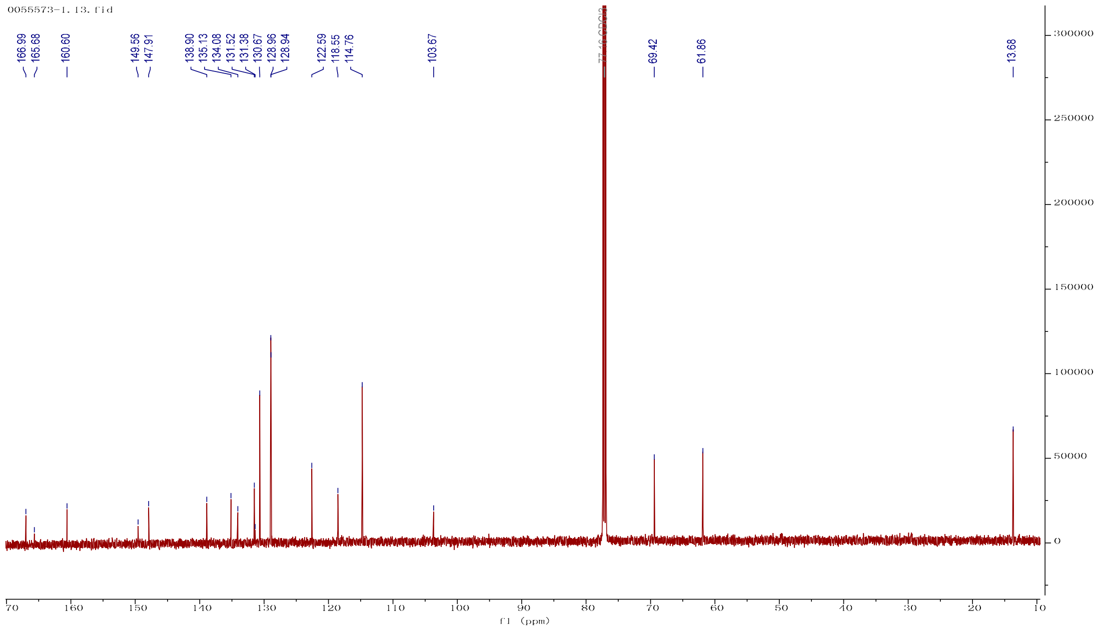
0055573-1.13.fid
166.99 165.68 160.60 149.56 147.91 138.90 135.13 134.08 131.52 131.38 130.67 128.96 128.94 122.59 118.55 114.76 103.67
69.42 61.86 13.68
f1 (ppm)

MS spectra

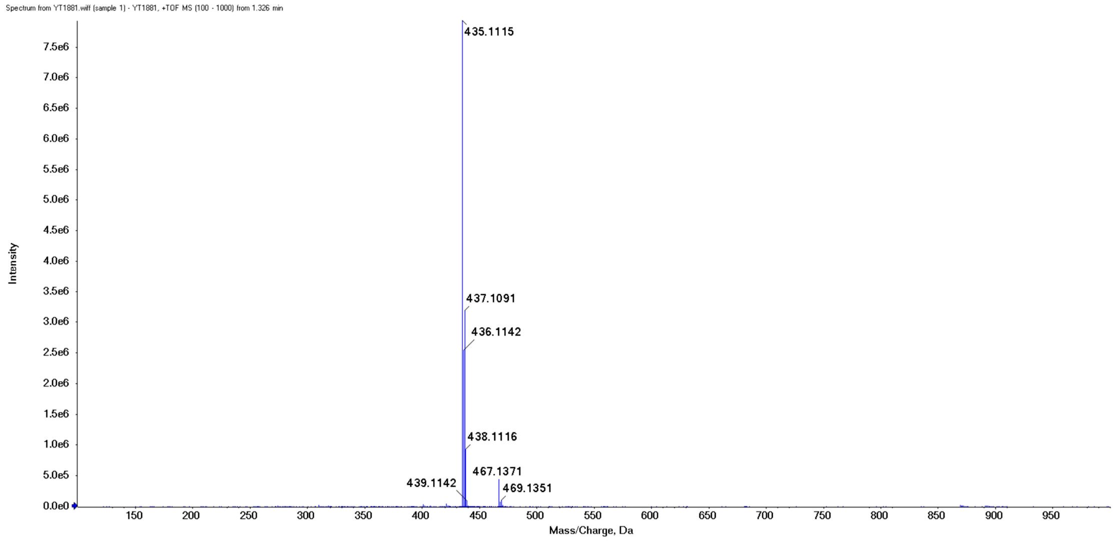

Formula Calculator Results

Measured m/z	Cal m/z	Error(mmu)	Error(ppm)	Ion Formula	Ion
435.11152	435.11061	0.9	2.1	$C_{24}H_{20}ClN_2O_4$	$[M+H]^+$

2.18 ^{1}H NMR, ^{13}C NMR and HRMS of M9

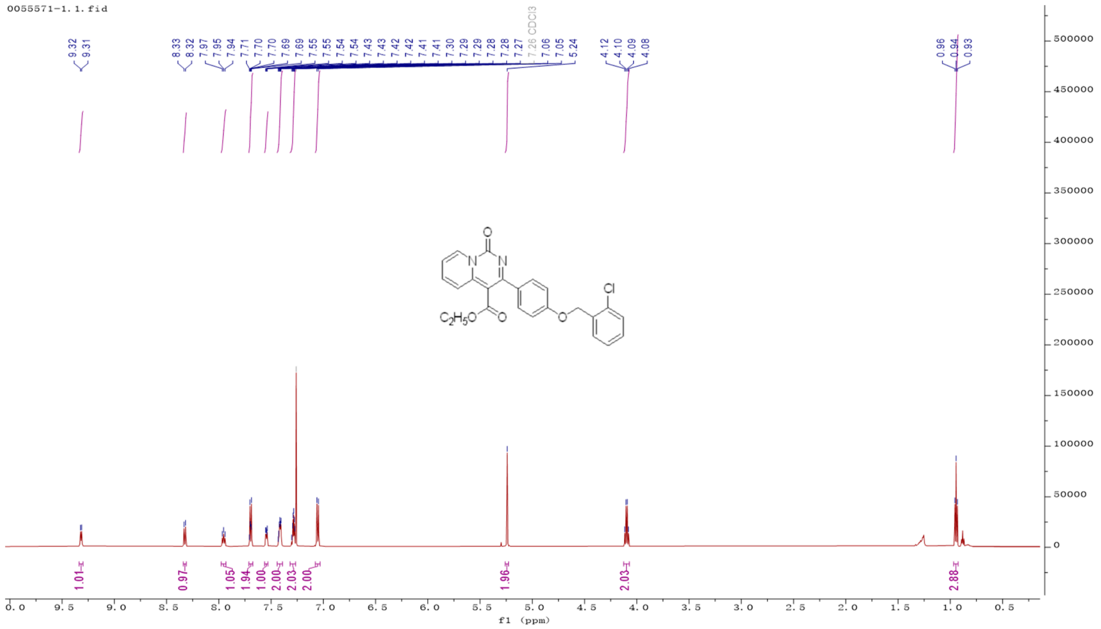

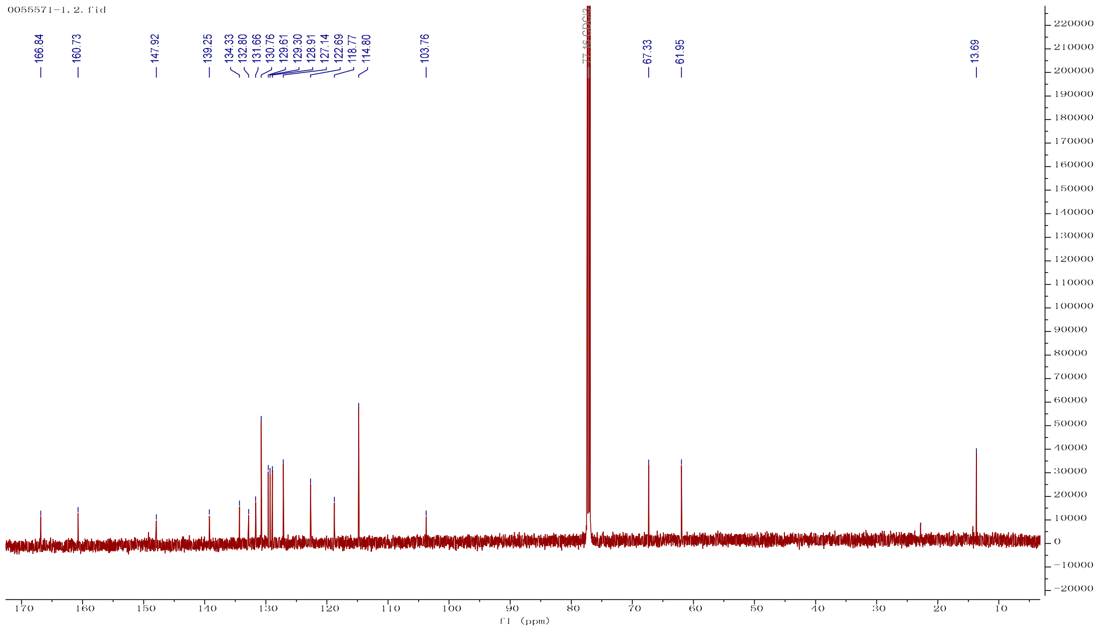

MS spectra

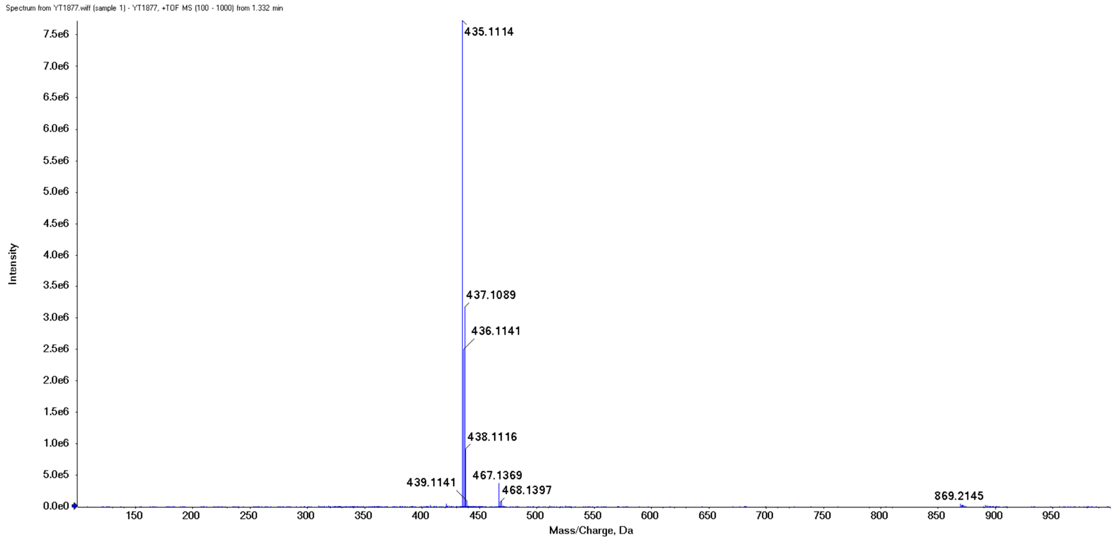

Formula Calculator Results

Measured m/z	Cal m/z	Error(mmu)	Error(ppm)	Ion Formula	Ion
435.11136	435.11061	0.7	1.7	$C_{24}H_{20}ClN_2O_4$	$[M+H]^+$

2.19 ^{1}H NMR, ^{13}C NMR and HRMS of M10

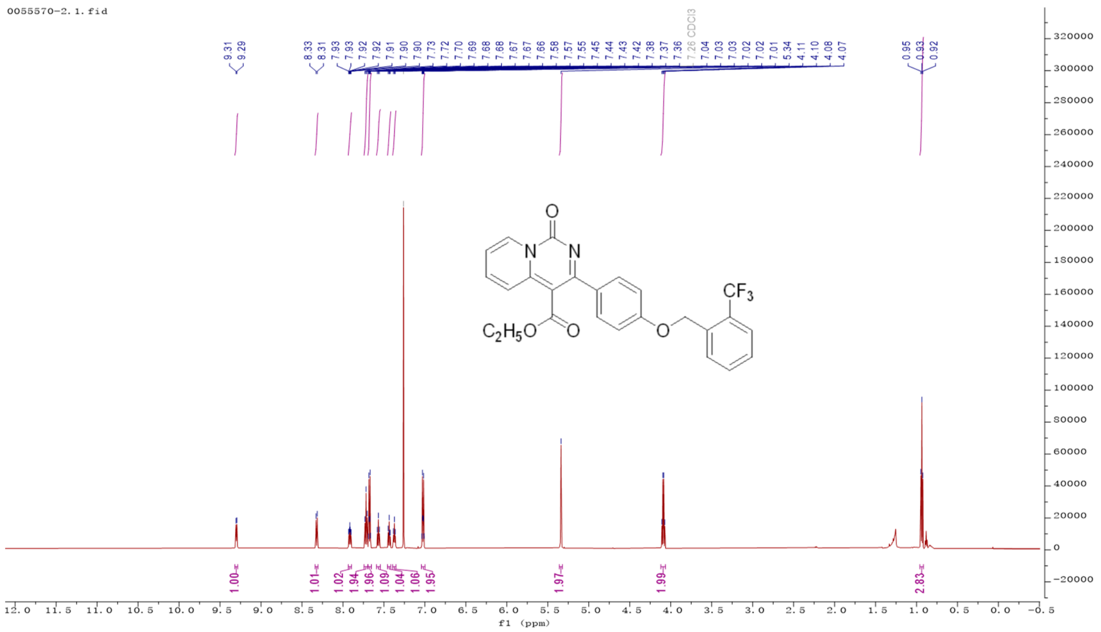

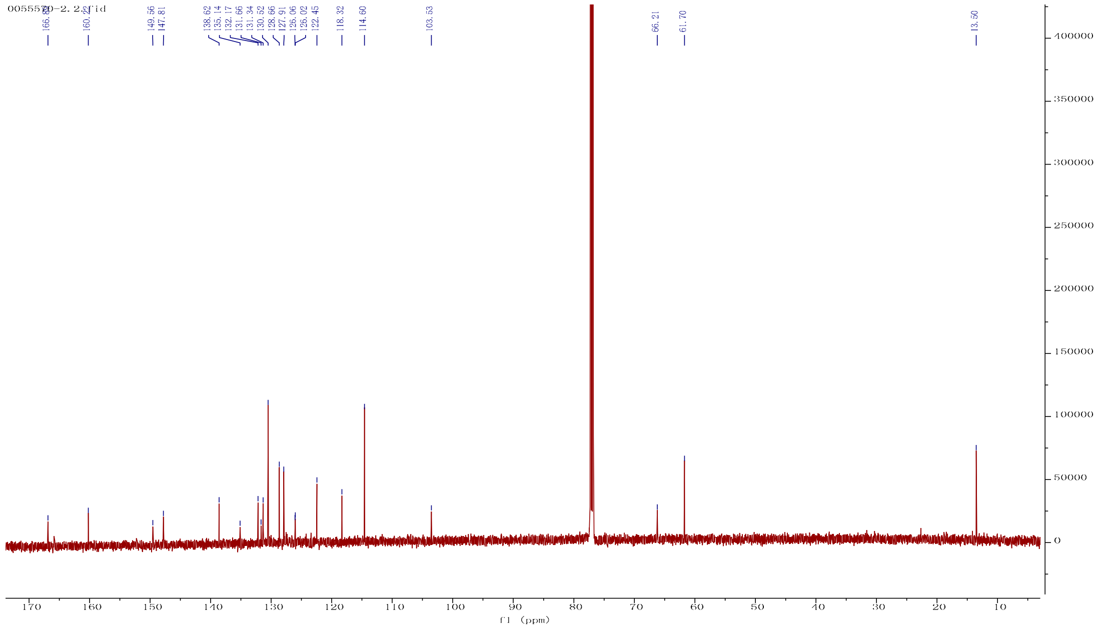

MS spectra

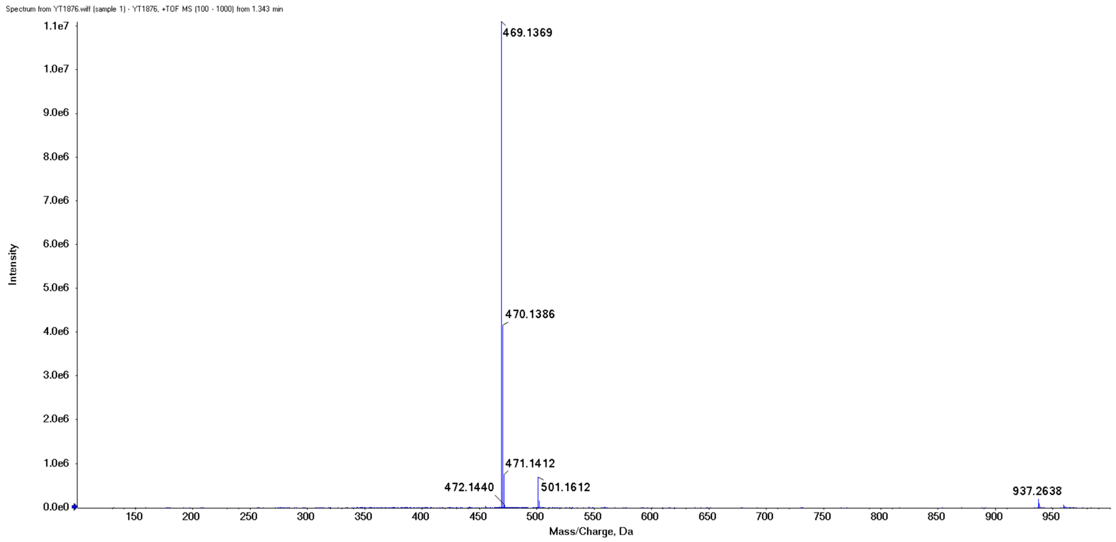

Formula Calculator Results

Measured m/z	Cal m/z	Error(mmu)	Error(ppm)	Ion Formula	Ion
469.13685	469.13697	-0.1	-0.3	$C_{25}H_{20}F_3N_2O_4$	$[M+H]^+$

2.20 ^{1}H NMR, ^{13}C NMR and HRMS of M11

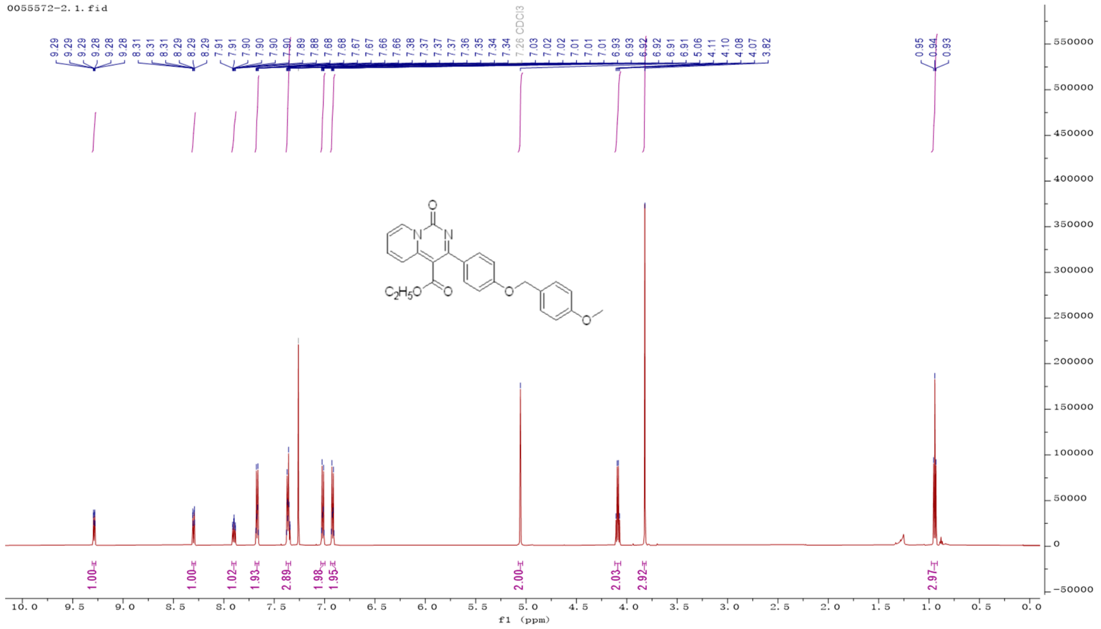

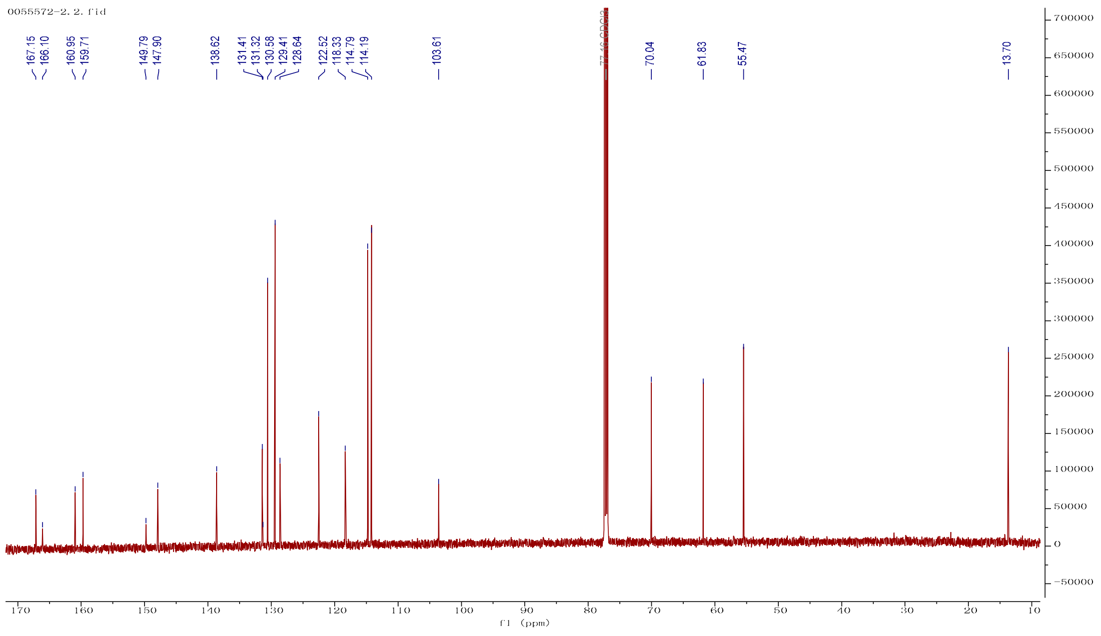

MS spectra

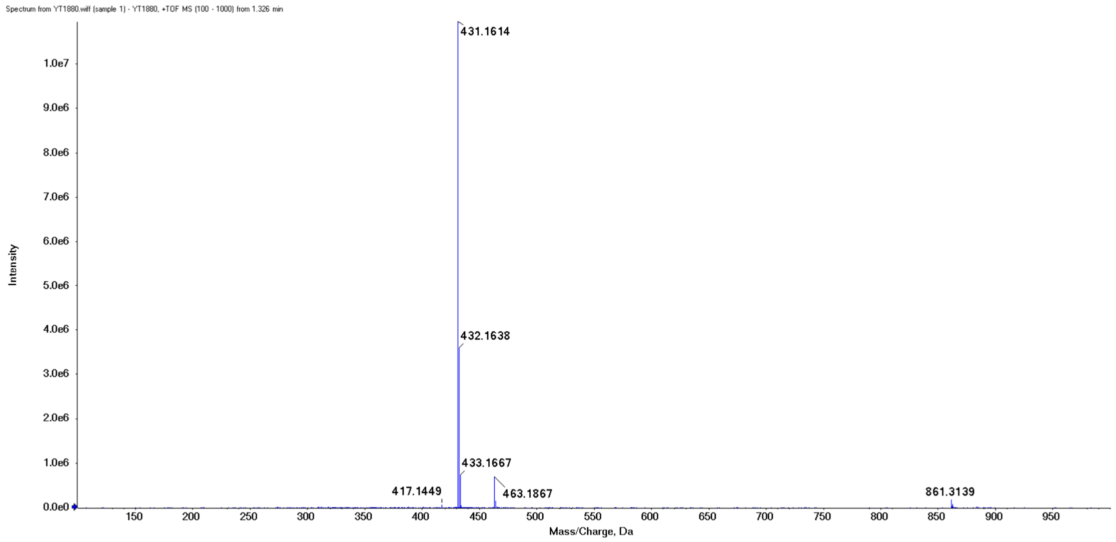

Formula Calculator Results

Measured m/z	Cal m/z	Error(mmu)	Error(ppm)	Ion Formula	Ion
431.16140	431.16015	1.3	2.9	$C_{25}H_{23}N_2O_5$	$[M+H]^+$

2.21 ^{1}H NMR, ^{13}C NMR and HRMS of M12

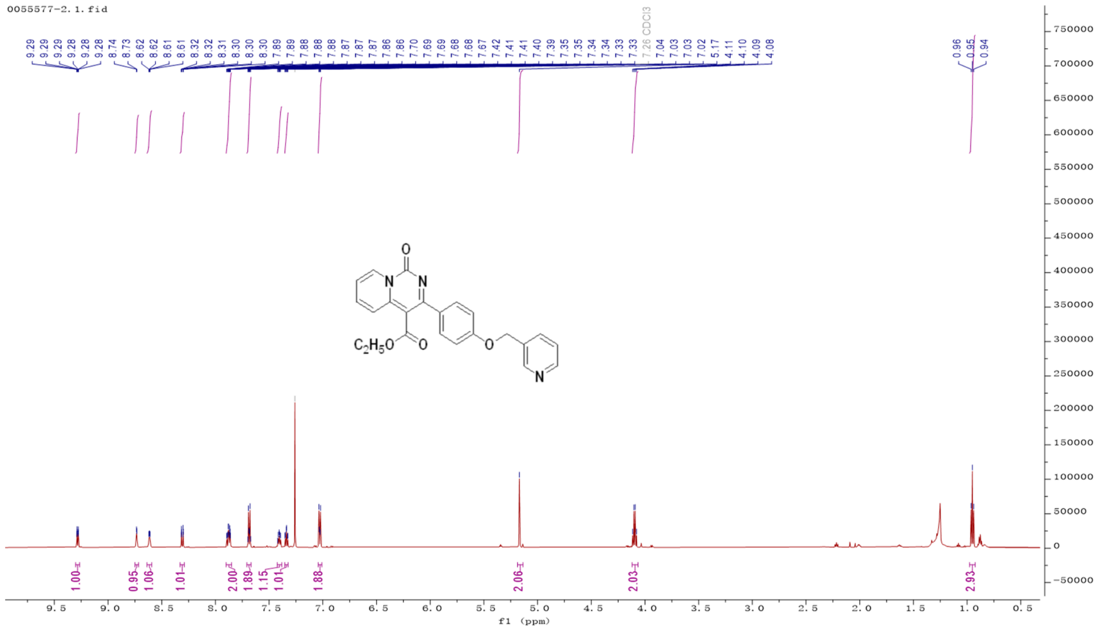

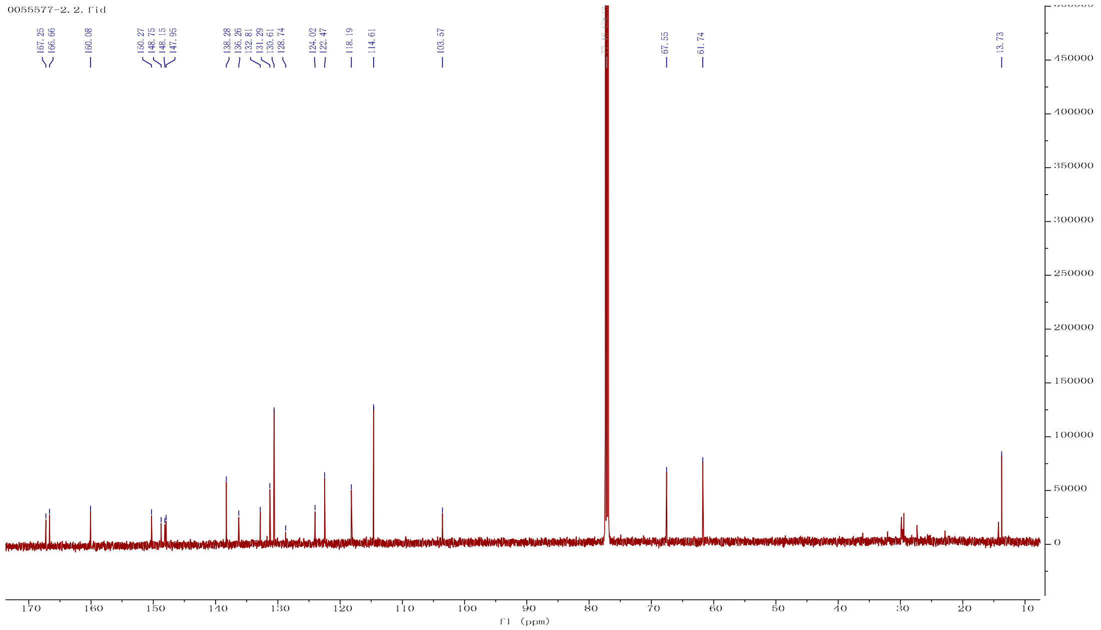

MS spectra

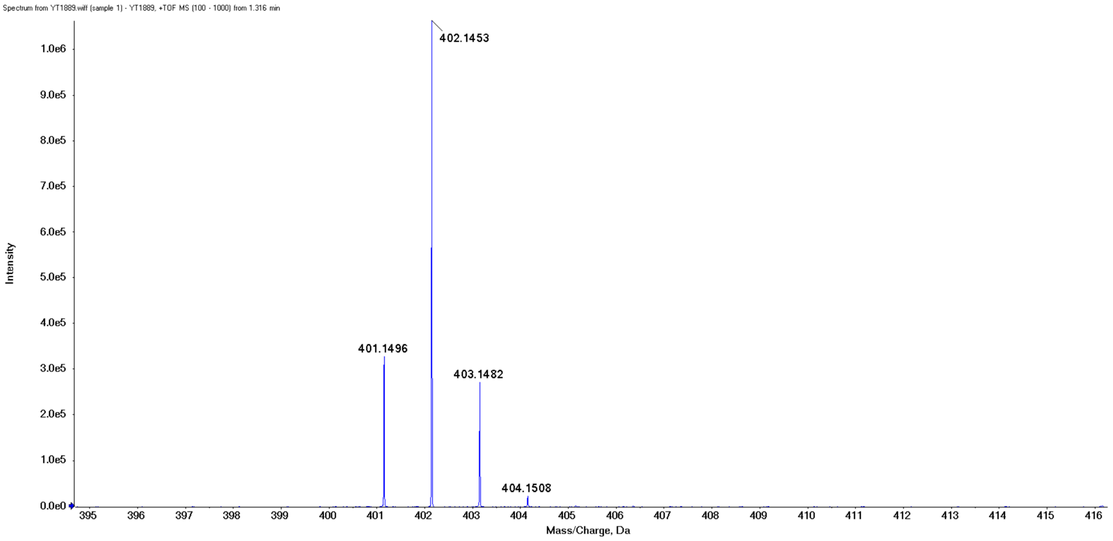

Formula Calculator Results

Measured m/z	Cal m/z	Error(mmu)	Error(ppm)	Ion Formula	Ion
402.14530	402.14483	0.5	1.2	$C_{23}H_{20}N_3O_4$	$[M+H]^+$

2.22 ^{1}H NMR, ^{13}C NMR and HRMS of M13

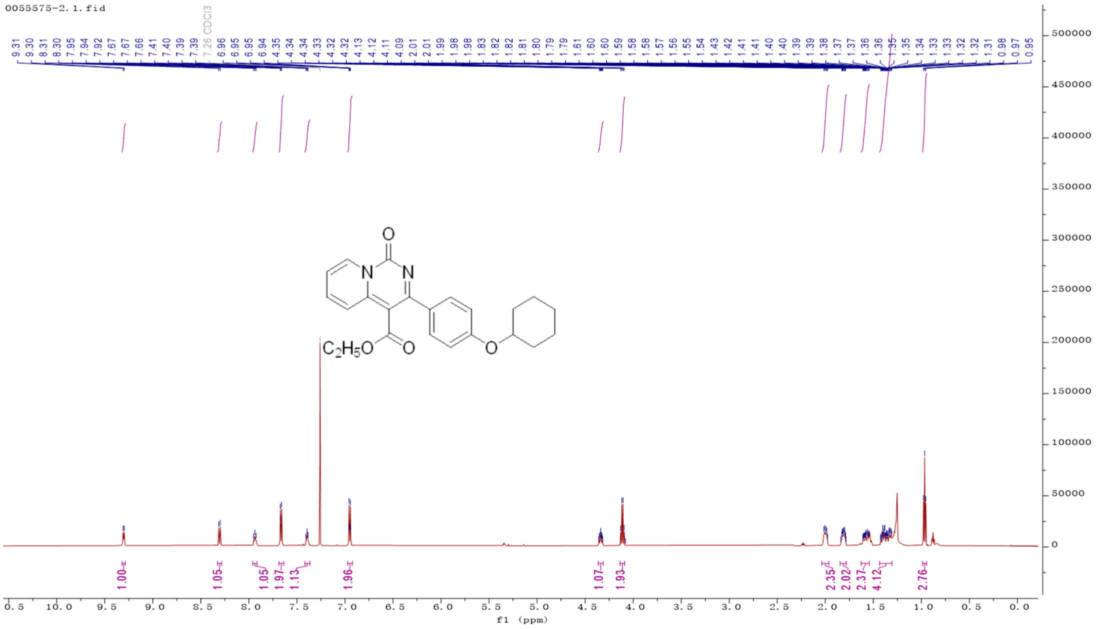

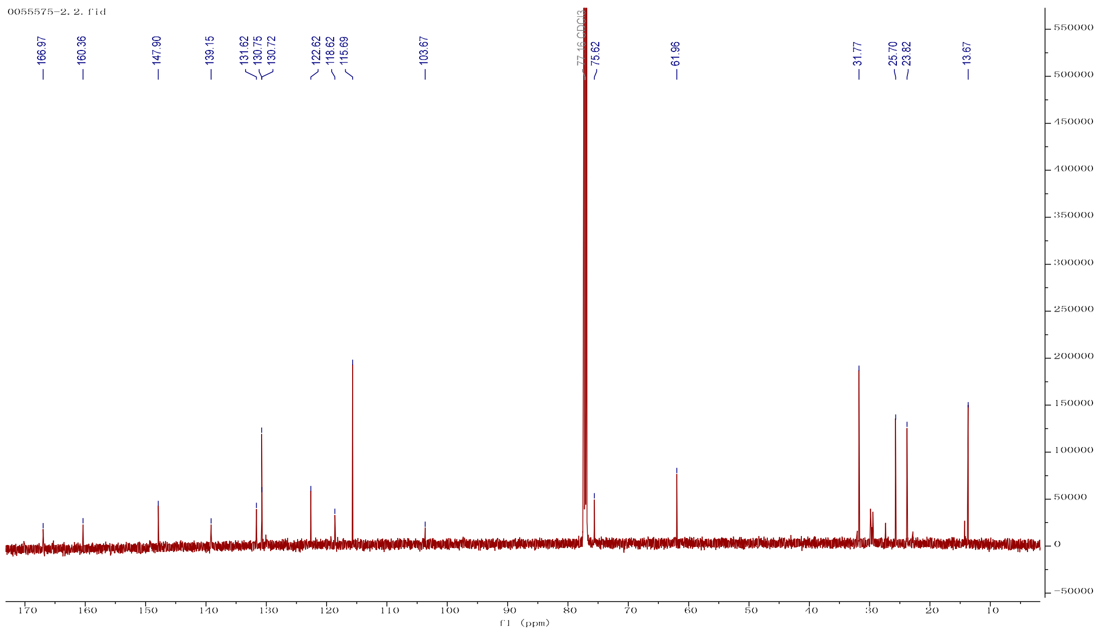

MS spectra

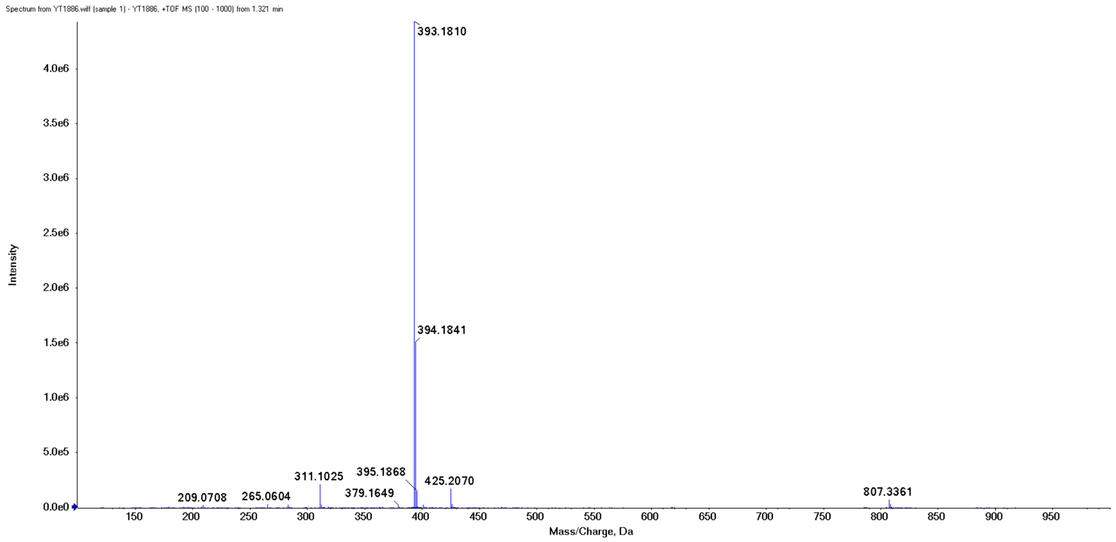

Formula Calculator Results

Measured m/z	Cal m/z	Error(mmu)	Error(ppm)	Ion Formula	Ion
393.18096	393.18088	0.1	0.2	$C_{23}H_{25}N_2O_4$	$[M+H]^+$

2.23 ^{1}H NMR, ^{13}C NMR and HRMS of M14

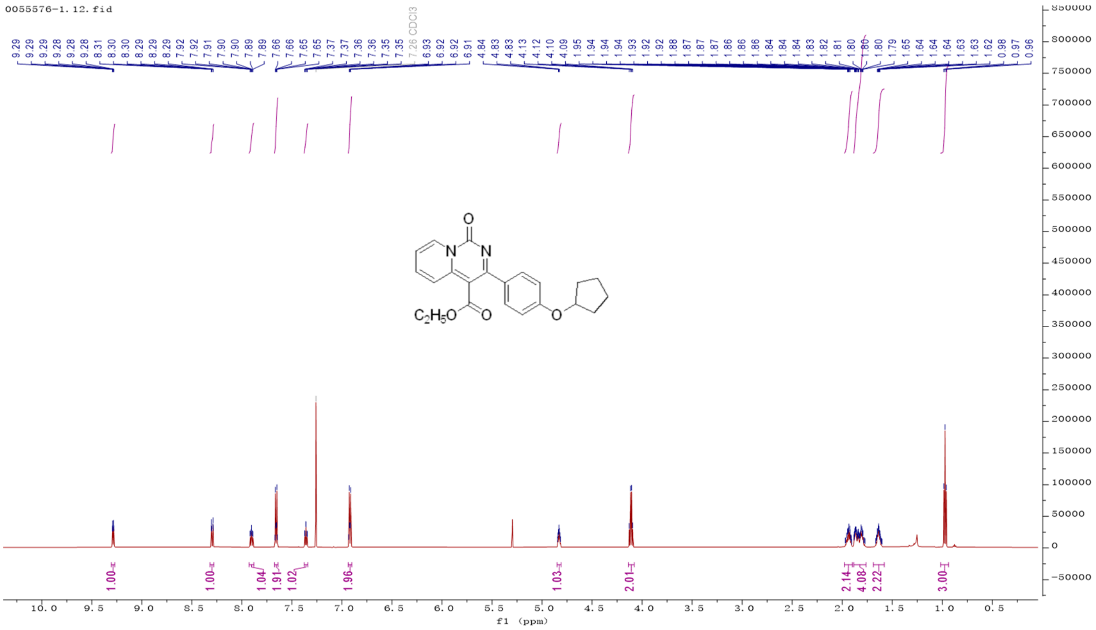

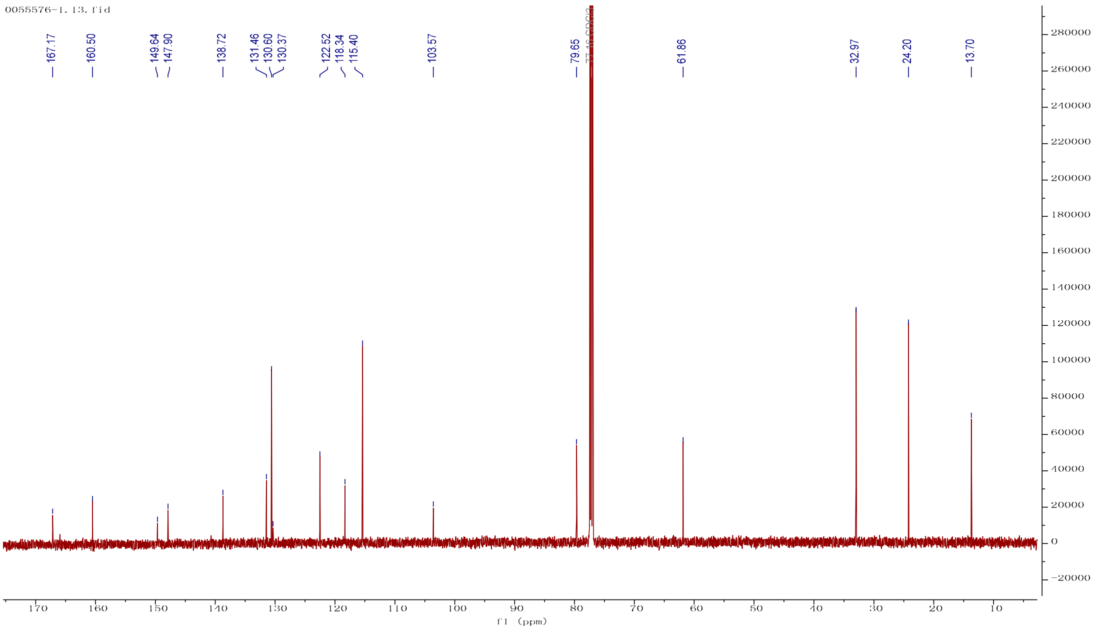

MS spectra

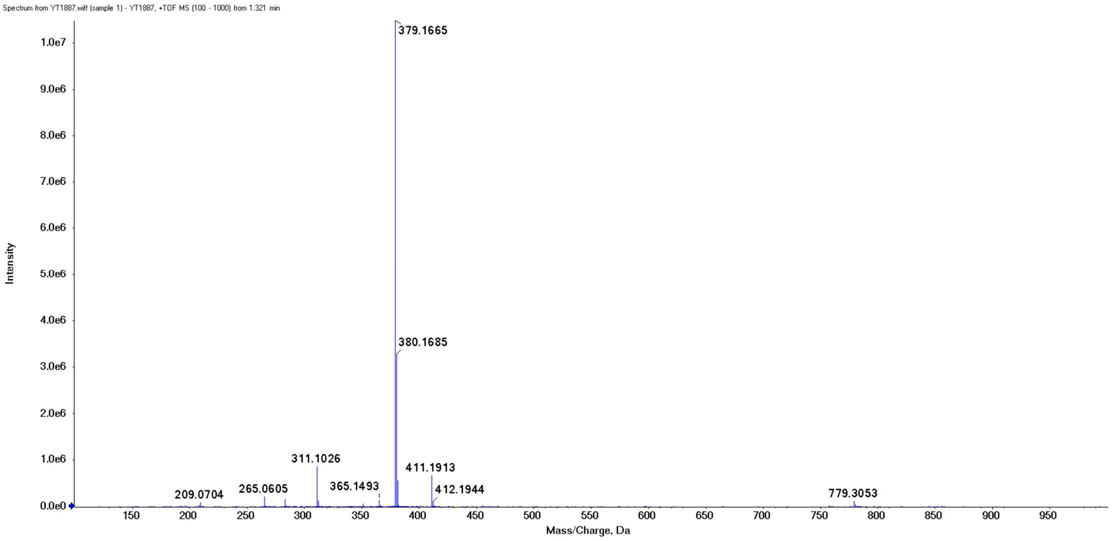

Formula Calculator Results

Measured m/z	Cal m/z	Error(mmu)	Error(ppm)	Ion Formula	Ion
379.16651	379.16523	1.3	3.4	$C_{22}H_{23}N_2O_4$	$[M+H]^+$

2.24 ^{1}H NMR, ^{13}C NMR and HRMS of N1

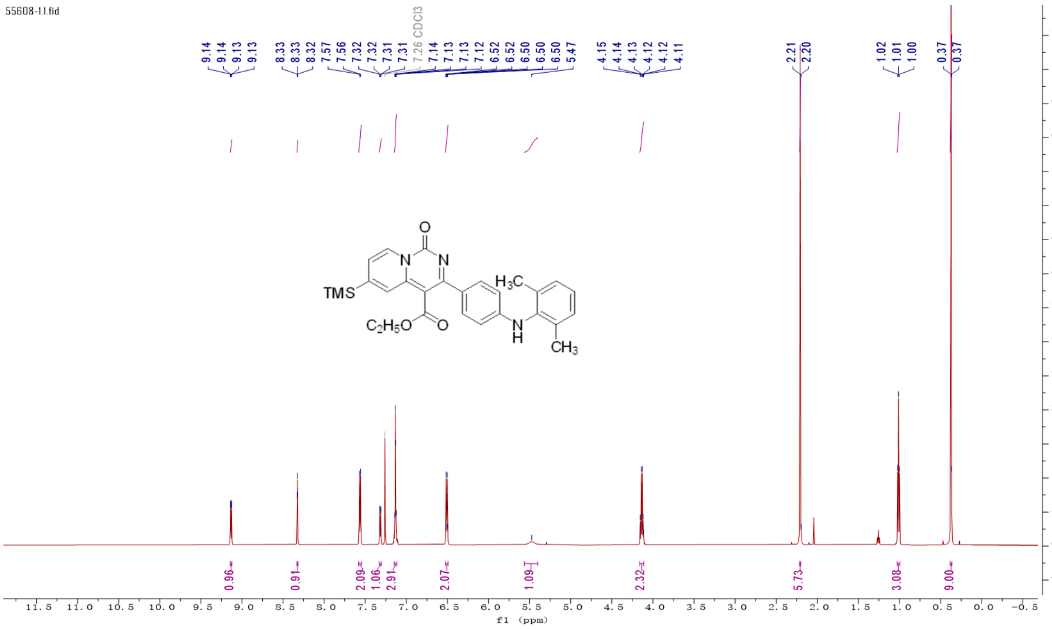

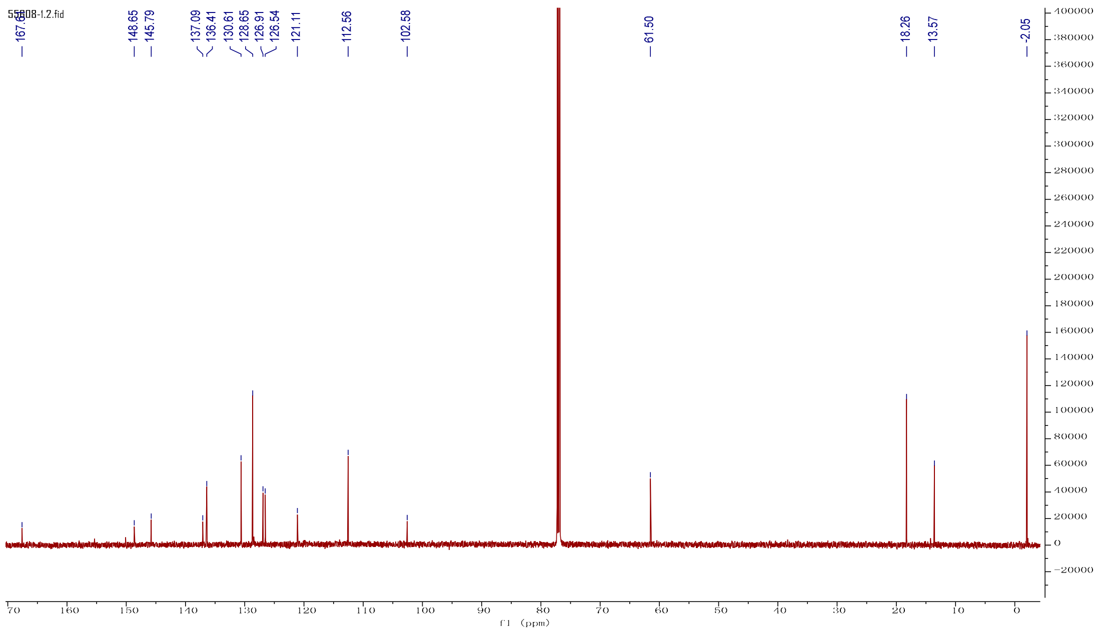

MS spectra

Spectrum from YT1897.wiff (sample 1) - YT1897, +TOF MS (100 - 1000) from 1.343 min

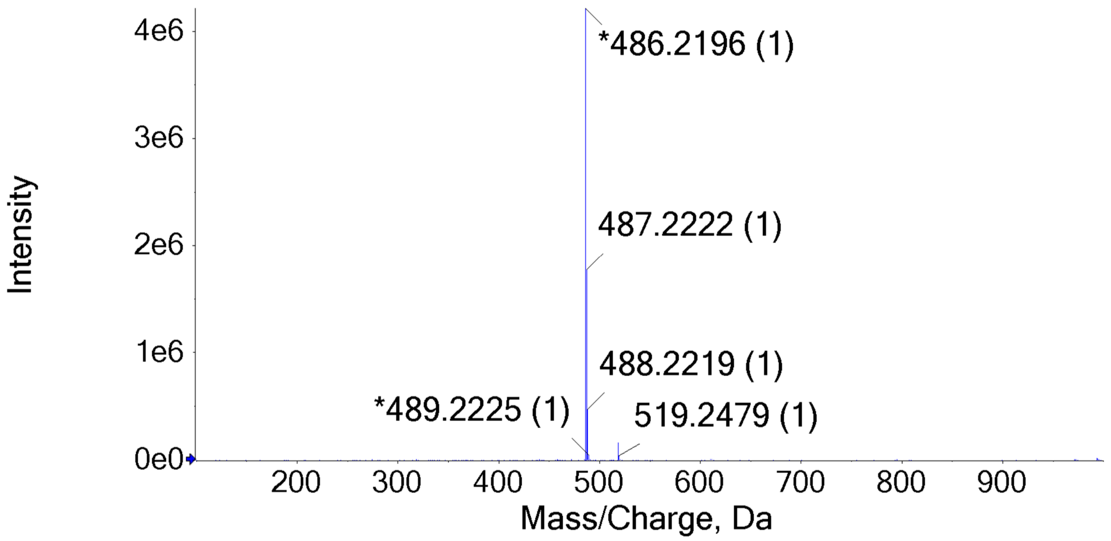

Formula Calculator Results

Measured m/z	Cal m/z	Error(mmu)	Error(ppm)	Ion Formula	Ion
486.2196	486.2207	-1.2	-2.5	$C_{28}H_{32}N_3O_3Si$	$[M+H]^+$

2.25 ^{1}H NMR, ^{13}C NMR and HRMS of N2

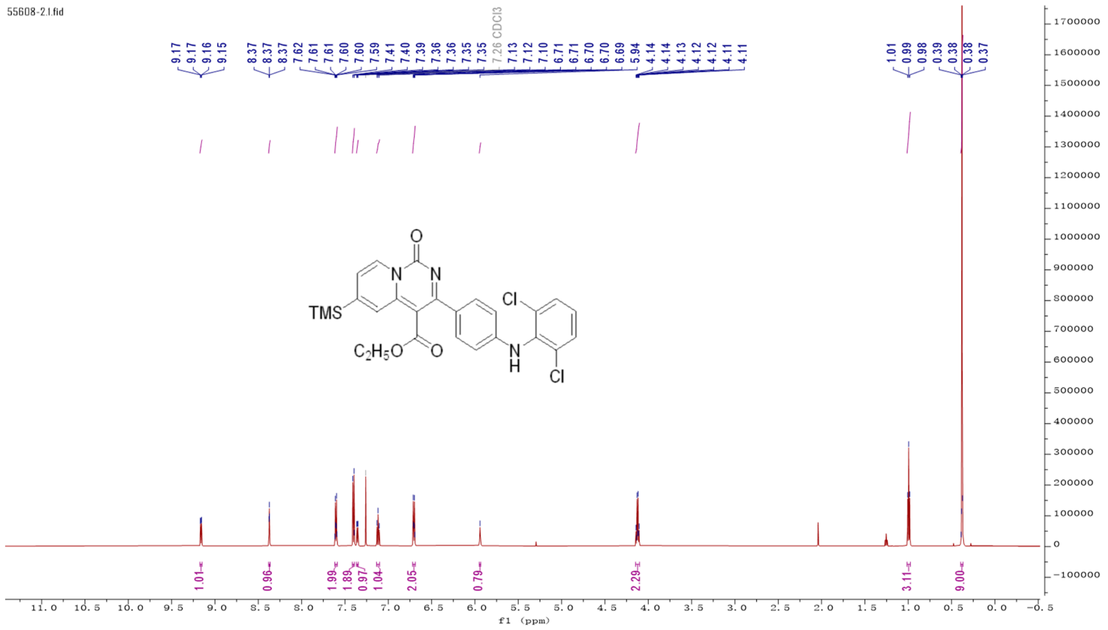
55608-2.1.fid
9.17 9.17 9.16 9.15 8.37 8.37 8.37 7.62 7.61 7.61 7.60 7.60 7.59 7.41 7.40 7.39 7.36 7.36 7.35 7.35 7.26 CDCl3 7.13 7.12 7.10 6.71 6.71 6.70 6.70 6.69 5.94 4.14 4.14 4.13 4.12 4.11 4.11
1.01 0.99 0.98 0.39 0.38 0.38 0.37
TMS
O
N
Cl
H
Cl
1.01 0.96 1.99 1.89 0.97 1.04 2.05 0.79 2.29 3.11 9.00
f1 (ppm)

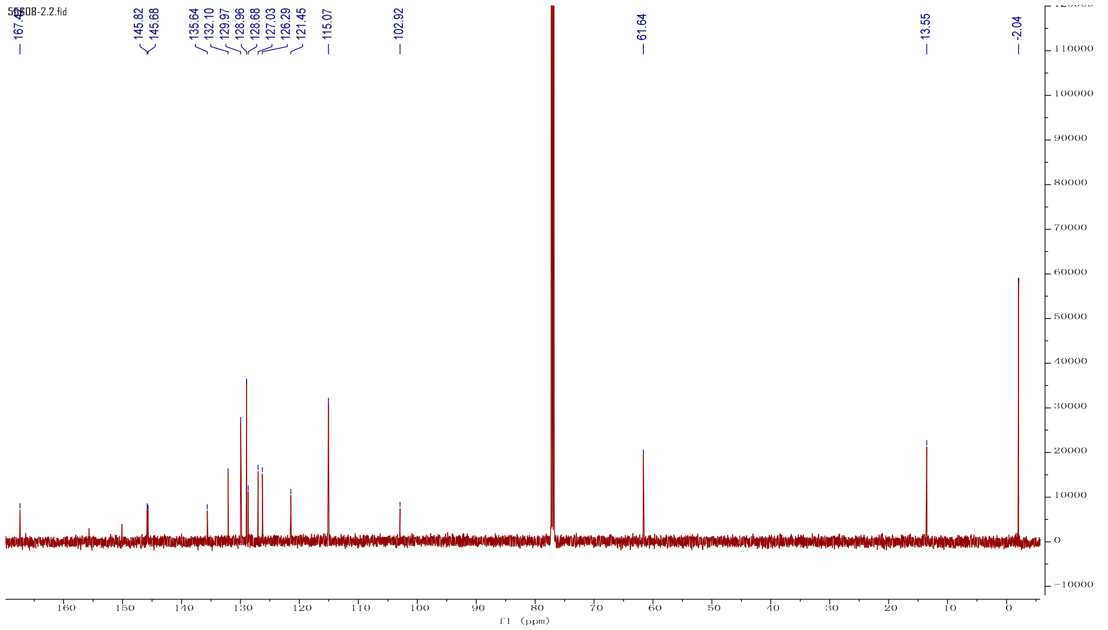
55608-2.2.fid
145.82 145.68 135.64 132.10 129.97 128.96 128.68 127.03 126.29 121.45 115.07 102.92 61.64 13.55 -2.04
f1 (ppm)

MS spectra

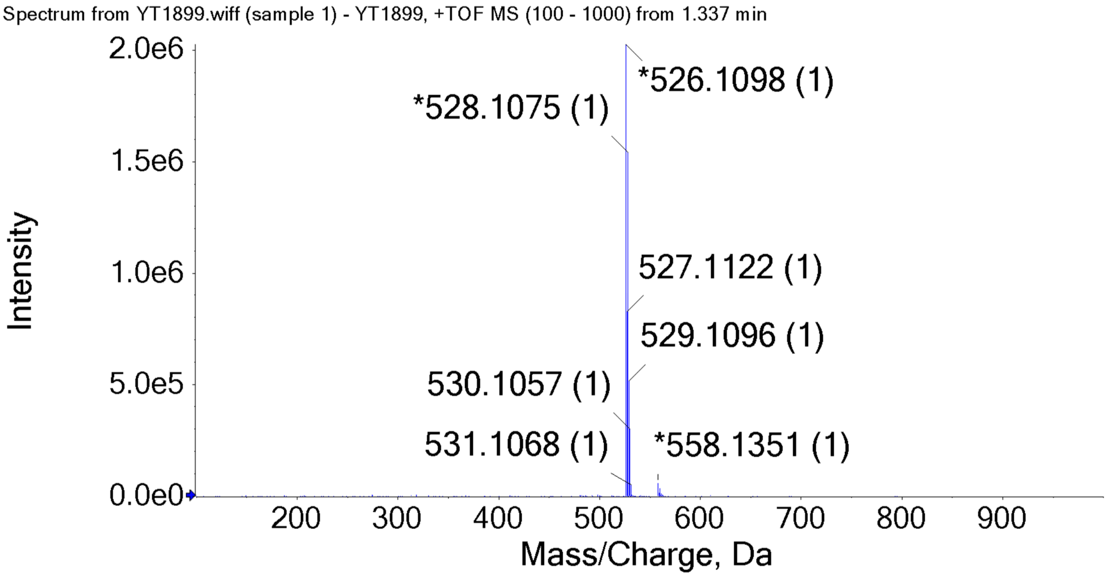

Formula Calculator Results

Measured m/z	Cal m/z	Error(mmu)	Error(ppm)	Ion Formula	Ion
526.1098	526.1115	-1.7	-3.2	$C_{26}H_{26}Cl_2N_3O_3Si$	$[M+H]^+$

2.26 ^{1}H NMR, ^{13}C NMR and HRMS of N3

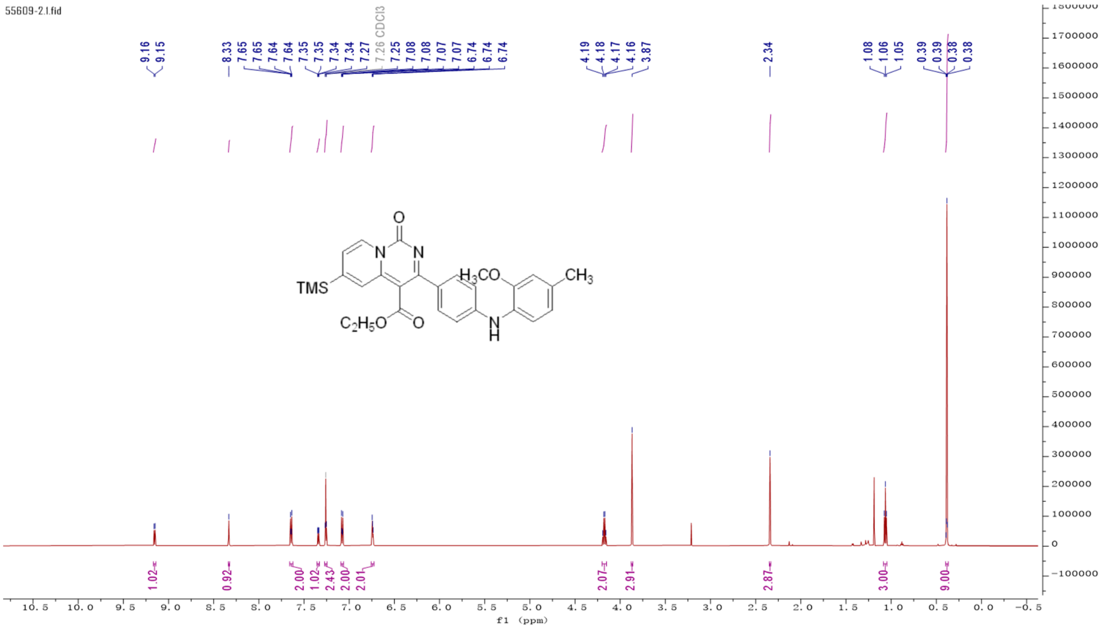

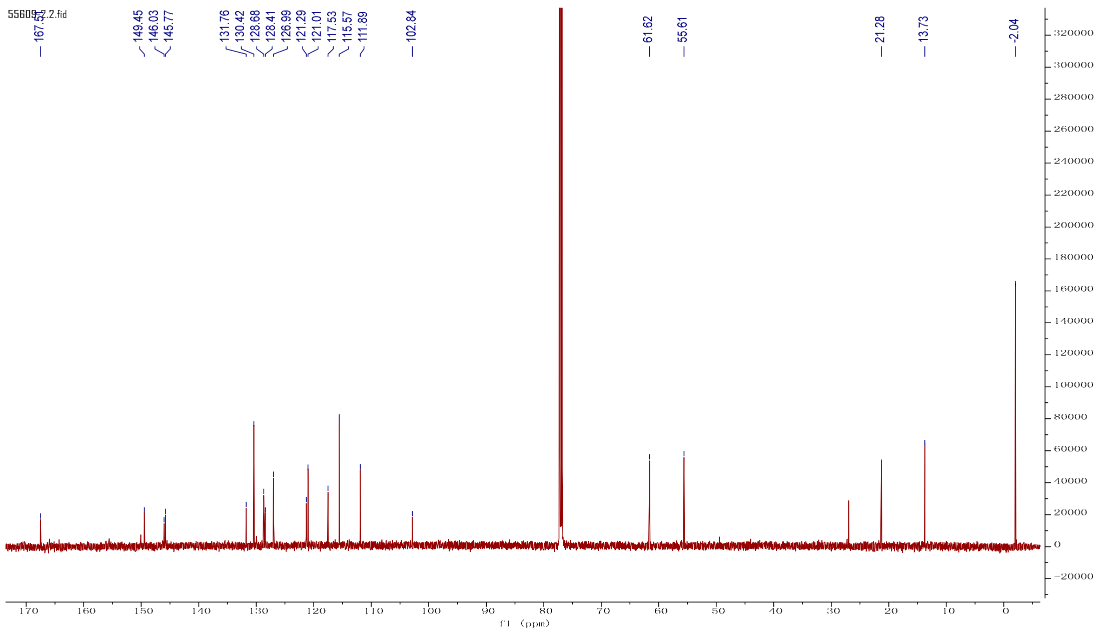

MS spectra

Spectrum from YT1900.wiff (sample 1) - YT1900, +TOF MS (100 - 1000) from 1.332 min

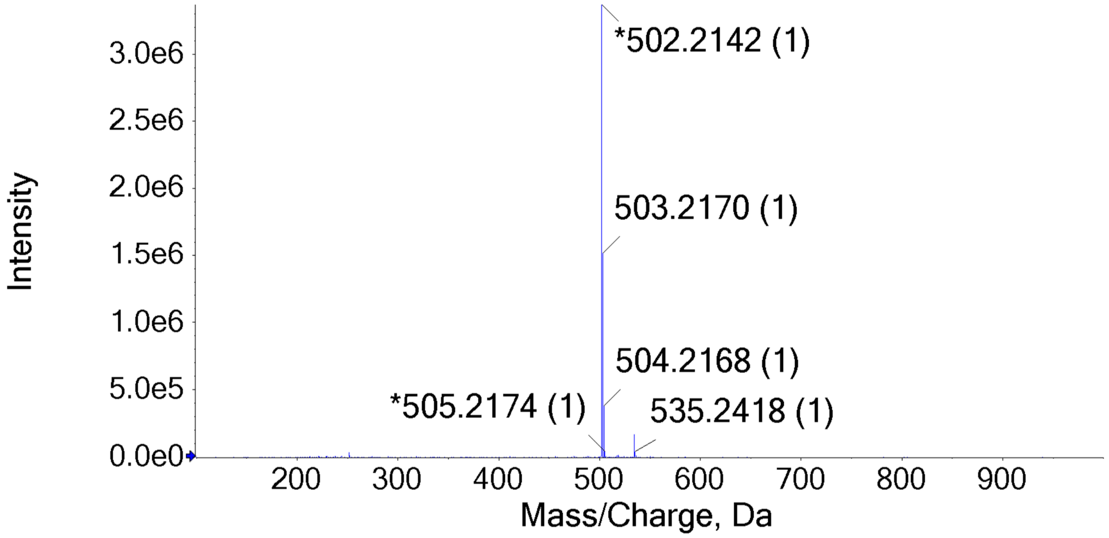

Formula Calculator Results

Measured m/z	Cal m/z	Error(mmu)	Error(ppm)	Ion Formula	Ion
502.2142	502.2156	-1.4	-2.8	$C_{28}H_{32}N_3O_4Si$	$[M+H]^+$

2.27 ^{1}H NMR, ^{13}C NMR and HRMS of N4

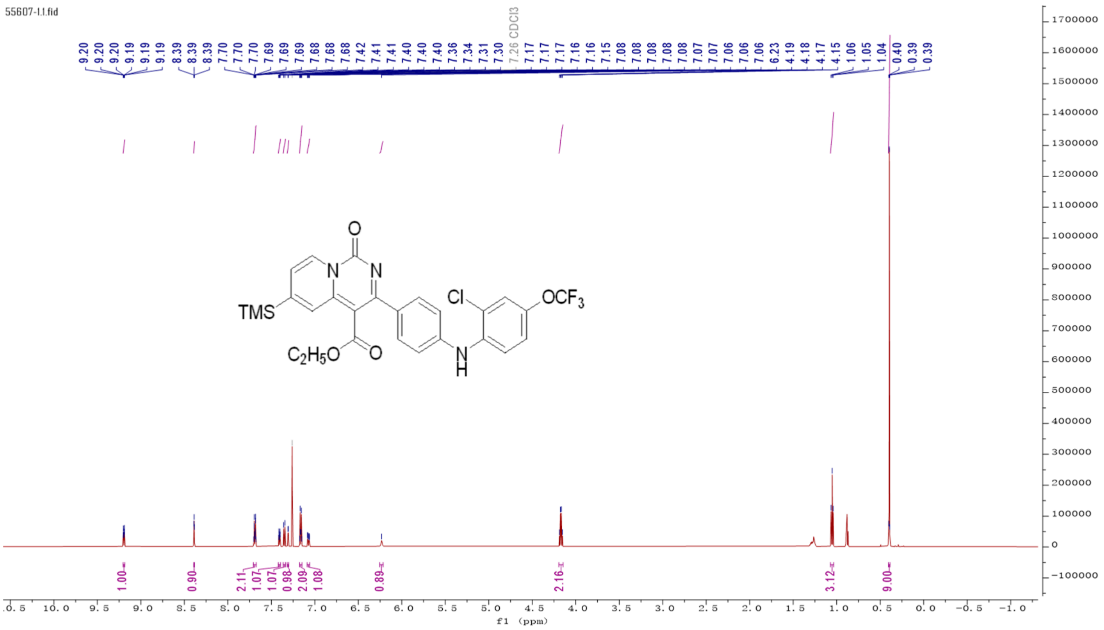

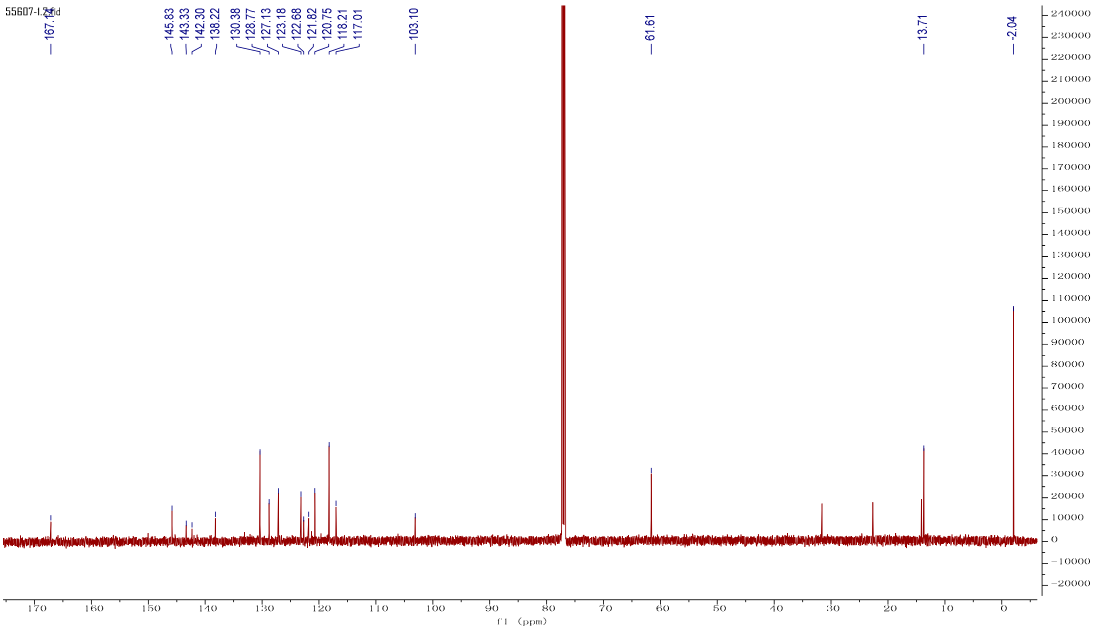

MS spectra

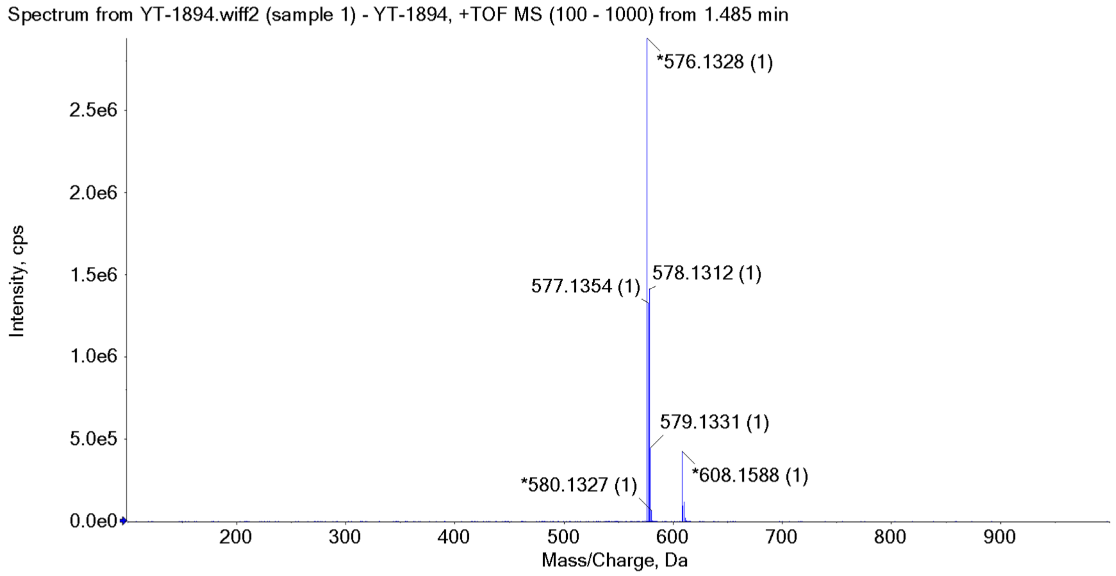

Formula Calculator Results

Measured m/z	Cal m/z	Error(mmu)	Error(ppm)	Ion Formula	Ion
576.1328	576.1328	0	0	$C_{27}H_{26}ClF_3N_3O_4Si$	$[M+H]^+$

2.28 ^{1}H NMR, ^{13}C NMR and HRMS of O1

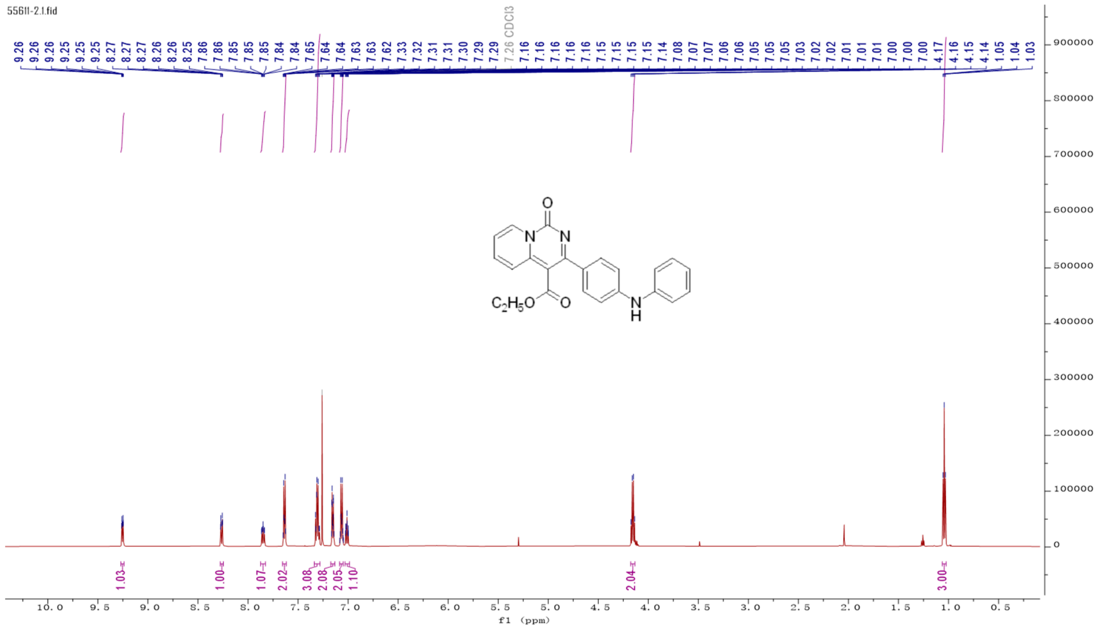

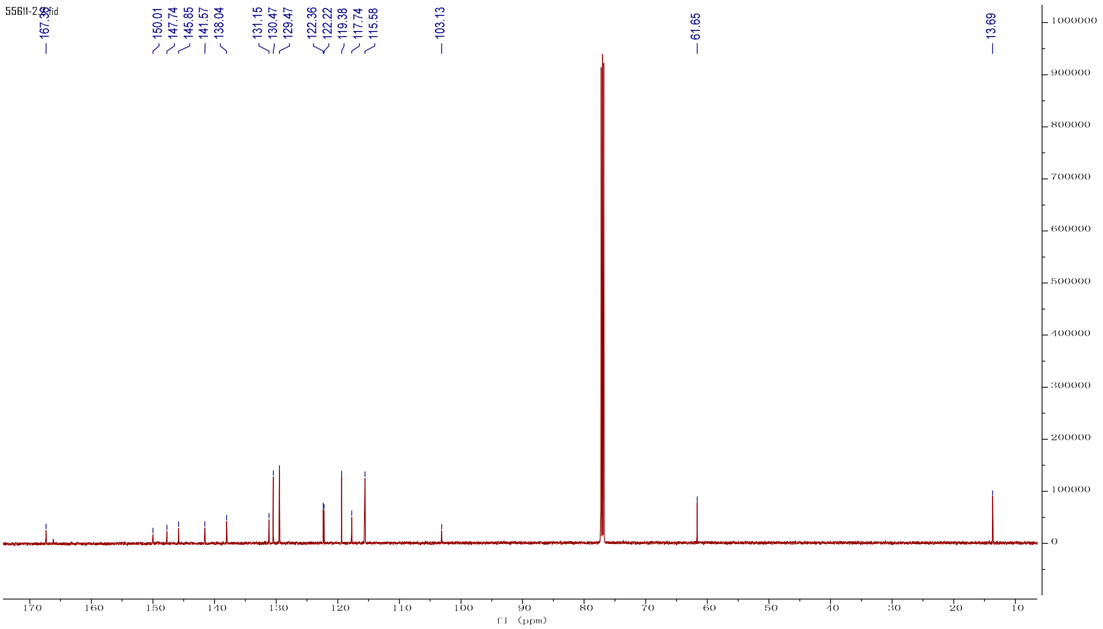

MS spectra

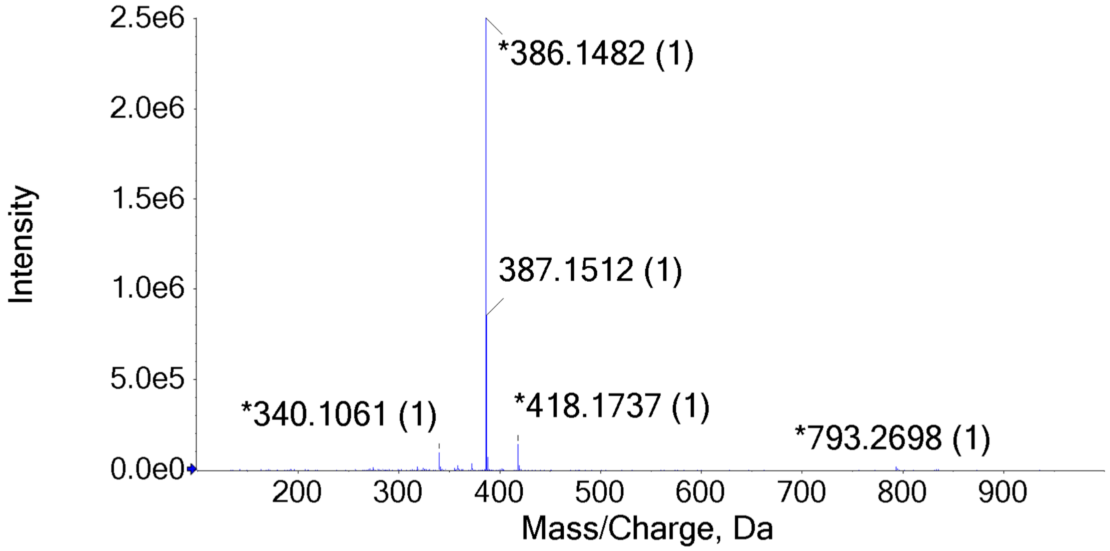

Formula Calculator Results

Measured m/z	Cal m/z	Error(mmu)	Error(ppm)	Ion Formula	Ion
386.1482	386.1499	-1.7	-4.4	$C_{23}H_{20}N_3O_3$	$[M+H]^+$

2.29 ^{1}H NMR, ^{13}C NMR and HRMS of O2

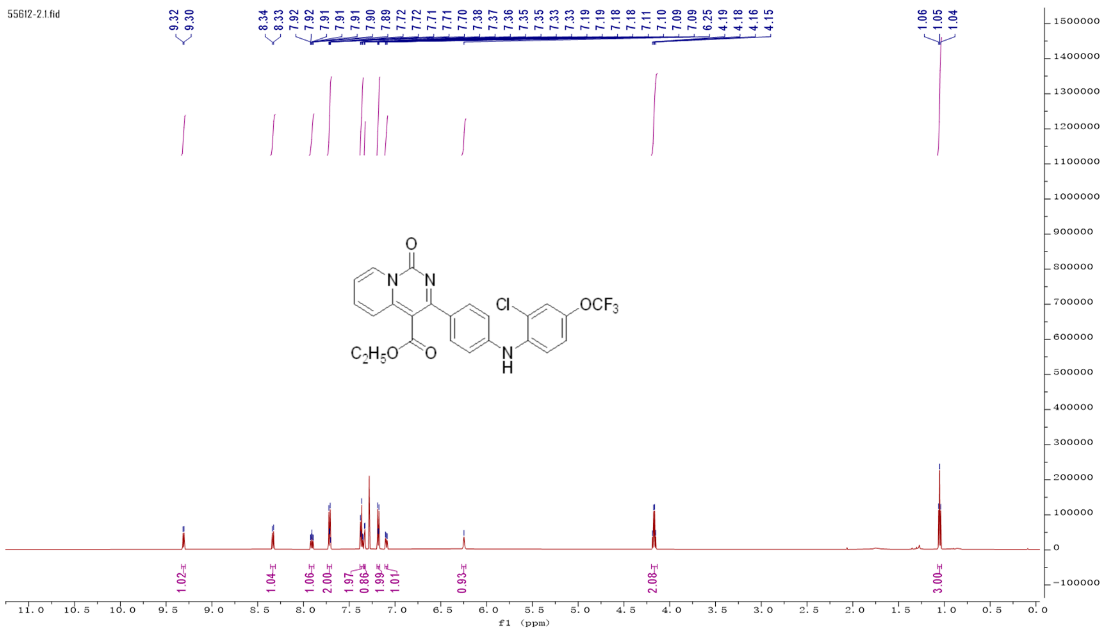
55612-2.1.fid
OCF3
Cl
N
H
C2H5O
O
f1 (ppm)

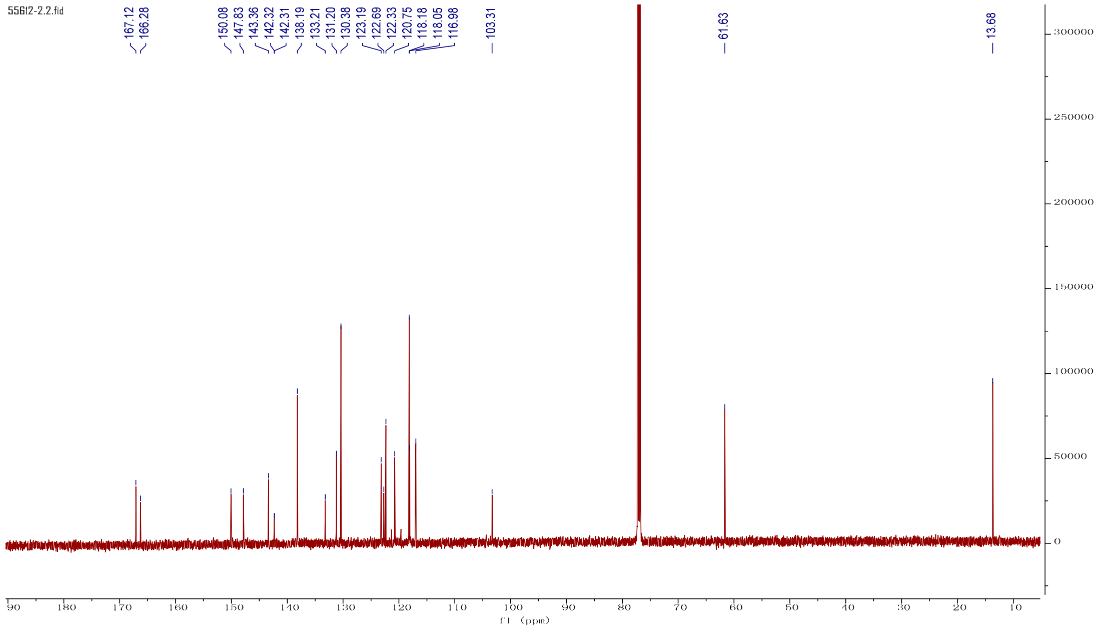
55612-2.2.fid
167.12
166.28
150.08
147.83
143.36
142.32
142.31
138.19
133.21
131.20
130.38
123.19
122.69
122.33
120.75
118.18
118.05
116.98
103.31
61.63
13.68
f1 (ppm)

MS spectra

Spectrum from YT1820.wiff (sample 1) - YT1820, +TOF MS (100 - 1000) from 1.326 min

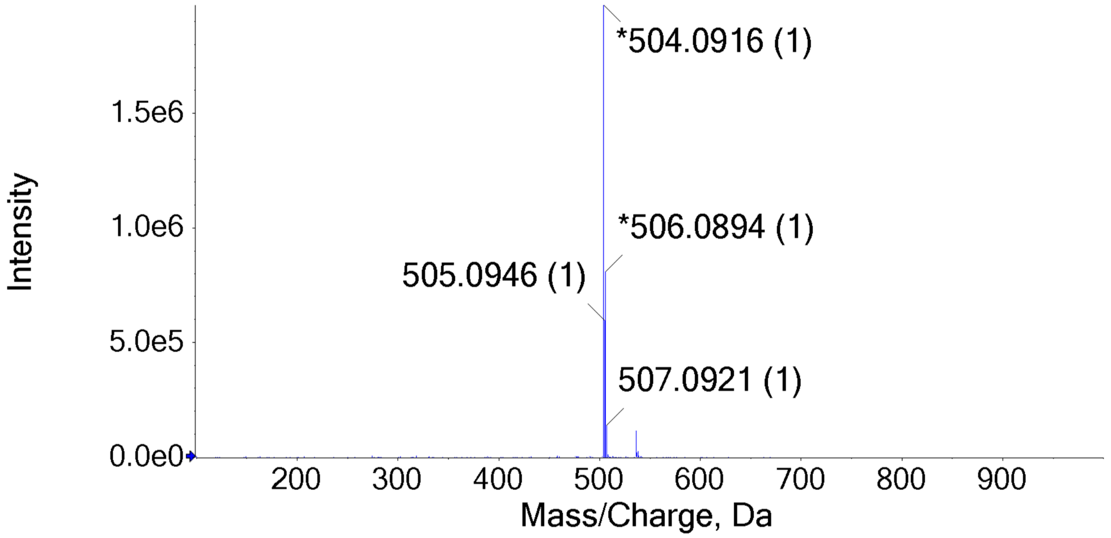

Formula Calculator Results

Measured m/z	Cal m/z	Error(mmu)	Error(ppm)	Ion Formula	Ion
504.0916	504.0932	-1.6	-3.2	$C_{24}H_{18}ClF_3N_3O_4$	$[M+H]^+$

2.30 ^{1}H NMR, ^{13}C NMR and HRMS of O3

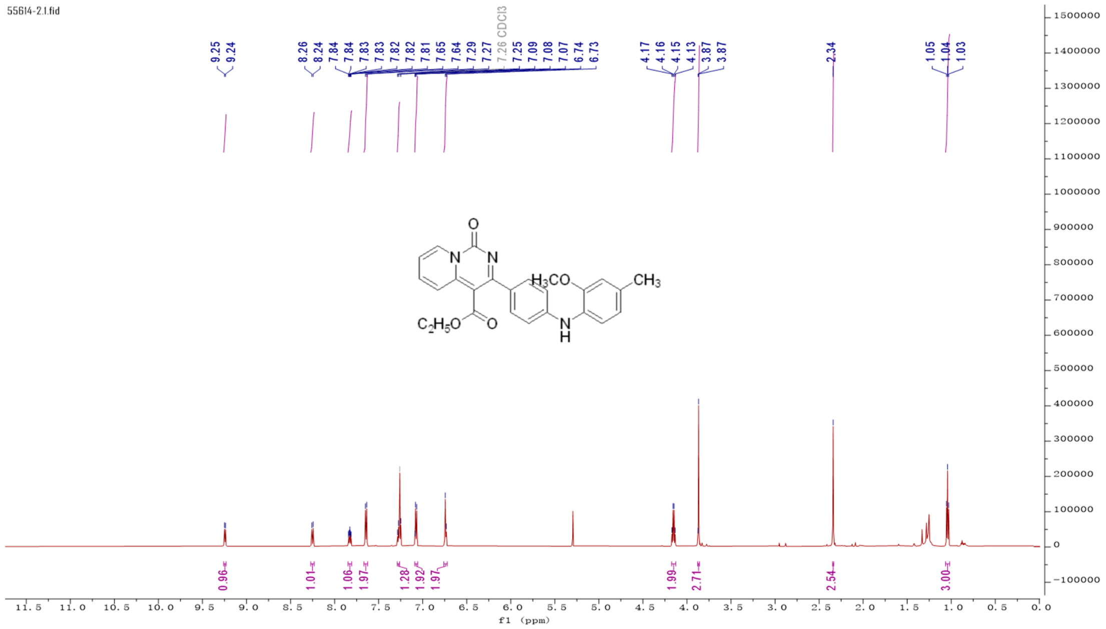

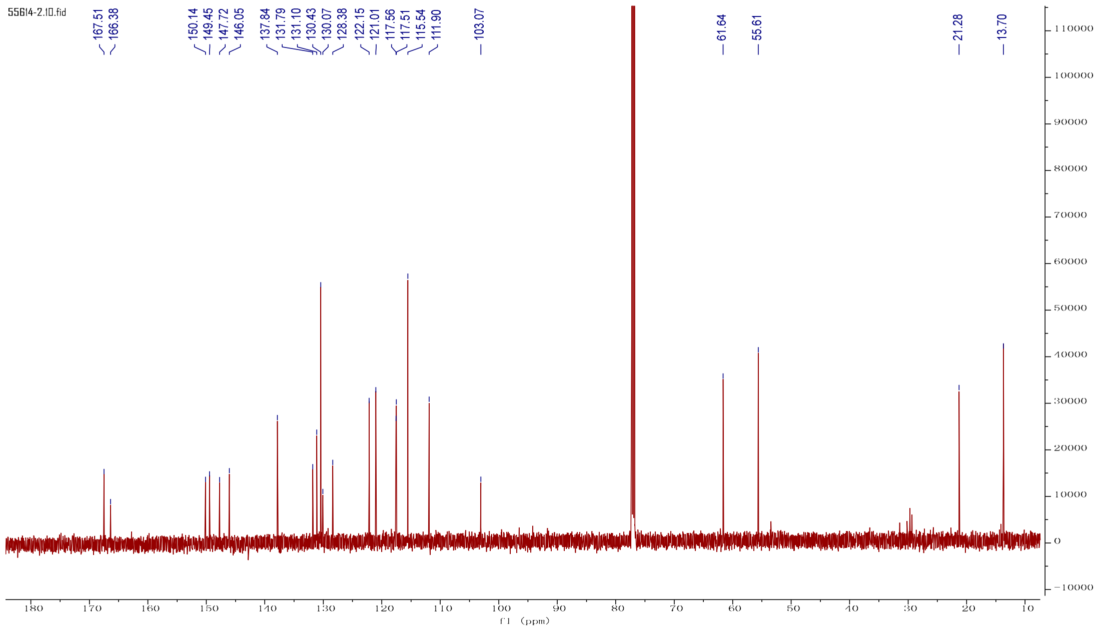

MS spectra

Spectrum from YT1822.wiff (sample 1) - YT1822, +TOF MS (100 - 1000) from 1.321 min

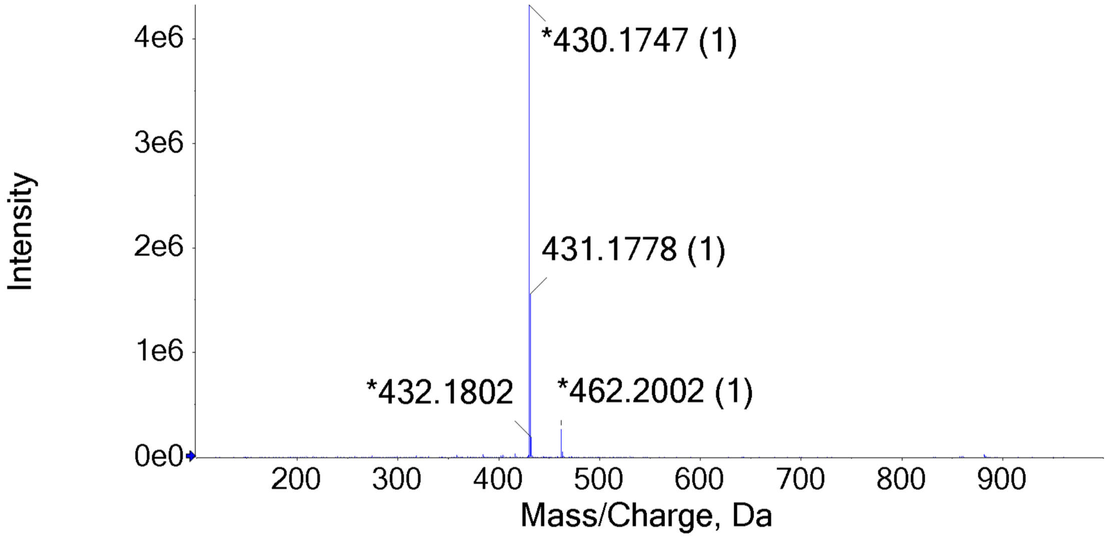

Formula Calculator Results

Measured m/z	Cal m/z	Error(mmu)	Error(ppm)	Ion Formula	Ion
430.1747	430.1761	-1.5	-3.4	$C_{25}H_{24}N_3O_4$	$[M+H]^+$

2.31 ^{1}H NMR and HRMS of O4

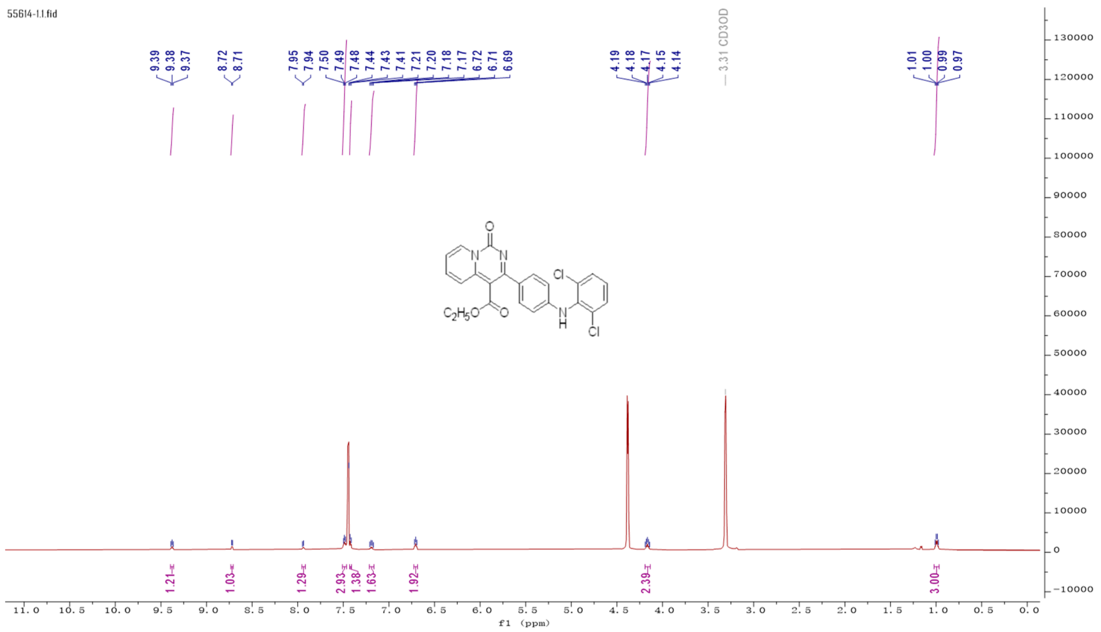

MS spectra

Spectrum from YT1898.wiff (sample 1) - YT1898, +TOF MS (100 - 1000) from 1.310 min

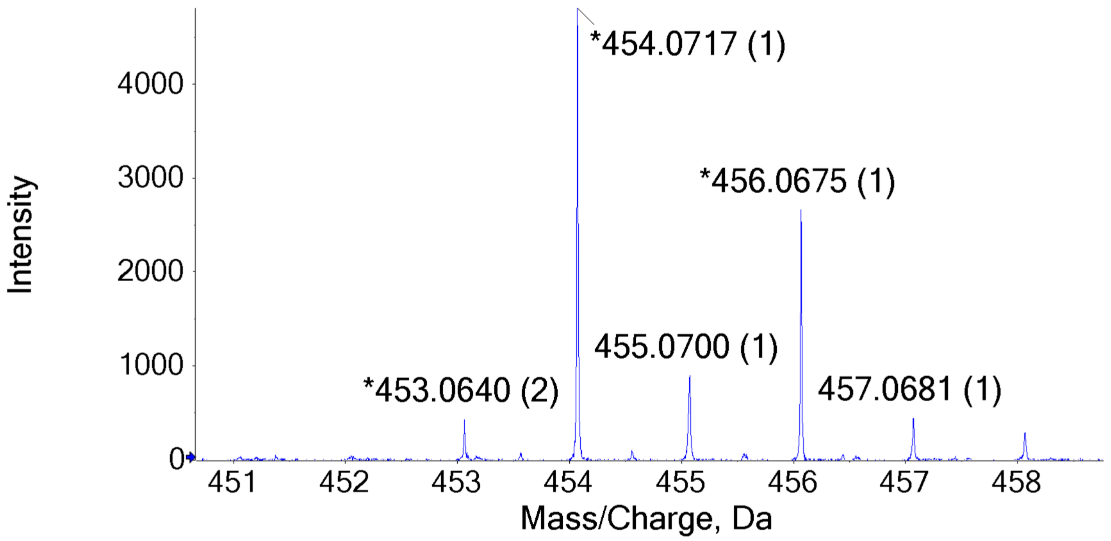

Formula Calculator Results

Measured m/z	Cal m/z	Error(mmu)	Error(ppm)	Ion Formula	Ion
454.0717	454.0719	-0.3	-0.6	$C_{23}H_{18}Cl_2N_3O_3$	$[M+H]^+$

2.32 ^{1}H NMR, ^{13}C NMR and HRMS of O5

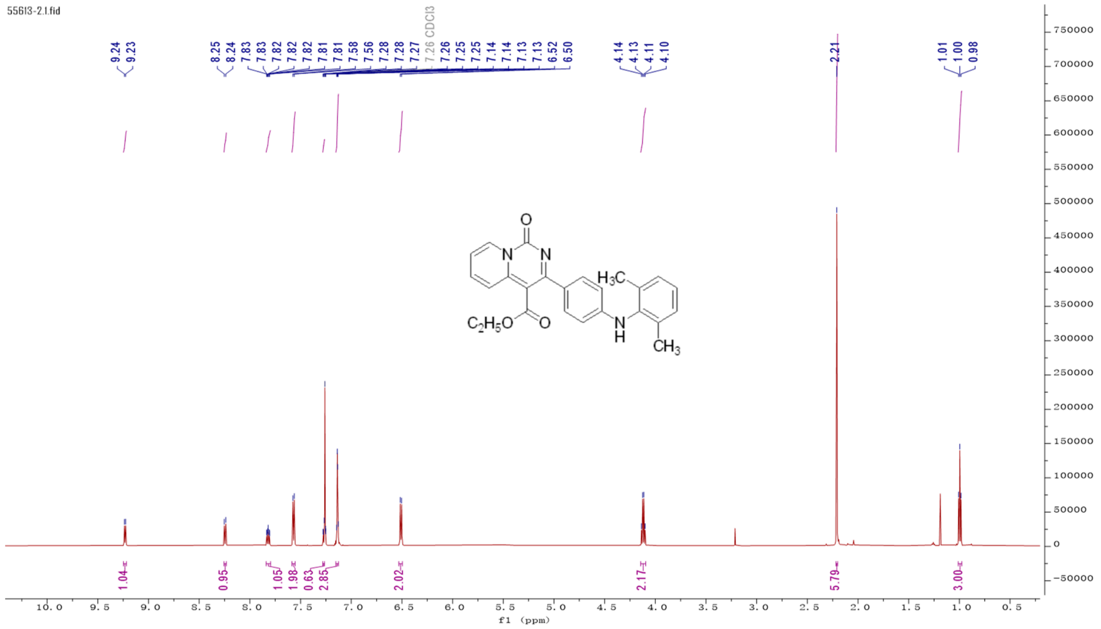
55613-2.1.fid
9.24
9.23
8.25
8.24
7.83
7.83
7.82
7.82
7.82
7.81
7.81
7.58
7.56
7.28
7.28
7.27
7.26 CDCl3
7.26
7.25
7.25
7.14
7.14
7.13
7.13
6.52
6.50
4.14
4.13
4.11
4.10
2.21
1.01
1.00
0.98
1.04
0.95
1.05
1.98
0.63
2.85
2.02
2.17
5.79
3.00
f1 (ppm)

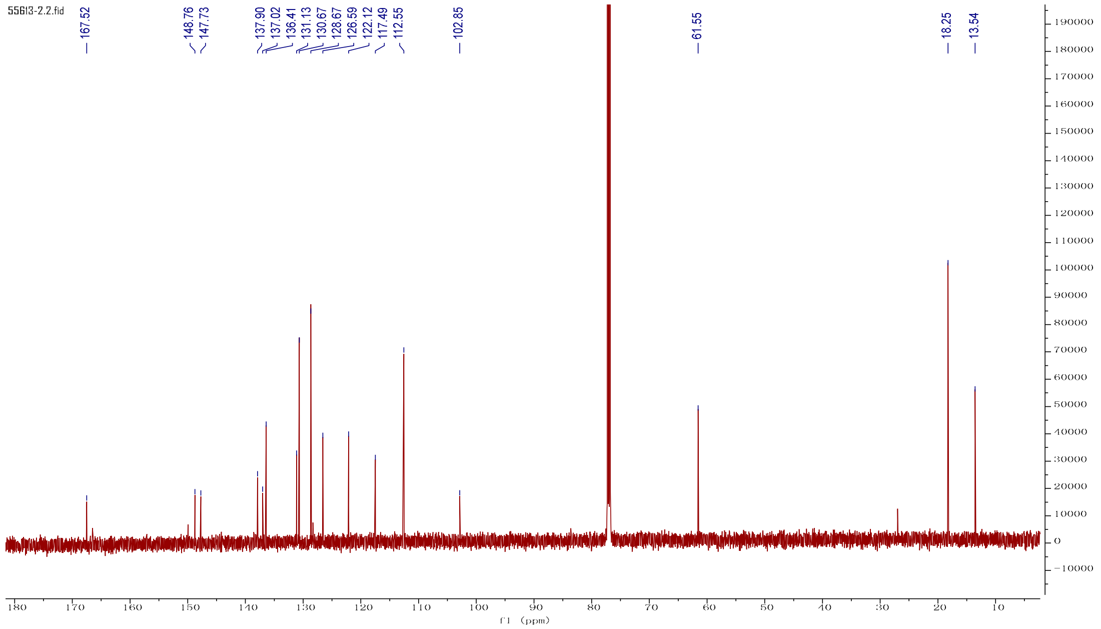
55613-2.2.fid
167.52
148.76
147.73
137.90
137.02
136.41
131.13
130.67
128.67
126.59
122.12
117.49
112.55
102.85
61.55
18.25
13.54
f1 (ppm)

MS spectra

Spectrum from YT1896.wiff (sample 1) - YT1896, +TOF MS (100 - 1000) from 1.321 min

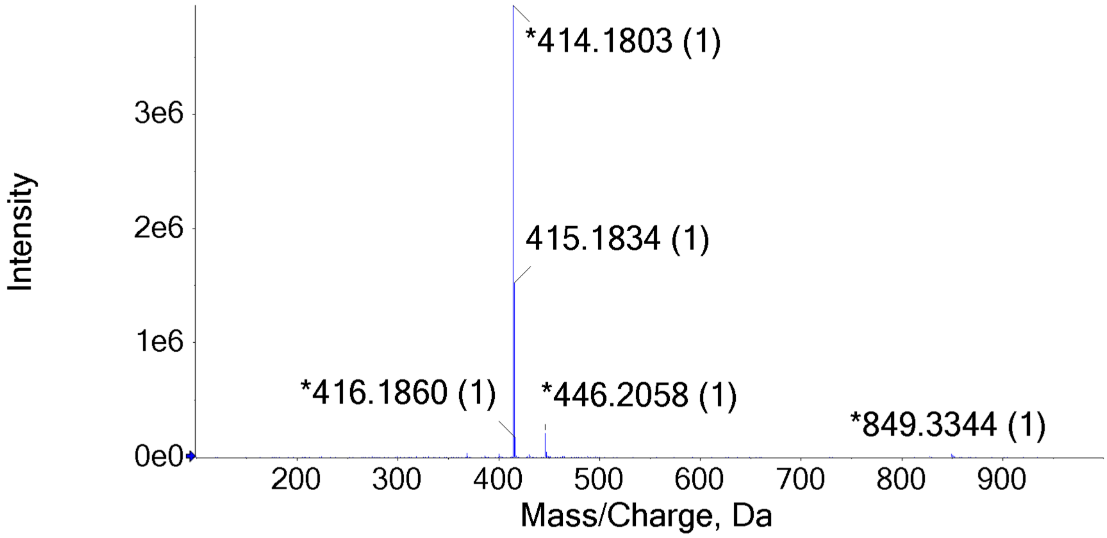

Formula Calculator Results

Measured m/z	Cal m/z	Error(mmu)	Error(ppm)	Ion Formula	Ion
414.1803	414.1812	-0.9	-2.3	$C_{25}H_{24}N_3O_3$	$[M+H]^+$

2.33 ^{1}H NMR, ^{13}C NMR and HRMS of O6

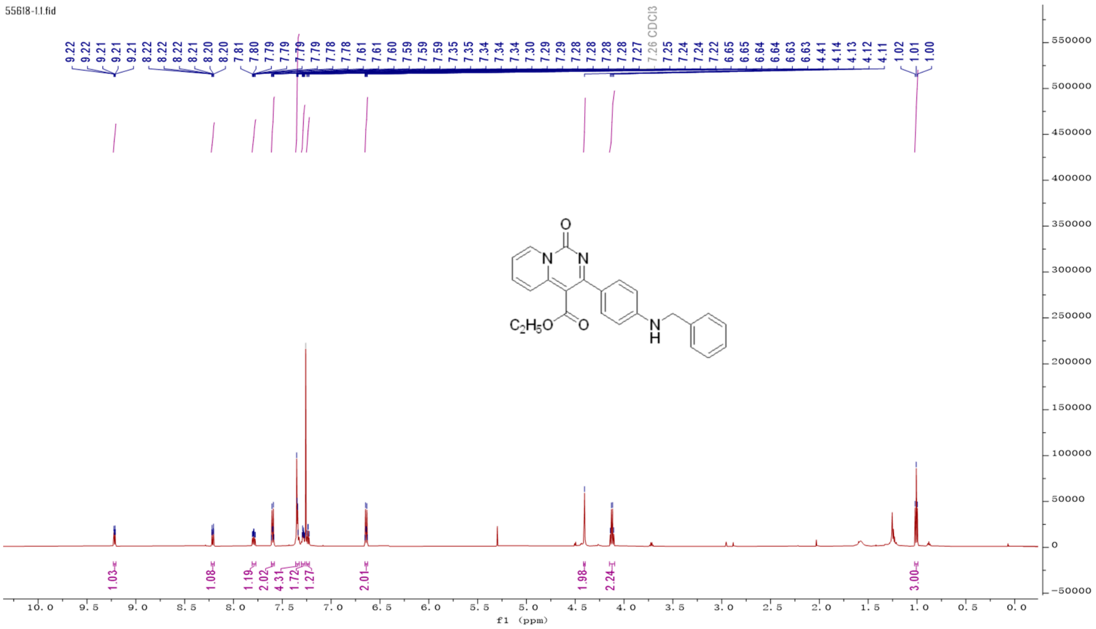

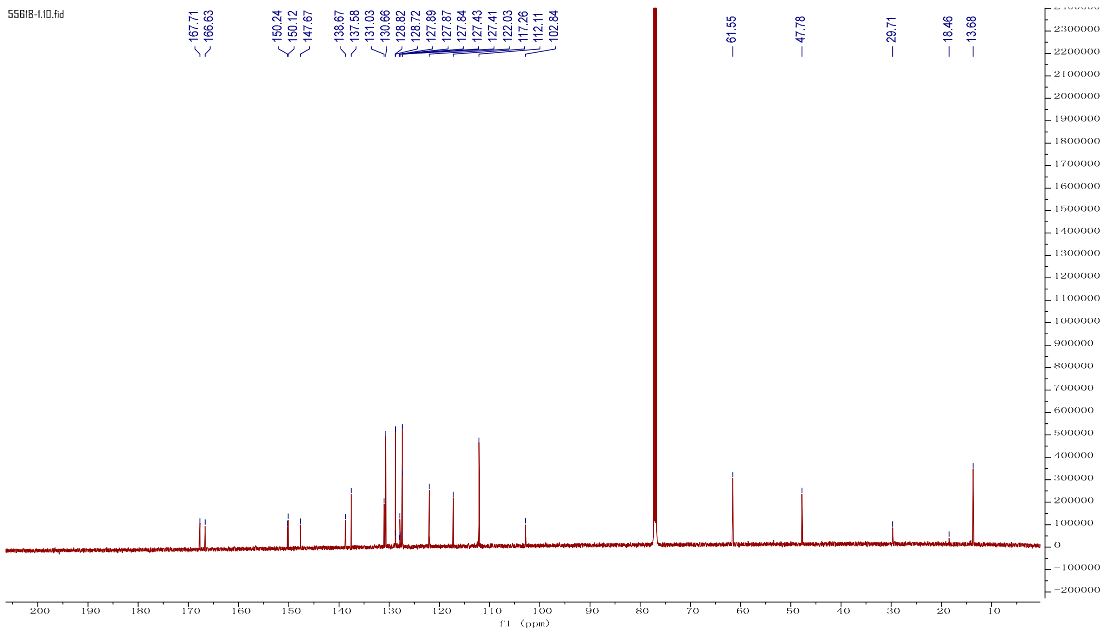

MS spectra

Spectrum from YT1904.wiff2 (sample 1) - YT1904, +TOF MS (100 - 1000) from 1.310 min

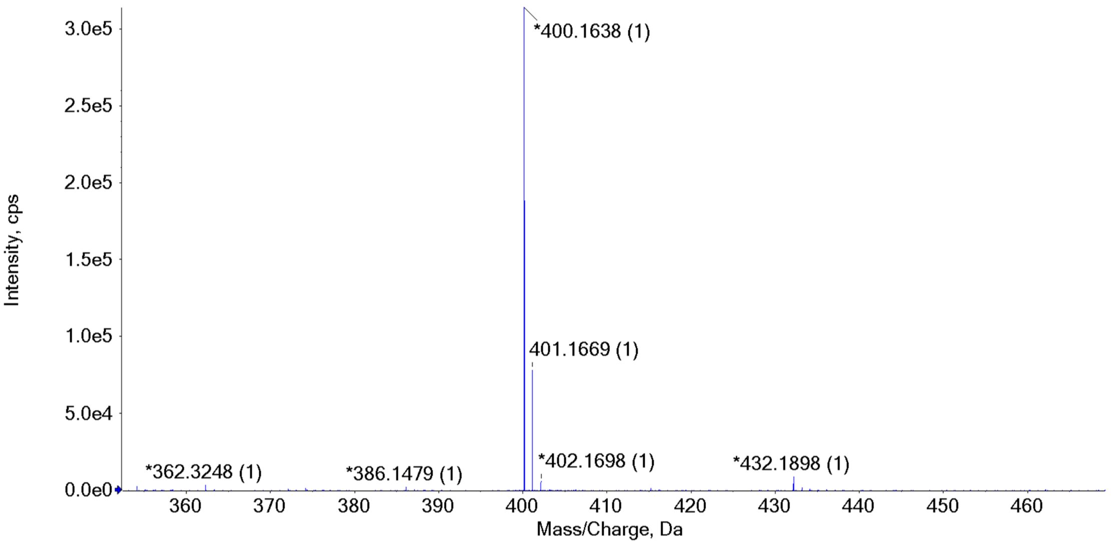

Formula Calculator Results

Measured m/z	Cal m/z	Error(mmu)	Error(ppm)	Ion Formula	Ion
400.1638	400.1656	-1.8	-4.5	$C_{24}H_{22}N_3O_3$	$[M+H]^+$

2.34 ^{1}H NMR, ^{13}C NMR and HRMS of O7

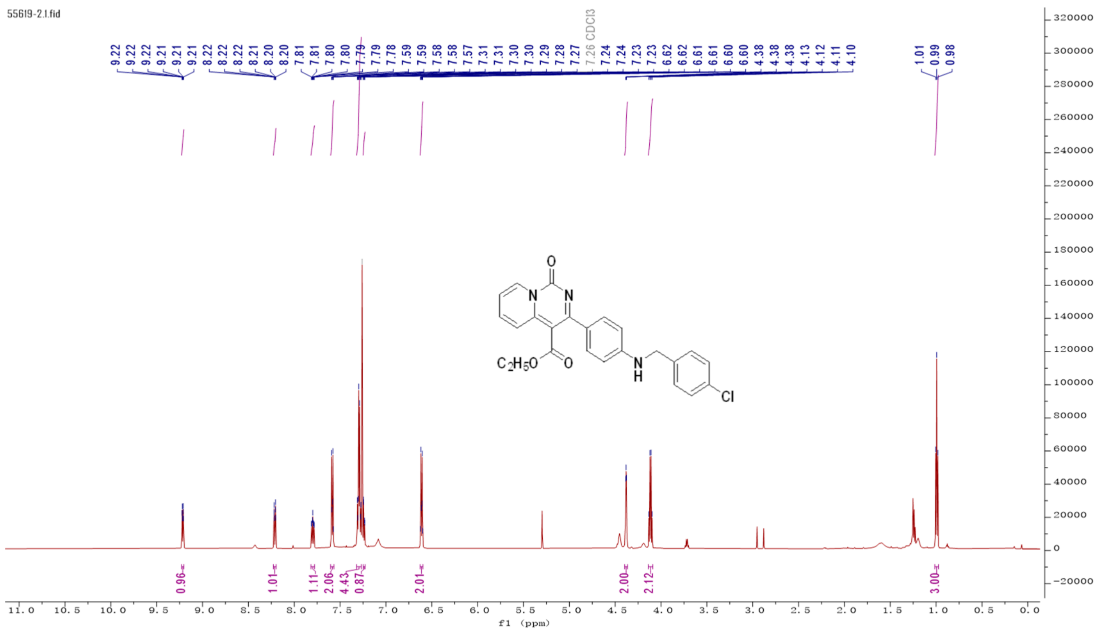
55619-2.1.fid
9.22 9.22 9.22 9.21 9.21 9.21 8.22 8.22 8.22 8.21 8.20 8.20 7.81 7.81 7.80 7.80 7.79 7.79 7.78 7.59 7.59 7.58 7.58 7.57 7.31 7.31 7.30 7.29 7.28 7.27 7.26 CDCl3 7.24 7.24 7.23 7.23 6.62 6.62 6.61 6.61 6.60 6.60 4.38 4.38 4.38 4.13 4.12 4.11 4.10 1.01 0.99 0.98
0.96 1.01 1.11 2.06 4.43 0.87 2.01 2.00 2.12 3.00
O
N
N
C_2H_5O
O
N
H
Cl
f1 (ppm)

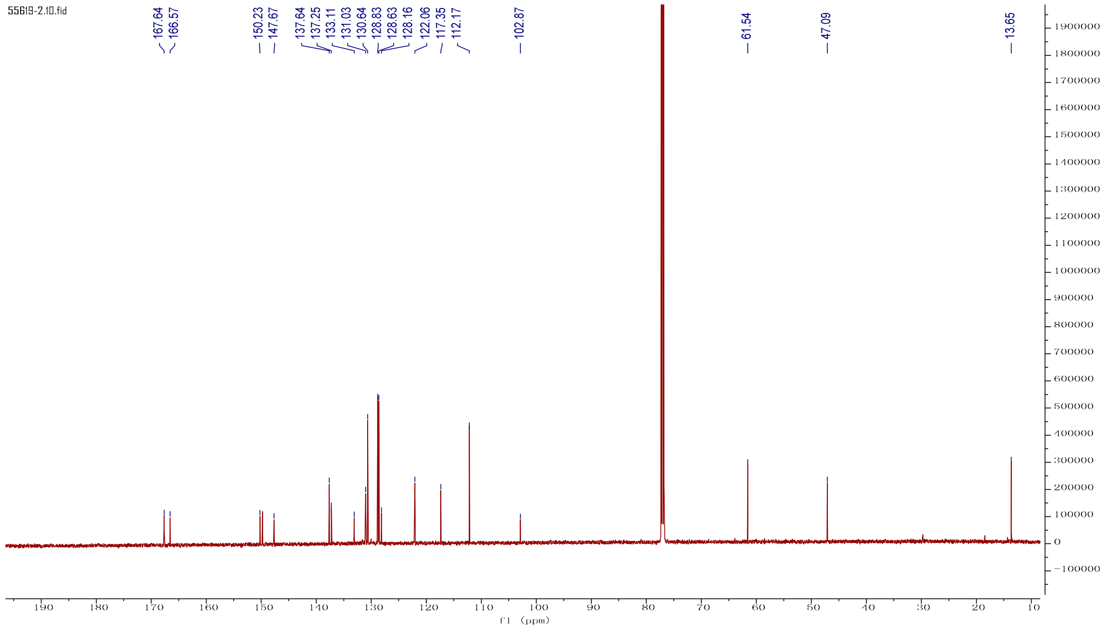
55619-2.10.fid
167.64 166.57 150.23 147.67 137.64 137.25 133.11 131.03 130.64 128.83 128.63 128.16 122.06 117.35 112.17 102.87 61.54 47.09 13.65
f1 (ppm)

MS spectra

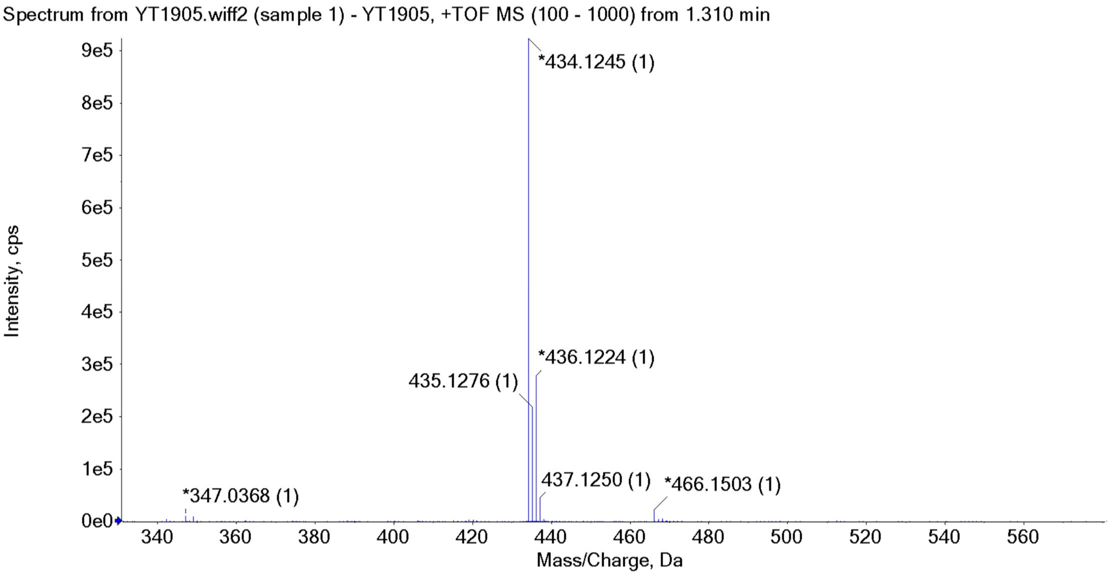

Formula Calculator Results

Measured m/z	Cal m/z	Error(mmu)	Error(ppm)	Ion Formula	Ion
434.1245	434.1266	-2.1	-5.0	$C_{24}H_{21}ClN_3O_3$	$[M+H]^+$

2.35 ^{1}H NMR, ^{13}C NMR and HRMS of P1

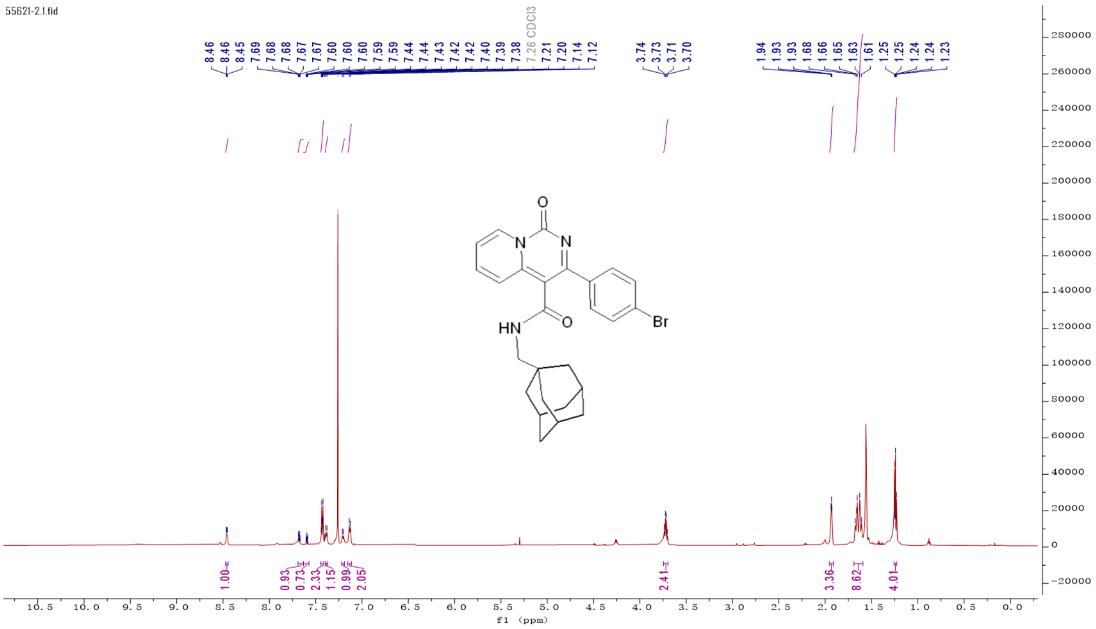

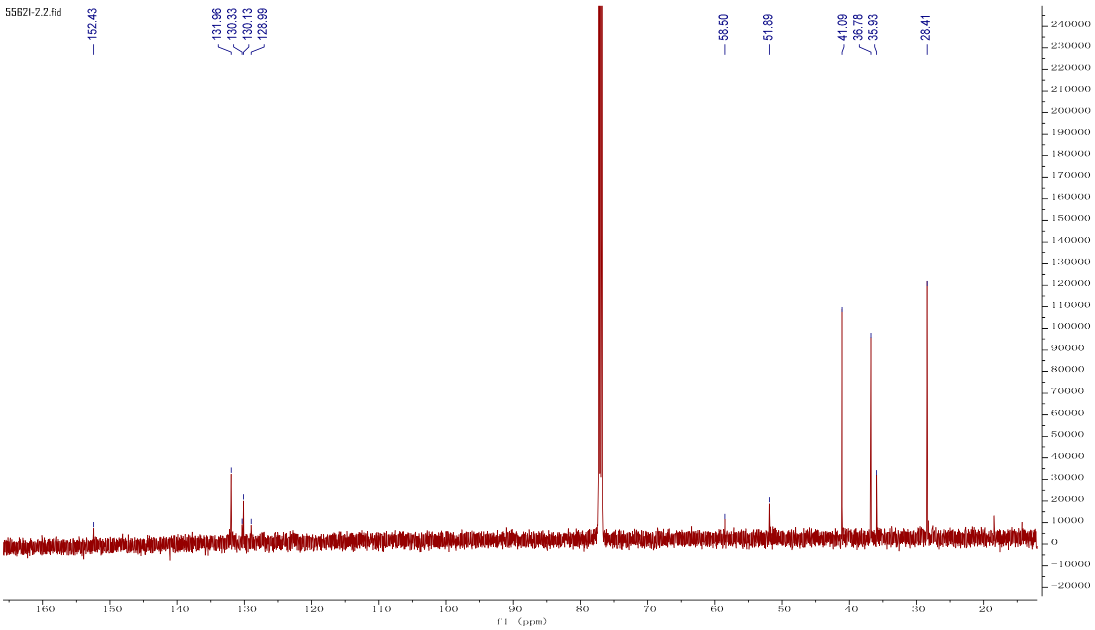

MS spectra

Spectrum from YT1907.wiff2 (sample 1) - YT1907, +TOF MS (100 - 1000) from 1.354 min

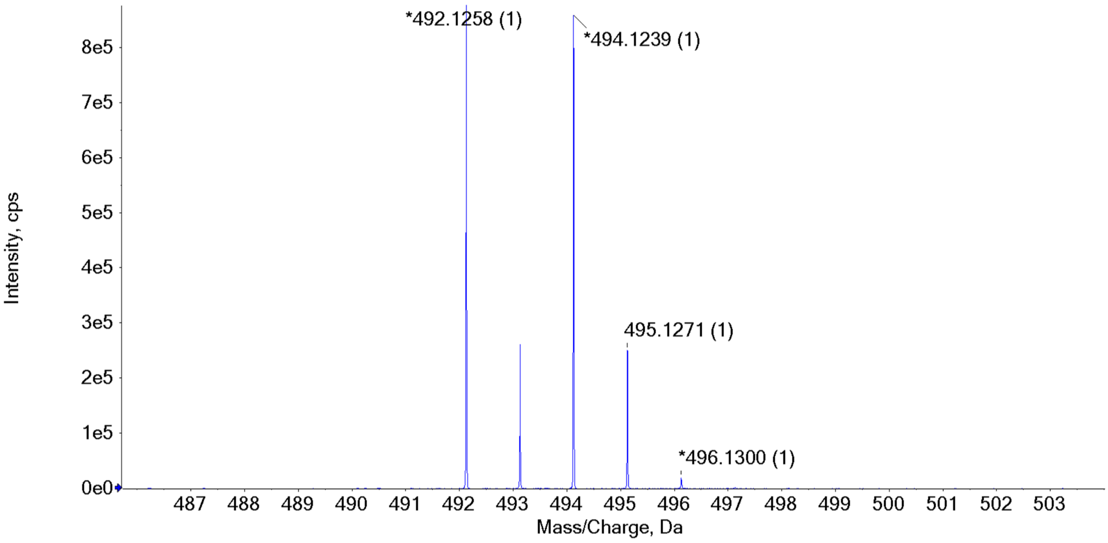

Formula Calculator Results

Measured m/z	Cal m/z	Error(mmu)	Error(ppm)	Ion Formula	Ion
492.1258	492.1281	-2.3	-4.7	$C_{26}H_{27}BrN_3O_2$	$[M+H]^+$

2.36 ^{1}H NMR, ^{13}C NMR and HRMS of P2

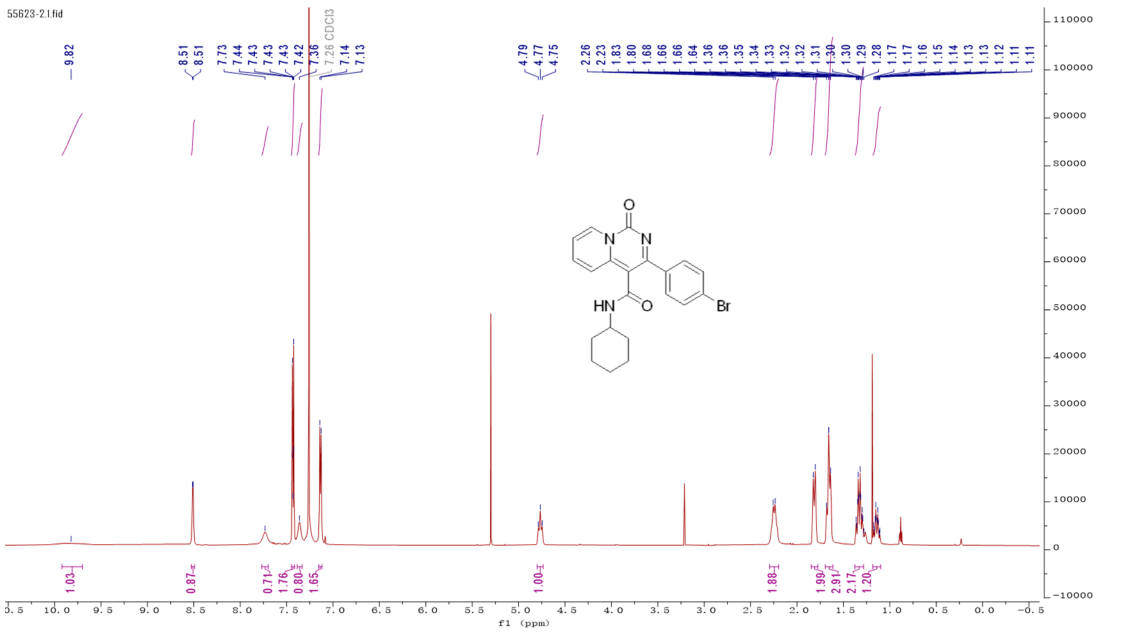

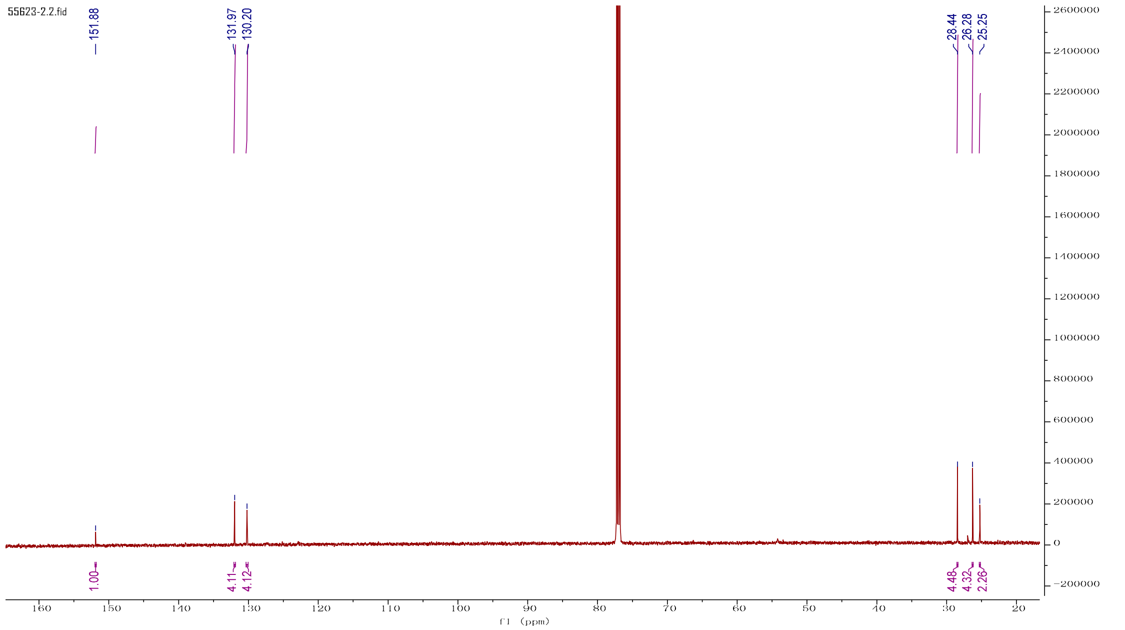

MS spectra

Spectrum from YT1910.wiff2 (sample 1) - YT1910, +TOF MS (100 - 1000) from 1.294 min

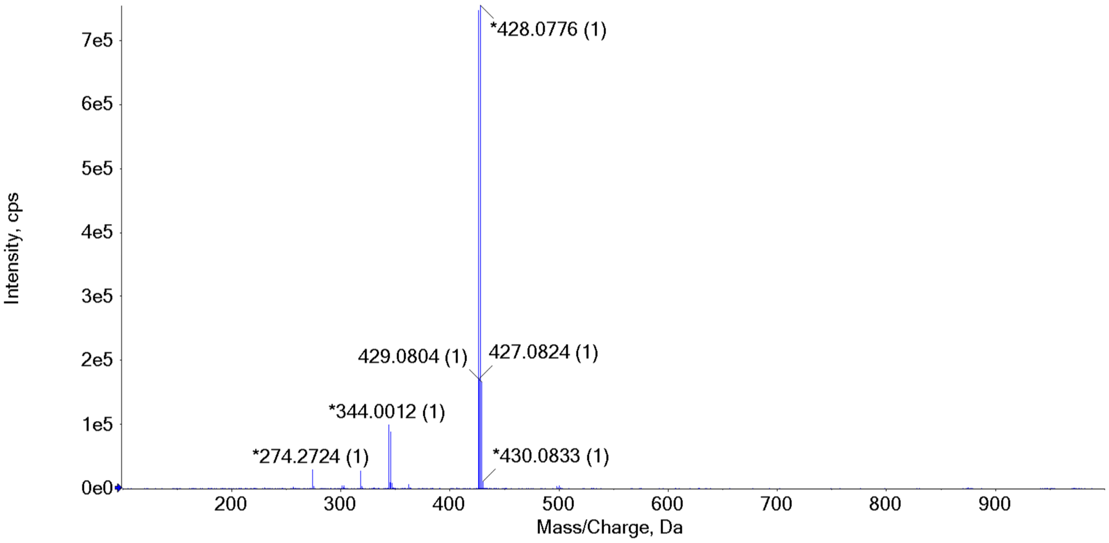

Formula Calculator Results

Measured m/z	Cal m/z	Error(mmu)	Error(ppm)	Ion Formula	Ion
426.0792	426.0812	-1.9	-4.6	$C_{21}H_{21}BrN_3O_2$	$[M+H]^+$

3.Analytical Report of HPLC Chromatograms

3.1 Analytical Report of HPLC Chromatograms of J1

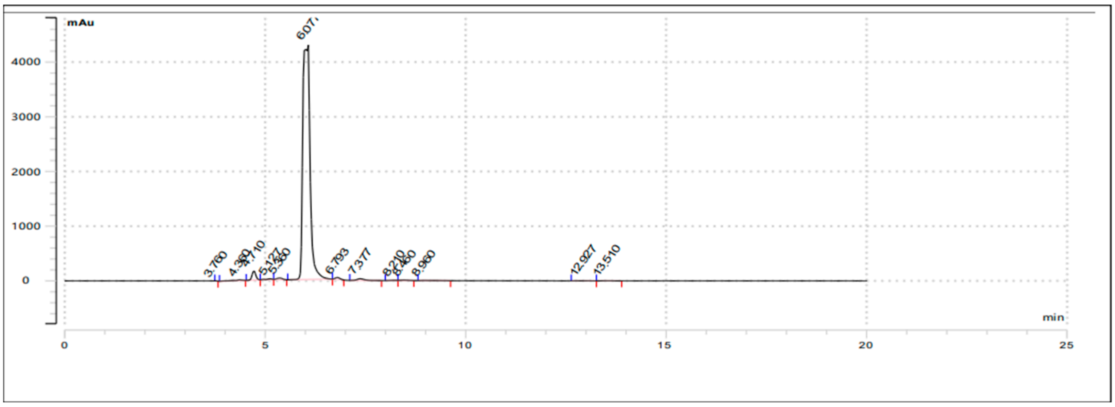

Peak List

No	Retention Time (min)	Peak Area (mAu*s)	Peak Width (min)	Half Peak Width (min)	Peak Height (mAu)	Area Percentage (%)
1	3.760	7.37540	0.111	0.066	1.214	0.013
2	4.360	222.45829	0.191	0.112	15.249	0.394
3	4.710	1084.69550	0.169	0.100	176.077	1.922
4	5.127	141.74724	0.418	0.246	14.421	0.251
5	5.360	277.84543	0.267	0.157	29.505	0.492
**6**	**6.077**	**53811.77547**	**0.319**	**0.187**	**4306.004**	**95.365**
7	6.793	294.51426	0.230	0.135	35.960	0.522
8	7.377	397.45290	0.324	0.191	31.566	0.704
9	8.210	19.73250	0.269	0.158	1.987	0.035
10	8.460	75.73200	0.313	0.184	6.625	0.134
11	8.960	68.91600	0.828	0.487	2.374	0.122
12	12.927	13.69970	0.509	0.300	0.751	0.024
13	13.510	11.11580	0.502	0.296	0.601	0.020
14		56427.06048			4622.334	100.000

3.2 Analytical Report of HPLC Chromatograms of J3

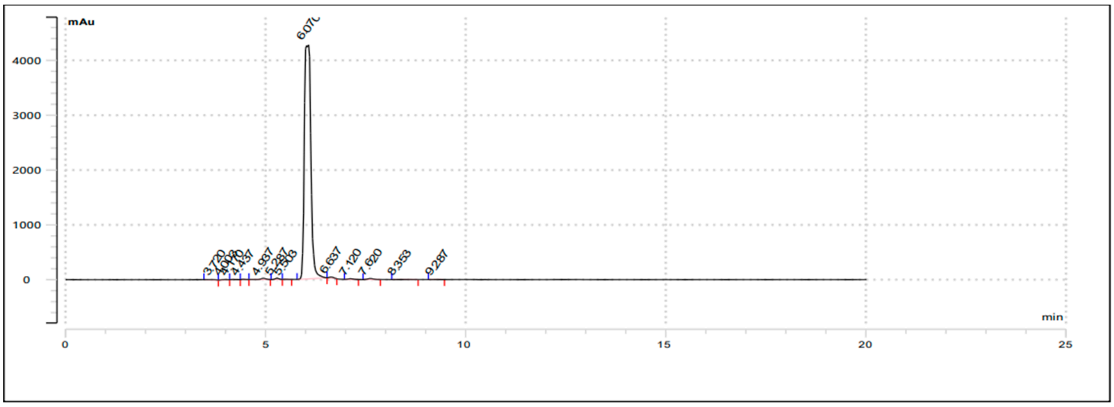

Peak List

No	Retention Time (min)	Peak Area (mAu*s)	Peak Width (min)	Half Peak Width (min)	Peak Height (mAu)	Area Percentage (%)
1	3.720	79.06915	0.344	0.203	6.503	0.160
2	4.003	60.11315	0.301	0.177	5.312	0.121
3	4.170	32.19930	0.295	0.173	3.845	0.065
4	4.437	41.23660	0.162	0.095	7.088	0.083
5	4.937	212.08015	0.217	0.128	24.911	0.428
6	5.287	158.84975	0.165	0.097	24.063	0.321
7	5.503	16.10590	0.177	0.104	2.649	0.033
**8**	**6.070**	**48402.56127**	**0.295**	**0.174**	**4292.183**	**97.729**
9	6.637	169.72465	0.208	0.122	22.542	0.343
10	7.120	120.57410	0.236	0.139	13.883	0.243
11	7.620	199.47380	0.276	0.163	19.334	0.403
12	8.353	21.72645	0.671	0.394	9.090	0.044
13	9.287	13.43940	0.325	0.191	1.128	0.027
14		49527.15366			4424.350	100.000

3.3 Analytical Report of HPLC Chromatograms of J4

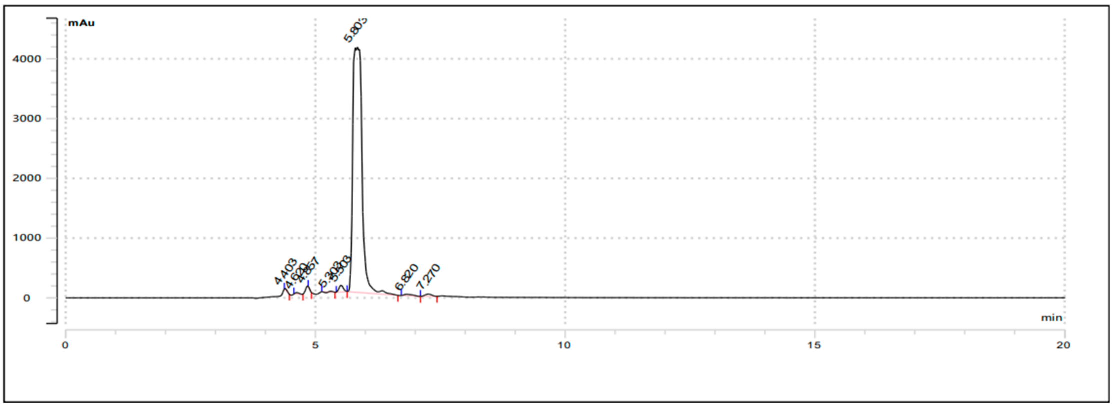

Peak List

No	Retention Time (min)	Peak Area (mAu*s)	Peak Width (min)	Half Peak Width (min)	Peak Height (mAu)	Area Percentage (%)
1	4.403	96.94203	0.074	0.044	33.624	0.178
2	4.620	119.47799	0.148	0.087	24.109	0.220
3	4.857	0.69400	0.111	0.065	0.000	0.001
4	5.303	8.81472	0.137	0.081	14.766	0.016
5	5.503	687.68030	0.157	0.092	113.702	1.263
**6**	**5.803**	**52855.43553**	**0.327**	**0.193**	**4117.103**	**97.105**
7	6.820	313.08049	0.380	0.224	25.171	0.575
8	7.270	349.00805	0.258	0.152	38.731	0.641
9		54431.13311			4367.207	100.000

3.4 Analytical Report of HPLC Chromatograms of N1

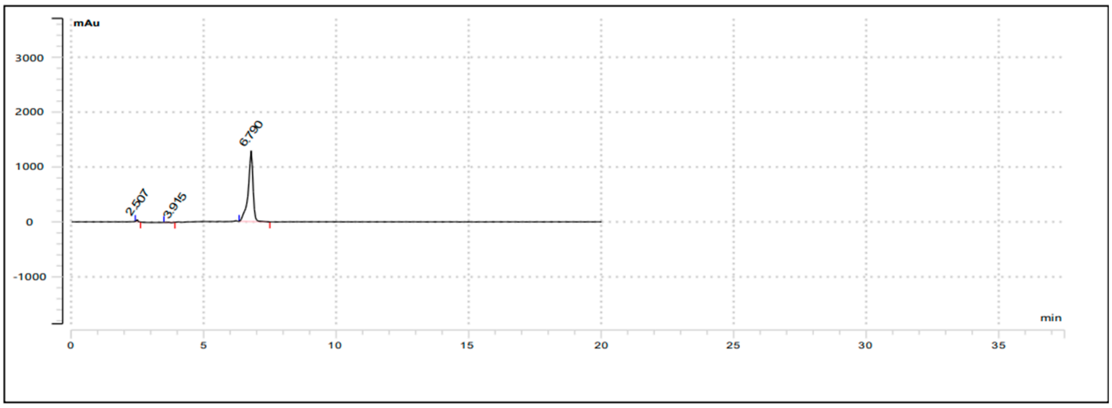

Peak List

No	Retention Time (min)	Peak Area (mAu*s)	Peak Width (min)	Half Peak Width (min)	Peak Height (mAu)	Area Percentage (%)
1	2.507	135.07900	0.075	0.044	46.815	0.822
2	3.915	52.44540	0.171	0.100	0.052	0.319
**3**	**6.790**	**16254.21132**	**0.283**	**0.166**	**1292.991**	**98.859**
4		16441.73572			1339.857	100.000

3.5 Analytical Report of HPLC Chromatograms of N2

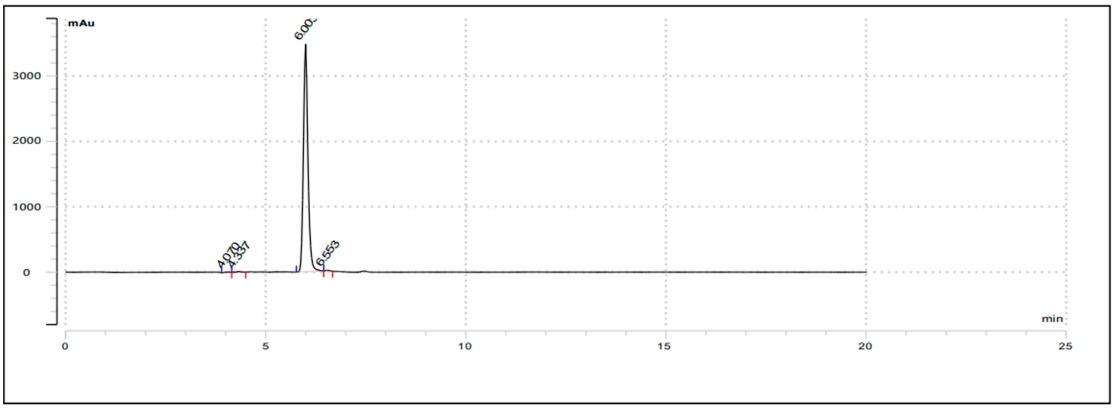

Peak List

No	Retention Time (min)	Peak Area (mAu*s)	Peak Width (min)	Half Peak Width (min)	Peak Height (mAu)	Area Percentage (%)
1	4.070	50.41780	0.302	0.178	4.076	0.184
2	4.337	56.92935	0.164	0.096	9.231	0.208
**3**	**6.003**	**27233.64877**	**0.196**	**0.116**	**3480.586**	**99.327**
4	6.553	77.23000	0.188	0.110	11.471	0.282
5		27418.22591			3505.363	100.000

3.6 Analytical Report of HPLC Chromatograms of N4

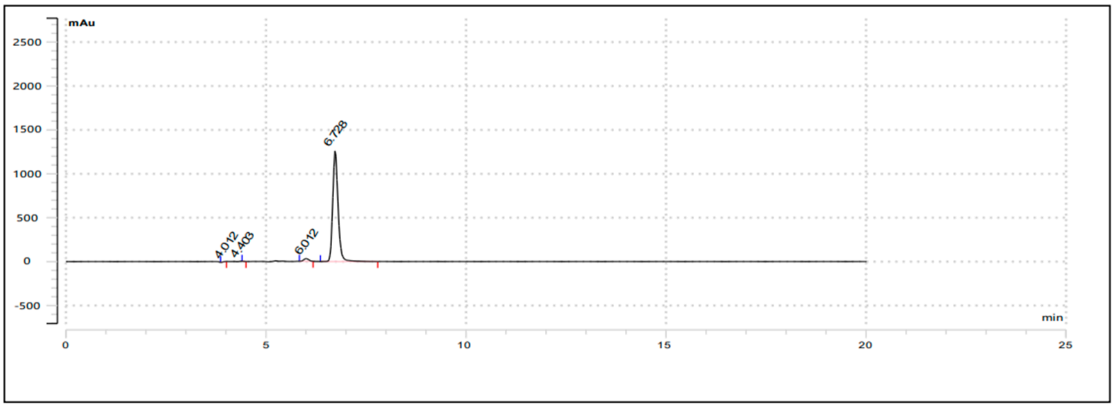

Peak List

No	Retention Time (min)	Peak Area (mAu*s)	Peak Width (min)	Half Peak Width (min)	Peak Height (mAu)	Area Percentage (%)
1	4.012	10.88455	0.197	0.116	0.091	0.091
2	4.403	1.25660	0.167	0.098	0.000	0.011
3	6.012	258.12485	0.230	0.135	30.805	2.162
**4**	**6.728**	**11666.83875**	**0.238**	**0.140**	**1269.341**	**97.736**
5		11937.10475			1300.237	100.000

4. Dose-response curve.

4.1 Dose-response curve of compound J1.

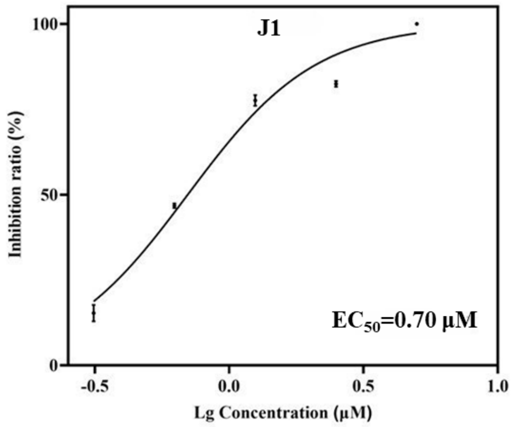

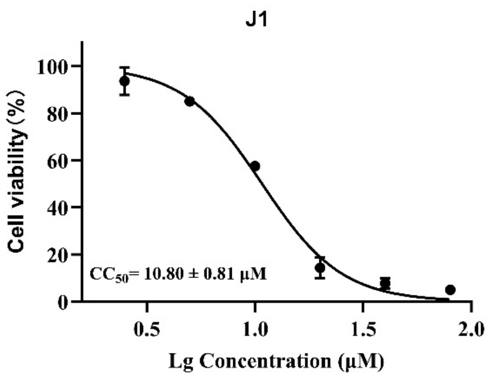

4.2 Dose-response curve of compound J3.

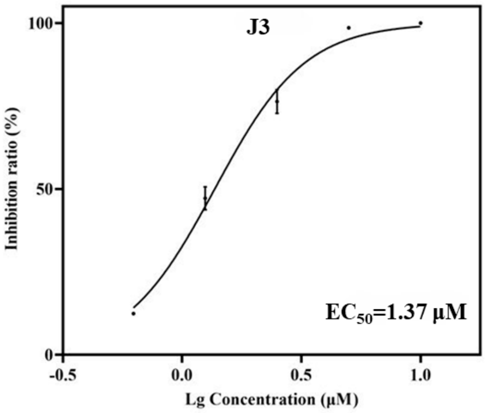

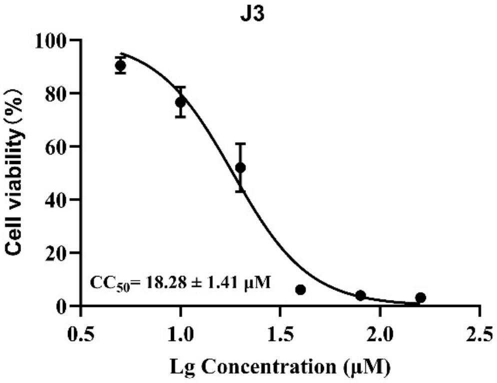

4.3 Dose-response curve of compound J4.

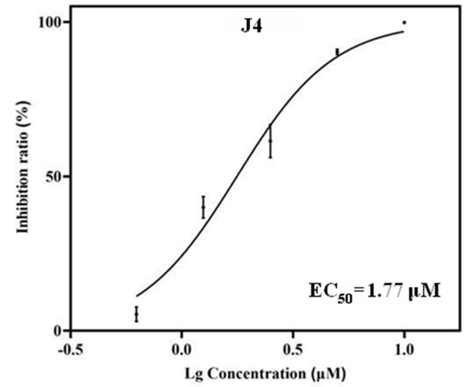

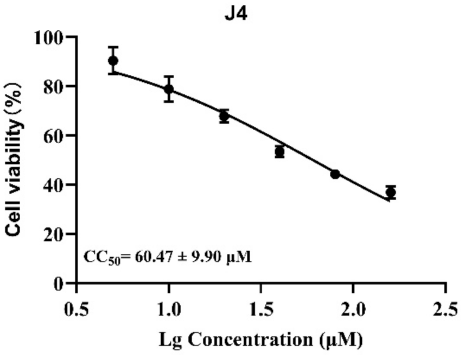

4.4 Dose-response curve of compound N1.

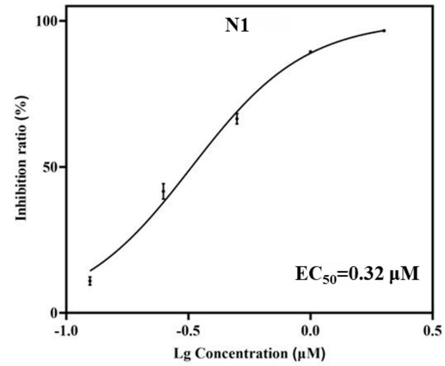

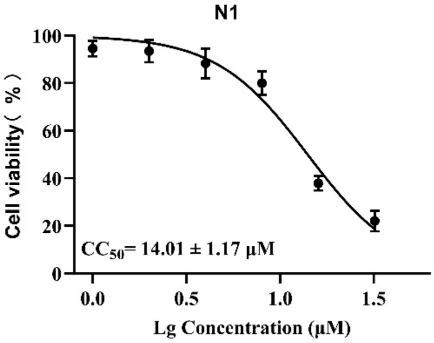

4.5 Dose-response curve of compound N2.

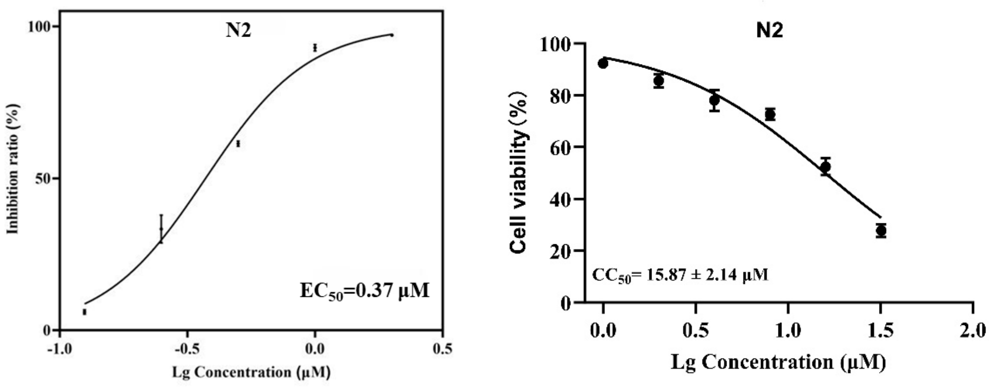

4.6 Dose-response curve of compound N4.

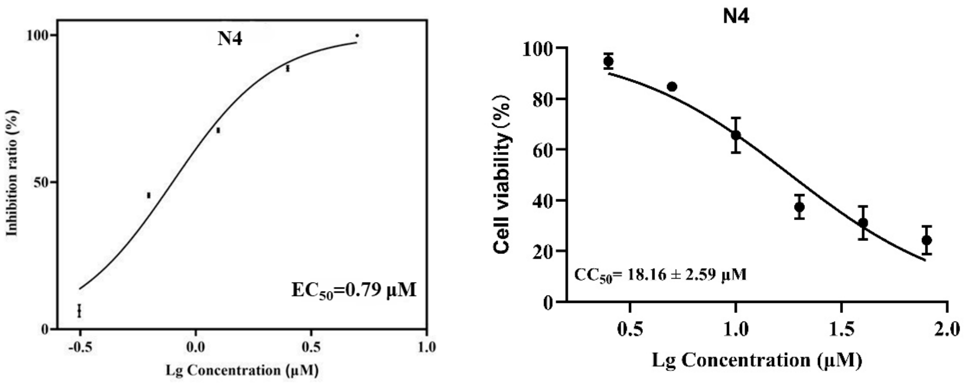

Supplement: Supplementary file 1 [file molecules-31-01480-s001.zip › molecules-4249720-supplementary.pdf]
